# Cancer-associated fibroblast heterogeneity in axillary lymph nodes drives metastases in breast cancer through complementary mechanisms

**DOI:** 10.1038/s41467-019-14134-w

**Published:** 2020-01-21

**Authors:** Floriane Pelon, Brigitte Bourachot, Yann Kieffer, Ilaria Magagna, Fanny Mermet-Meillon, Isabelle Bonnet, Ana Costa, Anne-Marie Givel, Youmna Attieh, Jorge Barbazan, Claire Bonneau, Laetitia Fuhrmann, Stéphanie Descroix, Danijela Vignjevic, Pascal Silberzan, Maria Carla Parrini, Anne Vincent-Salomon, Fatima Mechta-Grigoriou

**Affiliations:** 1 Institut Curie, Stress and Cancer Laboratory, Equipe labélisée par la Ligue Nationale contre le Cancer, PSL Research University, 26, rue d’Ulm, F-75005 Paris, France; 2 Inserm, U830, 26, rue d’Ulm, F-75005 Paris, France; 3 Analysis of Transduction Pathway, Institut Curie, Inserm, U830, PSL Research University, 26 rue d’Ulm, F-75005 Paris, France; 4 Institut Curie, Biology-inspired Physics at MesoScales Laboratory, Equipe labélisée par la Ligue Nationale contre le Cancer, CNRS UMR168, PSL Research University, Sorbonne Université, 26, rue d’Ulm, F-75005 Paris, France; 5 Institut Curie, Cell Migration and Invasion, UMR144, PSL Research University, 26, rue d’Ulm, F-75005 Paris, France; 6 Department of Pathology, Institut Curie Hospital, 26, rue d’Ulm, F-75248 Paris, France; 7 Institut Curie, Laboratoire Physico Chimie Curie, Institut Pierre-Gilles de Gennes, CNRS UMR168, 75005 Paris, France

**Keywords:** Cancer, Breast cancer, Cancer microenvironment, Tumour heterogeneity

## Abstract

Although fibroblast heterogeneity is recognized in primary tumors, both its characterization in and its impact on metastases remain unknown. Here, combining flow cytometry, immunohistochemistry and RNA-sequencing on breast cancer samples, we identify four Cancer-Associated Fibroblast (CAF) subpopulations in metastatic lymph nodes (LN). Two myofibroblastic subsets, CAF-S1 and CAF-S4, accumulate in LN and correlate with cancer cell invasion. By developing functional assays on primary cultures, we demonstrate that these subsets promote metastasis through distinct functions. While CAF-S1 stimulate cancer cell migration and initiate an epithelial-to-mesenchymal transition through CXCL12 and TGFβ pathways, highly contractile CAF-S4 induce cancer cell invasion in 3-dimensions via NOTCH signaling. Patients with high levels of CAFs, particularly CAF-S4, in LN at diagnosis are prone to develop late distant metastases. Our findings suggest that CAF subset accumulation in LN is a prognostic marker, suggesting that CAF subsets could be examined in axillary LN at diagnosis.

## Abstract (web summary)

Cancer associated fibroblasts are known to promote the progression of cancer. Here, the authors show that two particular subsets of cancer associated fibroblasts induce metastasis but work via distinct mechanisms including, chemokine signalling and Notch signalling.

## Introduction

Breast cancers (BCs) are the most frequent cancers in women worldwide. Diagnosis is mainly based on molecular subtype (Luminal A/B, HER2 and triple-negative (TN) defined according to cancer cell expression of progesterone, estrogen and HER2 receptors), tumor size and grade, as well as axillary lymph node (LN) metastasis. Most BC deaths arise from distant metastases in bone, liver and lung^1,2^. LNs are first reached by tumor cells evading from primary tumors (PTs) and number of invaded LNs is a strong prognostic factor^3^. Tumor metastasis is a multi-step process, including local invasion, intravasation, migration in blood or lymph stream, extravasation and distant organ colonization. Among others, one mechanism involves cancer cell epithelial-to-mesenchymal transition (EMT), which involves TGFβ but also NOTCH and WNT signaling pathways^4–7^.

Tumors are complex ecologies composed of numerous cell types, which participate in tumorigenesis and modulate cancer cell invasiveness^8–11^. Cancer-associated fibroblasts (CAFs) are abundant and involved in many tumor hallmarks such as angiogenesis, tumor cell proliferation, treatment resistance, immunomodulation and metastases^9,12–15^. In particular, CAFs are well-described to enhance tumor invasion and metastases, especially in BC. Indeed, CAFs are able to secrete factors, such as TGFβ and CXCL12, that directly stimulate cancer cell proliferation, EMT and migration^16–19^. Moreover, CAFs indirectly promote tumor spread via angiogenesis induction through VEGF and IL6^9,20^. Besides, CAFs can directly interact with cancer cells via heterotypic E-cadherin–N-cadherin (CDH1–CDH2) adhesion and drive their invasion^21^. They remodel the extracellular matrix (ECM)^22,23^ and produce spaces or even tracks followed by cancer cells^24–26^. Recently, lung epithelial cells were shown to acquire cancer-associated parenchymal-like feature in metastatic niche^7^.

CAFs are poorly described in metastases, including axillary LN metastases, with only few studies assessing some markers, such as podoplanin (PDPN) or α-smooth muscle actin (αSMA)^27–30^. CAFs are known to be heterogeneous in PTs^31–34^, but characterization of this heterogeneity and its link with CAF functions is still far from being understood. If the role of CAFs in tumor invasion is well established, here we investigated if and how CAF subsets act on metastatic spread. As we recently identified four CAF subsets (named CAF-S1 to -S4) in BC^32^, we investigated CAF heterogeneity in metastatic LN and tested if one or several of these CAF subsets could be involved in BC cell spread. We show here that two particular subsets, CAF-S1 and CAF-S4, strikingly accumulate in metastatic LN and positively modulate BC cell invasion by complementary mechanisms. On the one hand, CAF-S1 induce cancer cell migration and EMT initiation in a CXCL12/TGFβ-dependent manner. On the other hand, CAF-S4 contractile properties promote cancer cell motility and invasiveness in 3-dimensions (3D) through NOTCH-mediated pathways. Importantly, CAF-S1 and CAF-S4 content in LN is an independent prognostic factor at diagnosis, underlining the clinical relevance of our findings.

## Results

### Metastatic breast cancer axillary LNs exhibit four CAF subsets

**Fig. 1:**
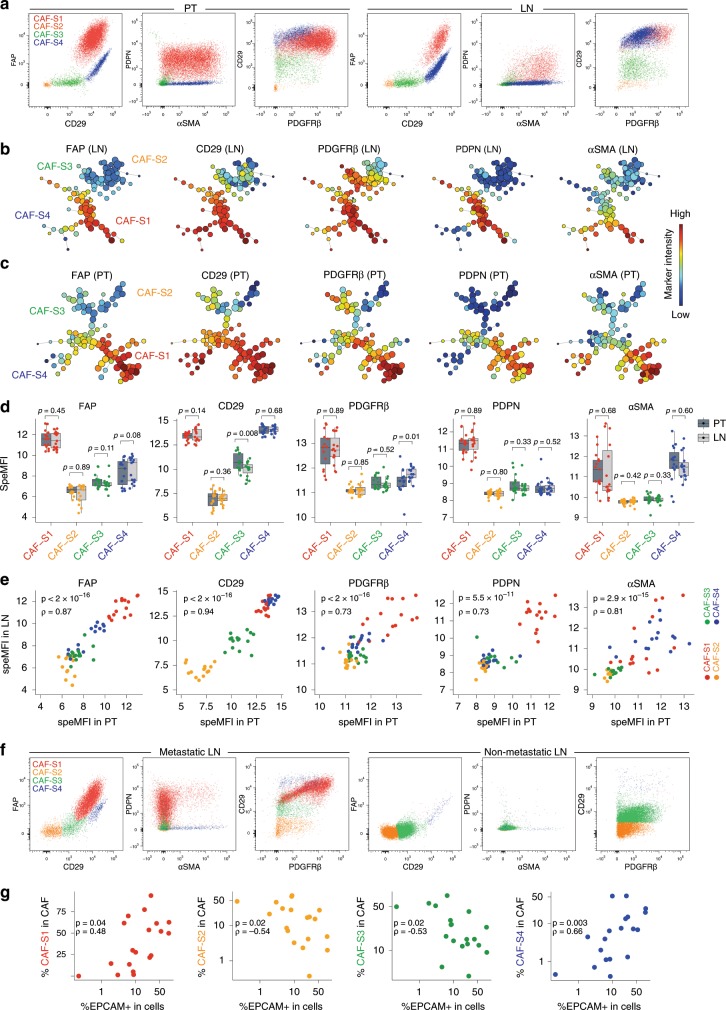
Metastatic BC LNs exhibit four CAF subsets. **a** Representative FACS plots showing FAP, CD29, PDPN, αSMA and PDGFRβ protein levels in DAPI^−^ EPCAM^−^ CD45^−^ CD31^−^ CD235a^−^ cells from a primary breast tumor (PT, left) and its corresponding metastatic axillary lymph node (LN, right). **b**, **c** FlowSom trees built on CAF from LNs (**b**, *n* = 20) and PTs (**c**, *n* = 16) and annotated for each CAF marker expression. Colors show CAF marker intensities. Node size depends on the number of phenotypically close cells. **d** Specific mean fluorescence intensity (speMFI) for each marker per CAF subset in PT and LN. Values are in log2 adjusted with offsets per marker. Each dot represents one sample (*n* ≥ 13 PT/LN pairs). Boxplots are median ± 25%–75% quantiles, whisker values range 1.5 × IQR above 75th or below 25th percentiles. *p* Values from Wilcoxon signed rank test. **e** Correlation plots between each marker speMFI in PT and LN, matched by patient and CAF subset (*n* ≥ 13 PT/LN pairs). *p* Values from Spearman’s test. **f** Same as in **a** for an invaded axillary LN (left) and its corresponding non-invaded LN (right). **g** Correlation plots between the percentage (%) of each CAF subset among total CAF and EPCAM^+^ cells among live cells, in invaded axillary LN (*n* = 19). *p* Values from Spearman’s test. Source data provided in Source Data file, with R scripts used.

To investigate CAF heterogeneity in BC metastatic axillary LNs, we first performed multicolor flow cytometry (fluorescence-activated cell sorting (FACS)) on freshly resected invaded LN, with their matched PTs as controls (Supplementary Table 1). We applied the same gating strategy on both tissues by first excluding debris, dead cells, doublets, hematopoietic (CD45+), epithelial (EPCAM+), endothelial (CD31+) and red blood cells (CD235a+) (Supplementary Fig. 1a). We then characterized the CD45− EPCAM− CD31− CD235a− fraction enriched in fibroblasts by studying five CAF markers: FAP (fibroblast activation protein α1), CD29 (Integrin β1), αSMA, PDGFRβ (platelet-derived growth factor receptor-β) and PDPN (Fig. 1a). On FACS data, we applied FlowSom^35^, an unsupervised automated algorithm which orders cells according to their phenotypic similarities (Fig. 1b, c). FlowSom clustered CAFs into four main branches and thus distinguished four CAF subsets (CAF-S1 to -S4) in invaded LNs (Fig. 1b and Supplementary Fig. 1b). Four branches were also detected in corresponding PT (Fig. 1c and Supplementary Fig. 1c), confirming data in breast and ovarian PT^32,33^. Although expression of CD29 in CAF-S3 was slightly lower and PDGFRβ in CAF-S4 slightly higher in LNs than in PTs, systematic pairwise comparison of each CAF marker protein level between LNs and PTs showed the identification of CAF-S1, -S2, -S3 and -S4 in metastatic LNs (Fig. 1d, e). Hence, these data reveal that four CAF subsets are detected in metastatic BC LNs.

As normal LN structure relies on a fibroblastic network constituted by fibroblast reticular cells (FRCs) described as PDPN+ cells^36^, we investigated the analogy between normal stromal cells and CAF subsets in LNs. Even though non-invaded LNs were hardly accessible because almost fully used for diagnosis, we had access to two non-invaded specimens (Supplementary Fig. 1d), along with their matched invaded LNs. Non-invaded axillary LNs were clearly enriched in CAF-S2- and CAF-S3-like cells, while the matched invaded LNs showed a much higher proportion of CAF-S1 and CAF-S4 (Fig. 1f and Supplementary Fig. 1e). CAF-S2 and CAF-S3 subpopulations are thus detected in metastatic LNs, but also in non-invaded LNs. These results corroborated our previous data showing that CAF-S2- and CAF-S3-like cells are detected in healthy breast tissue^32^, suggesting that these CAFs might derive from normal resident fibroblasts. In that sense, the pattern of CAF-S3 in LNs was slightly different than the one detected in PTs, as observed with CD29 staining (Fig. 1b–d), suggesting that normal-like CAF-S2/S3 could be more affected by their tissue of origin than CAF-S1 and CAF-S4. In contrast to CAF-S2 and CAF-S3, CAF-S1 and CAF-S4 were strictly observed in invaded LNs and positively correlated with tumor cell invasion (Fig. 1g). Thus, these data highlight a potential link between both CAF-S1 and CAF-S4 and tumor cell invasion in LNs. In conclusion, we identified four CAF subsets in metastatic LNs defined as: CAF-S1: FAP^High^ CD29^Med-High^ αSMA^High^ PDPN^High^ PDGFRβ^High^; CAF-S2: FAP^Neg^ CD29^Low^ αSMA^Neg-Low^ PDPN^Low^ PDGFRβ^Low^; CAF-S3: FAP^Neg-Low^ CD29^Med^ αSMA^Neg-Low^ PDPN^Low^ PDGFRβ^Low-Med^; CAF-S4: FAP^Low-Med^ CD29^High^ αSMA^High^ PDPN^Low^ PDGFRβ^Med^. Besides, the amounts of both CAF-S1 and CAF-S4 subsets in LNs are linked to BC cell metastatic spread.

### CAF-S1 and CAF-S4 are the most abundant subsets in metastatic LN

**Fig. 2:**
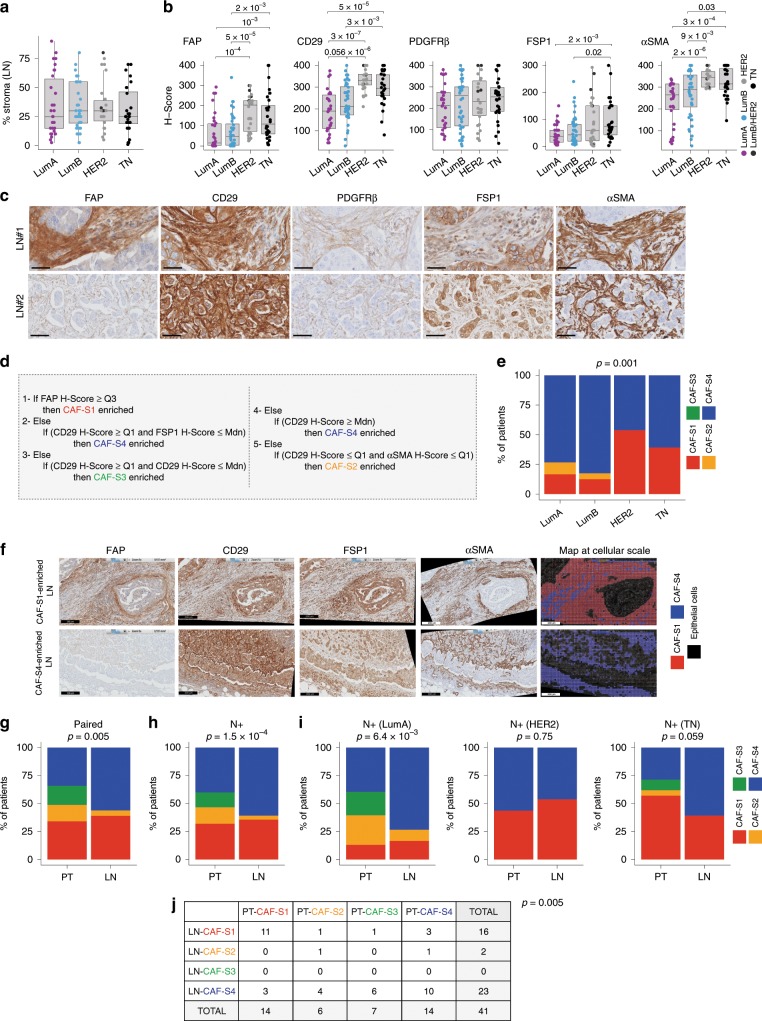
Metastatic LNs are enriched in CAF-S1 and CAF-S4. **a** Percentage (%) of stroma in invaded LN according to BC subtypes (*n* = 124), LumA (violet), LumB (blue), HER2 (light gray), Lum B/HER2 (dark gray), TN (black). **b** Histological scores (H-Scores) for each CAF marker in invaded LN according to BC subtypes (*n* = 124). **c** Representative images of CAF marker staining on serial LN sections (LN#1 HER2; LN#2 TN). Scale bar, 50 μm. **d** Decision tree defining CAF subset identity based on quartile (Q) and median (Mdn) distribution of CAF markers. Thresholds and decision rules were first established on FACS data and next applied to IHC data. **e** Repartition of CAF subset enrichments (CAF-S1 (red), CAF-S2 (orange), CAF-S3 (green) and CAF-S4 (blue)) in LN according to BC subtypes (*n* = 124). **f** Representative views of CAF marker immunostaining on serial LN sections used for building maps of CAF subsets at cellular scale using the decision tree algorithm, shown in **d**. CAF-S1 are in red, CAF-S4 in blue and epithelial cells in black. CAF-S1- and CAF-S4-enriched LNs are shown. Scale bar, 200 μm. **g** Repartition of CAF subset enrichments in PTs and matched LNs (*n* = 41 pairs). **h** Same as in **g** with unmatched samples (N+ cases, *n* = 75 PT and 84 LN). **i** Same as in **h** according to BC subtypes (Lum A: *n* = 38 PTs, 30 LNs; HER2: *n* = 16 PTs, 26 LNs; TN: *n* = 21 PTs, 28 LNs). **j** Contingency table showing repartition of CAF subset enrichments in PTs and corresponding invaded LNs (*n* = 41 pairs). In all panels, boxplots are median ± 25%–75% quantiles, whisker values range 1.5 × IQR above 75th or below 25th percentiles. **b** *p* Values from Mann–Whitney test. **e**, **g**, **h** *p* Values from Fisher’s exact test. Non-significant *p* values are not mentioned. Source data provided in Source Data file, with R scripts used.

To decipher the link between CAF subsets and metastatic spread, we studied metastatic LN sections from a retrospective cohort of 124 BC patients (Supplementary Table 2). We analyzed invaded zones of metastatic LN, identified using EPCAM marker (Supplementary Fig. 2a). We first observed that LN stroma represented around 25–30% of invaded areas, independently of BC subtypes (Fig. 2a). We performed immunohistochemistry (IHC) of five CAF markers (FAP, CD29, FSP1, PDGFRβ, αSMA) on serial LN sections (Fig. 2b, c). Here, we replaced PDPN by FSP1 because we could not find a PDPN-specific antibody for IHC, but we verified that PDPN and FSP1 markers recognized the same cells by FACS (Supplementary Fig. 2b). Histological scoring of each CAF marker demonstrated that invaded LNs from Luminal (Lum A and B) cases exhibited the lowest histological scores (H-scores) except for PDGFRβ, whereas both HER2 and TN LNs showed the highest H-scores (Fig. 2b, c and Supplementary Fig. 2c). When applying a decision tree algorithm to determine CAF subset enrichment^32^ (Fig. 2d), we found that 96% of metastatic LNs showed accumulation of CAF-S1 and CAF-S4 (Fig. 2e). Luminal LNs were mainly enriched in CAF-S4, while HER2 and TN cases displayed both CAF-S1 and CAF-S4 predominance. We observed that the median percentage of fibroblasts positive for FAP, SMA and CD29 (reflecting CAF-S1 identity) reached 75% of total CAFs in CAF-S1-enriched LNs, and that fibroblasts negative for FAP but positive for SMA and CD29 (reflecting CAF-S4 identity) reached 60% of total fibroblasts in CAF-S4-enriched LNs. We also developed an image analysis tool that combined spatial registration and computational analysis to generate maps of CAF subsets at cellular level in invaded LNs (Fig. 2f and Supplementary Fig. 2d). Representative pictures of CAF subset maps confirmed the enrichment in CAF-S1 or CAF-S4 in invaded LNs. Interestingly, among the 124 LNs analyzed, we had access to 41 corresponding PTs (Lum A, HER2, TN) for matched analyses (Fig. 2g and Supplementary Fig. 2e). We found that FAP and αSMA H-scores were similar between PTs and LNs, whereas CD29 and PDGFRβ were higher and FSP1 lower in LNs (Supplementary Fig. 2e). When we combined H-scores using the decision tree algorithm, we found that CAF enrichments in metastatic LNs were significantly different from those in matched PTs, with a strong increase in CAF-S4 in LNs (Fig. 2g). Similarly, when comparing invaded LNs to PTs (unmatched samples) from a larger BC cohort with at least one invaded LN at diagnosis (N+) (Supplementary Table 2), we confirmed the accumulation of CAF-S4, the increase in CAF-S1 and the decrease in CAF-S2 and CAF-S3 in invaded LNs compared to PTs (Fig. 2h). Metastatic LNs were particularly enriched in CAF-S4 compared to PTs in Luminal A and TN subtypes (Fig. 2i), possibly because HER2 PTs exhibited already high CAF-S4 content. As CAF marker H-scores were significantly correlated between PTs and LNs (FAP: *r* = 0.54, *p* = 3.10–4; CD29: *r* = 0.45, *p* = 3.10–3; FSP1: *r* = 0.52, *p* = 6.10–4; SMA: *r* = 0.44, *p* = 4.10–3 by Pearson’s correlation test), we hypothesized that CAF-S1-enriched tumors could give rise to CAF-S1-enriched LNs, with similar consideration for CAF-S4. Indeed, 79% of CAF-S1-enriched PTs matched with CAF-S1-enriched LNs and 71% of CAF-S4-enriched PTs matched with CAF-S4-enriched LNs (Fig. 2j) (*p* = 0.007 by Fisher's exact test). Reciprocally, CAF-S1-enriched LNs mostly matched to CAF-S1-enriched PTs (69%). In contrast, CAF-S4-enriched LNs corresponded to PTs enriched in different CAF subsets (*p* = 0.005 by Fisher's exact test), suggesting that CAF-S4 in LNs could derive from distinct CAF subsets. In conclusion, our data show that metastatic LN stroma is enriched in myofibroblastic CAF-S1 and CAF-S4 subsets. Interestingly, CAF enrichments are different in LNs compared to PTs since LN accumulating CAF-S4 can correspond to different types of PTs that are enriched either in CAF-S4 or in inactivated CAFs (CAF-S2 or CAF-S3).

### CAF subsets show similar signatures between PTs and metastatic LNs

**Fig. 3:**
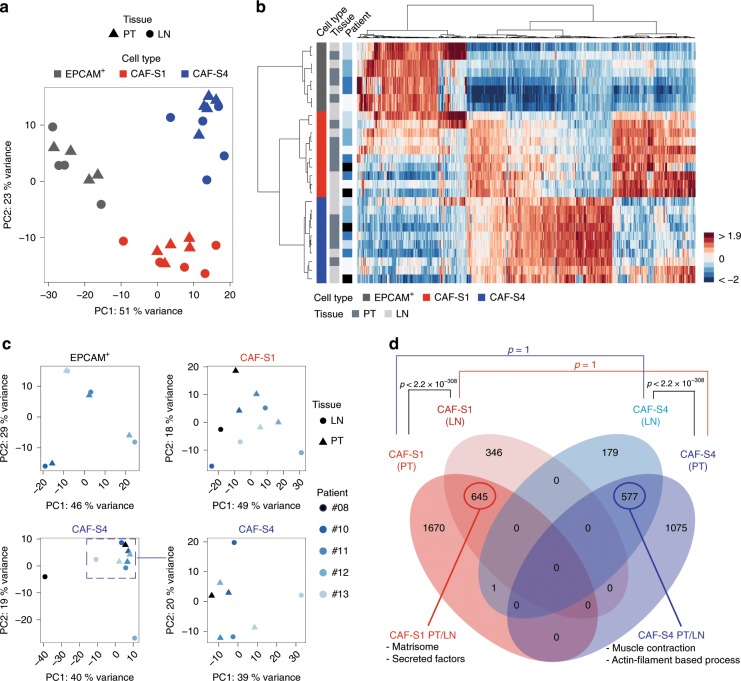
CAF subsets show same identity in PTs and metastatic LNs. **a** PCA based on the 500 most variant genes from RNAseq data of CAF-S1, CAF-S4 and EPCAM^+^ cells sorted from paired PT and LN (*n* = 5 pairs for CAF-S1 and CAF-S4; *n* = 4 pairs for EPCAM^+^ cells). **b** Hierarchical clustering on the same samples and same genes as in **a** using Ward’s method with Pearson distances. Rows represent samples and columns genes. Color saturation shows gene expression deviation from the mean (above in red, below in blue). **c** PCA based on the 500 most variant genes from RNAseq data of EPCAM^+^ cells (*n* = 4 PT/LN pairs, top left), CAF-S1 (*n* = 5 PT/LN pairs, top right) and CAF-S4 (bottom left: *n* = 5 PT/LN pairs; bottom right: PCA restricted to *n* = 8 samples, as indicated). **d** Venn diagram showing overlaps between the transcriptomic signatures of CAF-S1 from PT, CAF-S1 from LN, CAF-S4 from PT and CAF-S4 from LN. Circled numbers show common genes between tissue localization for each cell type. *p* Values from hypergeometric test indicate significance of overlaps between subgroups. Source data provided in Source Data file, with R scripts used.

As CAF-S1 and CAF-S4 subsets were largely predominant in LNs, we next aimed at characterizing these LN CAF subsets on a molecular basis and compared them to PT CAF subsets. CAF-S1 and CAF-S4 from both PTs and LNs (*n* = 5 pairs) were FACS-sorted by excluding dead, hematopoietic, epithelial, endothelial cells and erythrocytes and analyzing CAF markers (using the same gating strategy as in Supplementary Fig. 1). We performed RNA sequencing (RNAseq) on CAF-S1 and CAF-S4 fibroblasts, and EPCAM+ cancer cells sorted from PTs and matched invaded LNs (Supplementary Table 1). Unsupervised principal component analysis (PCA) and hierarchical clustering (HC) built on the 500 most variant genes distinguished tumor cells, CAF-S1 and CAF-S4 fibroblasts (Fig. 3a, b). The first PCA component (51% variance) differentiated EPCAM+ cells from CAF, and the second (23% variance) CAF-S1 from CAF-S4 (Fig. 3a). HC enabled us to visualize specific transcriptomic profiles (Fig. 3b). PCA and HC showed that samples from PTs and LNs were mixed within each cellular population, highlighting that differences between CAF subsets (CAF-S1/CAF-S4) were higher than between tissue of origin (PT/LN). We performed PCA on each population on their respective 500 most variant genes and still noted that PT and LN samples did not segregate (Fig. 3c). In line with published data^4,37^, tumor cells clustered with their matched EPCAM + LN cells, suggesting that tumor cell transcriptomes vary more between patients than between PTs and LNs. In contrast, patient effect was less detectable in CAF-S1 or CAF-S4 (Fig. 3c). We next performed paired differential analyses of CAF-S1 and CAF-S4 considering either PT or LN samples. CAF-S1 upregulated genes were mainly involved in ECM organization and CAF-S4-specific genes in muscle contraction (Supplementary Table 3 and Fig. 3d). As CAF-S4 exhibited pericyte-like signature, we confirmed that we could detect MCAM-positive fibroblasts in invaded LNs (Supplementary Fig. 2f), thereby confirming the existence of pericyte-like CAF and their potential pericyte origin^32,38–41^. Moreover, there was a strong overlap between PT and LN signatures for each CAF subset (Fig. 3d), suggesting that CAF-S1 and CAF-S4 from metastatic LN are molecularly close to CAF-S1 and CAF-S4 from PTs (Supplementary Tables 4 and 5).

### CAF-S1 and CAF-S4 exhibit distinct migration and invasion properties

**Fig. 4:**
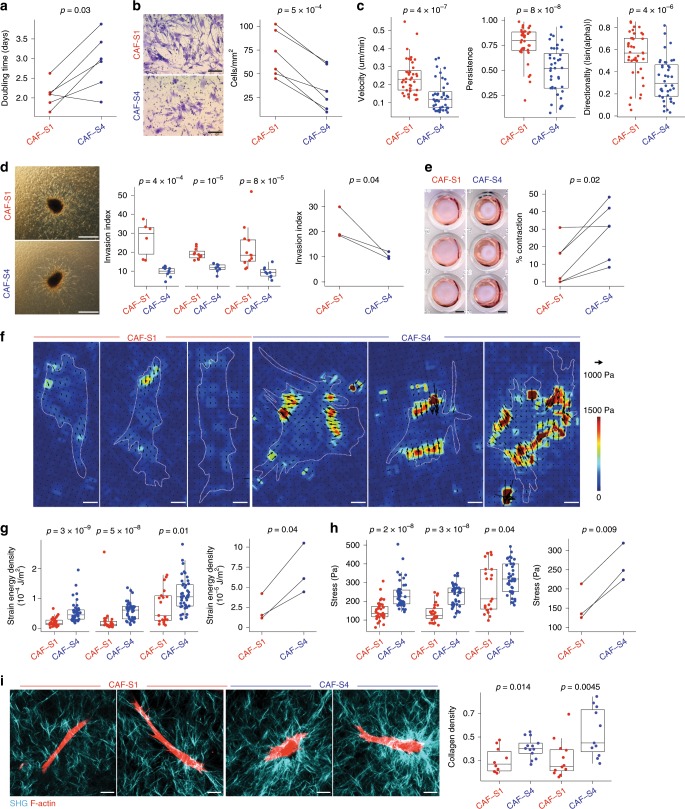
CAF-S1 and CAF-S4 exhibit distinct functional features. **a** CAF-S1 (red) and CAF-S4 (blue) doubling time (*n* = 6). **b** Left, Transwell membrane underside images. Scale bar, 200 μm. Right, CAF-S1/-S4 migration capacity (cells/mm^2^) (*n* = 6). **c** CAF-S1/-S4 velocity, persistence and direction (|sin(*α*)|, assessed by cell exclusion assay. **d** Left, Images of CAF-S1/-S4 spheroids embedded into collagen. Scale bar, 200 μm. Middle, CAF-S1/-S4 invaded areas/core spheroid areas. Each dot is one spheroid (*n* ≥ 6 per CAF subset). Right, Same as Middle for median values per CAF subset. **e** Left, Images of collagen-gel contraction by CAF-S1/-S4. Scale bar, 2 mm. Right, Percentage of collagen contraction by CAF-S1/-S4 (*n* = 6). **f**–**h** Contractility of CAF-S1/-S4 by traction force microscopy. **f** Images of traction stress applied by CAF-S1/-S4 on substrate. Traction forces (arrows) and cellular outlines (dashed lines) shown. Scale bar, 20 μm. Traction stress magnitudes in Pascal (Pa). **g** Left, CAF-S1/-S4 strain energy density (Joules (J)/m^2^). Each dot is one cell (*n* ≥ 21 cells/CAF subset). Right, Same as left for median strain energy densities per CAF subset (*n* = 3). **h** Left, CAF-S1/-S4 traction stress. Right, Same as left for median traction stress per CAF subset (*n* = 3). **i** Left, Images of collagen (blue) by CAF-S1/-S4 (red) assessed by second harmonic generation. Scale bar, 20 μm. Right, Collagen density in each cell stack. Each dot is the average value of collagen density around one cell (*n* ≥ 10 cells per CAF subset) (*n* = 2). In all panels, boxplots are median ± 25%–75% quantiles, whisker values range 1.5 × IQR above 75th or below 25th percentiles. **a**, **b**, **e** right, **g** right, **h** right: *p* Value from paired *t*-test. **c**, **g** left: *p* Values from Mann–Whitney test. **d** middle, **h** left, **i** right for two first CAF pairs: *p* Values from Mann–Whitney test (1st pair) and Student’s t-test (2nd pair). **d** right: *p* Value from Student’s *t*-test. At least three CAF-S1/-S4 pairs tested, except in **c**/**i**, two pairs. Source data provided in Source Data file, with R scripts used.

As invaded LNs show predominantly CAF-S1 and CAF-S4 fibroblasts, we hypothesized they could be involved in metastatic spread and we analyzed their properties by functional assays. We established CAF-S1 and CAF-S4 primary cell cultures from BC patients. We first confirmed by RNAseq on four CAF-S1 and CAF-S4 pairs (three couples from PTs and one from LN) that, after several passages in culture, these cells kept similar molecular identities as those detected without culture (Supplementary Fig. 3a–c). Moreover, we confirmed by FACS that CAF markers at protein levels corroborated data from fresh samples (Supplementary Fig. 3d). We next deciphered CAF-S1 and CAF-S4 intrinsic characteristics. We first observed that CAF-S1 proliferated slightly faster than CAF-S4 (median doubling time = 2.1 days for CAF-S1 and 3.0 days for CAF-S4) (Fig. 4a). Moreover, CAF-S1 displayed higher migration skills compared to CAF-S4, as assessed by Transwell assays (Fig. 4b). We validated this result using cell exclusion zone assays by manually tracking cells and calculated their velocity, persistence and directionality. CAF-S1 were faster and more persistent than CAF-S4, CAF-S1 trajectories being more perpendicular to the edges than CAF-S4 (Fig. 4c and Supplementary Fig. 3e). We also analyzed CAF subset invasion properties in 3D. Both CAF-S1 and CAF-S4 subsets were able to form spheroids in hanging drops and to invade the surrounding matrix but CAF-S1 were more efficient in invading than CAF-S4 (Fig. 4d). Interestingly, on time-lapse video microscopy (Supplementary Movies), we observed that spheroids made by CAF-S4 pulled more efficiently on collagen fibers than CAF-S1 (Fig. 4e). To further quantify the traction forces exerted by CAF on matrix, we performed traction force microscopy (TFM). We observed that CAF-S1 were more elongated compared to CAF-S4 that were larger (Fig. 4f and Supplementary Fig. 3f, h). Moreover, the strain energy (that measures the energy spent to deform the substrate) developed by CAF-S4 was more than twofold higher than that of CAF-S1 (Supplementary Fig. 3g). To take into account differences in surface area between CAF-S1 and CAF-S4 (Supplementary Fig. 3h), we compared the strain energy densities (strain energy normalized to the cell surface) and confirmed that CAF-S4 deployed more contractile energy per surface unit than CAF-S1 (Fig. 4g). Similar results were obtained using mean traction stress amplitude (mechanical forces cell develop per unit area to deform the substrate; arrows Fig. 4f, h). Collagen fiber density was also increased around CAF-S4 compared to CAF-S1 cells (Fig. 4i), which is consistent with CAF-S4 capacity to exert higher traction forces on their environment, to pull and contract collagen fibers. Overall, these data demonstrate that CAF-S1 are motile, while CAF-S4 are contractile with strong capacity to pull on collagen fibers.

### CAF-S1 and CAF-S4 promote tumor cell spread by distinct mechanisms

**Fig. 5:**
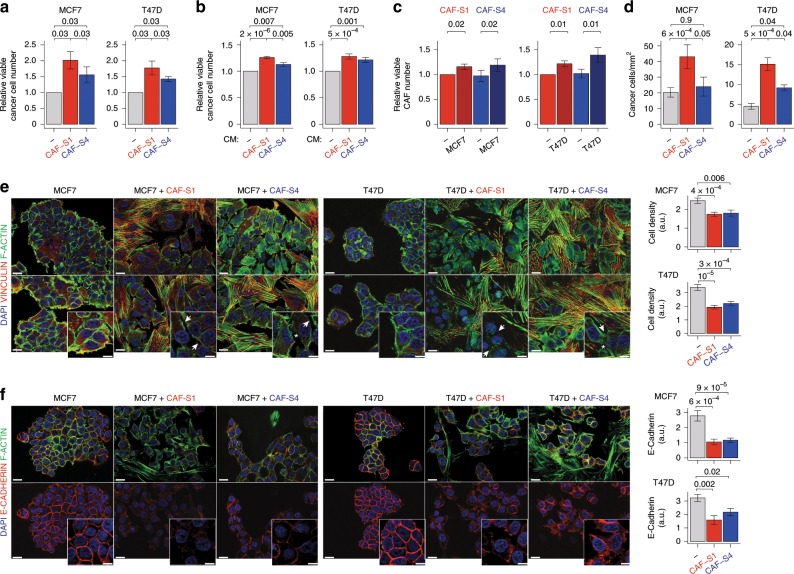
CAF-S1 promote proliferation and initiate cancer cell epithelial-to-mesenchymal transition. **a** Total number of viable BC cells (Dapi^−^ cells by FACS) in co-culture with CAF-S1 (red) or CAF-S4 (blue) relative to control (− gray, without CAF) (*n* = 6 per BC cell type). **b** Total number of viable BC cells (Resazurin staining) with CAF-S1- or CAF-S4-conditioned medium (CM) relative to control (−, without CM) (*n* = 9 per BC cell type). **c** Total number of viable CAF-S1 (red) and CAF-S4 (blue) (Dapi^−^ cells by FACS) in co-culture with BC cells relative to control (−, without BC cells) (*n* ≥ 6 per BC cell type). **d** CAF-S1 and CAF-S4 capacities to attract BC cells (*n* ≥ 8 per BC cells). **e** Left, Images of co-staining of Vinculin (red), F-actin (green) and DAPI (blue) in MCF7 (left) or T47D (right) cultured alone, or in presence of CAF-S1 or CAF-S4. Scale bars, 20 μm; inset, 10 μm. Arrows show reduced BC cell cohesion in presence of CAF-S1/-S4; asterisks show F-actin interconnections between BC cells in presence of CAF-S4. Vinculin and F-actin individual staining in Supplementary Fig. 4c. Right, Number of BC cells per tumor area (at least 13 images per condition, *n* = 3 per BC cell type). **f** Left, Images of E-cadherin (red), F-actin (green) and DAPI (blue) co-staining (top) or of E-cadherin (red) and DAPI (blue) staining (bottom) in BC cells alone or in presence of CAF-S1/-S4. Scale bars, 20 μm; inset, 10 μm. Right, Quantification of E-cadherin staining per BC cell area (at least eight images per condition) (*n* = 2 per BC cell type). In all panels, barplots are mean ± SEM and *n* number of independent experiments; a. u., arbitrary units. **a**, **d**: *p* Values from Wilcoxon signed rank test. **b**, **c**: *p* Values from paired *t*-test. **e** right, **f** right: *p* Values from Student’s *t*-test (MCF7) and Mann–Whitney test (T47D). At least two CAF-S1 and CAF-S4 pairs tested, except in **e**, **f** for T47D, where one CAF-S1/-S4 pair is used. Source data provided in Source Data file, with R scripts used.

While CAF function in metastases was investigated in the past, the specific role of CAF subsets on metastatic spread remains unknown. We assessed CAF-S1 and CAF-S4 effects on BC cell properties by using MCF7 and T47D luminal cell lines, as CAF switch between PTs and LNs was prominent in luminal cases. Co-culture of BC cells with CAF-S1 or CAF-S4 increased the number of tumor cells, with a stronger impact of CAF-S1 compared to CAF-S4 (Fig. 5a). This effect was also detected with CAF-S1- or CAF-S4-conditioned medium (CM), although at a lower extent (Fig. 5b). CAF subset proliferation increased at long time points of co-culture with cancer cells (Fig. 5c), but not at short term (Supplementary Fig. 4a). Transwell assays showed that BC cell migration was strongly increased toward CAF-S1, but at a much lower extent toward CAF-S4 (Fig. 5d). The CAF-S1-pro-migratory effect was independent of its pro-proliferative effect, while the slight impact of CAF-S4 was proliferation-driven (Supplementary Fig. 4b). In agreement with their intrinsic features (Fig. 4), CAF-S4 fibroblasts (easily distinguished from cancer cells by F-actin staining) exhibited more F-actin stress fibers and Vinculin-stained focal adhesions than CAF-S1 (Fig. 5e and Supplementary Fig. 4c for quantification). Interestingly, in presence of CAF-S1 or CAF-S4, cancer cells were more scattered (Fig. 5e and Supplementary Fig. 4c). Indeed, in presence of CAF-S1, BC cells were less cohesive and more dispersed. With CAF-S4, cancer cells were also less packed but with numerous remaining F-actin interconnections. In agreement with these observations, E-cadherin/CDH1 protein levels decreased at cancer cell membrane in presence of CAF-S1, effect which was also visible but less pronounced with CAF-S4 (Fig. 5f and Supplementary Fig. 4d). Thus, these results suggest that CAF-S1–and to a lower extent CAF-S4—promote cancer cell spreading by initiating first steps of EMT.

**Fig. 6:**
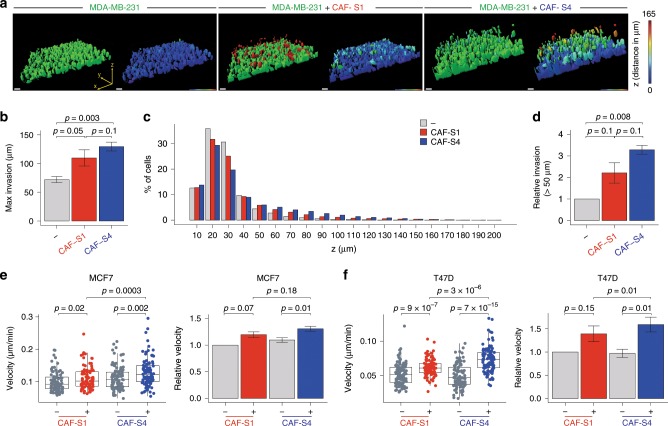
CAF-S4 promote tumor cell invasion in 3D. **a** Representative 3D views of MDA-MB-231 invasion assessed by inverted Transwell assays, in CAF-free (left), CAF-S1- (middle) or CAF-S4- (right) embedded collagen matrix. In left images in each condition, colors show the different cell types: BC cells in green; CAF-S1 in red; CAF-S4 in blue. In right images in each condition, colors indicate the distance browsed by BC cells on the vertical (*z*) axis in 3D (maximal distance 165 μm in red). Scale bar, 50 μm. **b** Maximal vertical distance browsed by BC cells in CAF-free, CAF-S1- or CAF-S4-embedded collagen (*n* = 3, ~500 BC cells per analyzed *z*-stack). **c** Mean frequency (%) of BC cells along vertical axis (*z*, μm) in CAF-free, CAF-S1- or CAF-S4-embedded collagen. **d** Proportion of BC cells that invaded above 50 μm in CAF-S1- or CAF-S4-embedded collagen, relative to CAF-free condition (*n* = 3, ~500 BC cells per analyzed *z*-stack). **e** Left, Representative tumor-on-chip experiment showing velocity of MCF7 in CAF-free, CAF-S1- or CAF-S4-embedded collagen. Each dot represents one cell (*n* ≥ 90 cells per condition). Right, Median velocity of MCF7 in CAF-S1- or CAF-S4-embedded collagen matrix relative to CAF-free condition (*n* = 3). **f** Same as in **e** for T47D. In all panels, barplots are mean ± SEM. Boxplots are median ± 25%–75% quantiles, whisker values range 1.5 × IQR above 75th or below 25th percentiles; *n* indicates number of independent experiments. **b**, **d**, **e** right: *p* Values from paired *t*-test. **e** left: *p* Values from Mann–Whitney test. At least two CAF-S1 and CAF-S4 pairs have been tested. Source data provided in Source Data file, with R scripts used.

As CAF-S4 properties were linked to 3D environments, we moved to such systems to analyze their impact on BC cell invasiveness in surrounding matrix. We first used an inverted Transwell assay to test CAF subset-mediated matrix remodeling on BC cell invasion (Fig. 6a–f). In this assay, cancer cells need to cross the membrane to invade the matrix. MCF7 and T47D cells are not invasive enough to obtain quantifiable results, we thus chose to work with MDA-MB-231, a TN invasive cell line. The highest *z*-distance covered by cancer cells in the matrix was significantly increased in CAF-S1- and even more in CAF-S4-embedded collagen (Fig. 6a, b). If cancer cells browsed in average 72 μm above the membrane in a CAF-free matrix, they reached 109 μm in presence of CAF-S1 and 129 μm with CAF-S4 (Fig. 6b). We next looked at the average distribution of cancer cell frequency along *z*-axis, every 10 μm from the membrane up to 200 μm. Major part of cancer cells was below 30 μm, distance from which a major drop was observed (Fig. 6c). Interestingly, beyond 30 μm, CAF-S4 significantly increased the proportion of cancer cells compared to control (Supplementary Fig. 4e, left). Similar results were obtained when increasing the invasion threshold, with an even greater impact of CAF-S4 on tumor cell invasion (Fig. 6d and Supplementary Fig. 4e, right). To go deeper in the characterization of BC cell motility in 3D, we took advantage of the tumor-on-chip system^42^, a microfluidic device where cancer cells and CAF subsets were co-cultured in 3D and followed by time-lapse video microscopy. In this system, we reproduced the intrinsic CAF subset motility, CAF-S1 being more motile than CAF-S4 (Supplementary Fig. 4f). In addition, velocity of GFP-labeled BC cells increased in presence of CAF-S4, and at a lower extent CAF-S1 (Fig. 6e, f). In tumor-on-chip, CAF subset-mediated effect was more important on T47D (Fig. 6f) than MCF7 (Fig. 6e), probably because T47D cells are less motile at basal levels than MCF7 (around 0.1 μm/min for MCF7 and 0.05 μm/min for T47D). Taken together, these results show that both CAF-S1 and CAF-S4 stimulate BC cell motility, via different effects: CAF-S1 initiate first steps of EMT and secrete factors that attract cancer cells, while CAF-S4 remodel the matrix and promote cancer cell invasion in 3D.

### CAF-S1 and CAF-S4 induce cancer cell invasion by TGFβ/CXCL12 and NOTCH

**Fig. 7:**
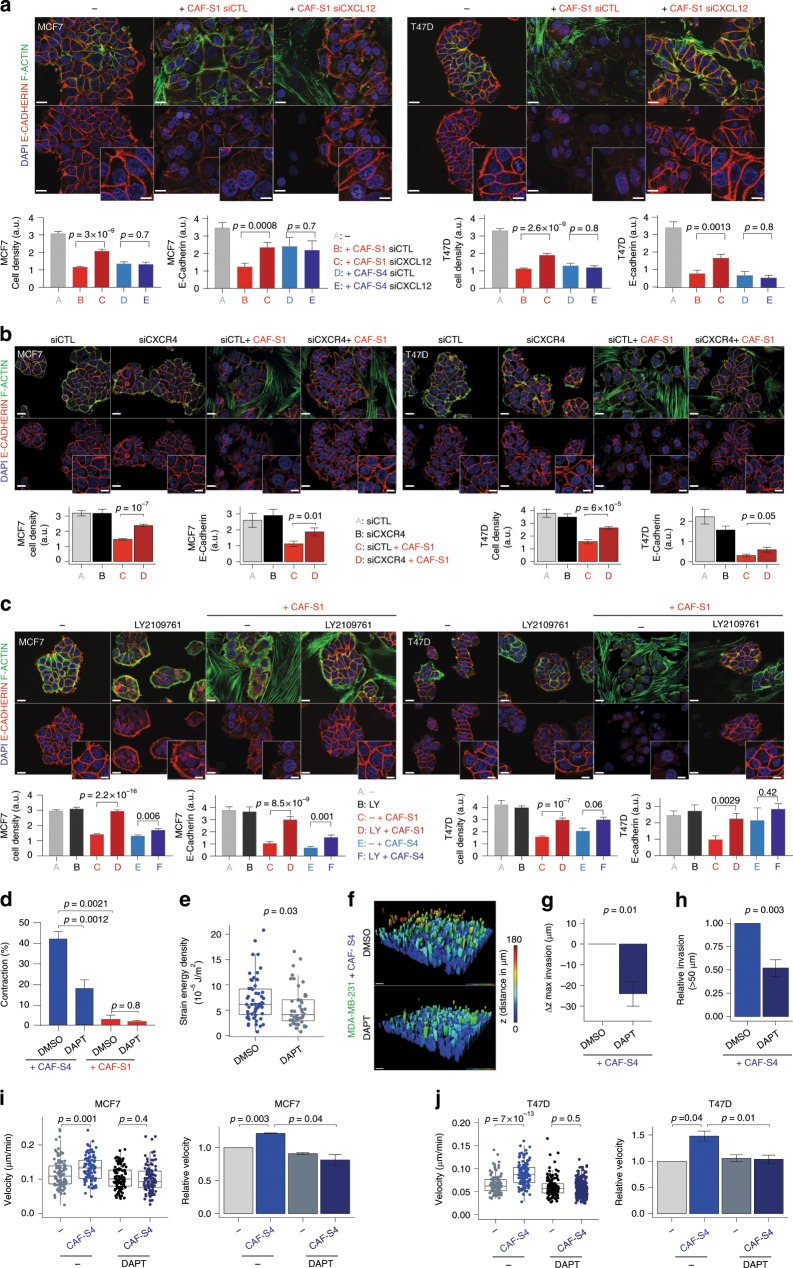
CAF-S1 and CAF-S4 promote cancer cell invasion by TGFβ, CXCL12 and NOTCH. **a** Up, Representative images of E-cadherin (red), F-actin (green) and DAPI (blue) in BC cells alone (−) or with CAF-S1 transfected with non-targeting (siCTL) or CXCL12-targeting (siCXCL12) siRNA. CAF-S4 images in Supplementary Fig. 5c. Scale bars, 20 μm; inset 10 μm. Down, BC cell density and E-cadherin staining alone (gray) or with CAF (CAF-S1 red; CAF-S4 blue) (≥12 images/condition; *n* = 8). **b** Same as **a** with siCTL- or siCXCR4-transfected BC cells, with/without CAF-S1 (red/gray) (≥8 images/condition; *n* = 4). **c** Same as **a** with/without CAF-S1 with/without TGFβ-R inhibitor (LY2109761). CAF-S4 images in Supplementary Fig. 5e. Quantifications without (gray) or with CAF (CAF-S1 red; CAF-S4 blue) (≥7 images/condition; MCF7: *n* = 7; T47D: *n* = 3). **d** Percentage (%) of collagen contraction by CAF-S4 (blue) or CAF-S1 (red) without (DMSO) or with DAPT (*n* ≥ 3). **e** Strain energy density of CAF-S4 without (DMSO, blue) or with DAPT (gray). Each dot is one cell, *n* ≥ 46 cells/condition. **f** Representative 3D views of MDA-MB-231 (green) invasion by inverted Transwell assays in CAF-S4 (blue)-embedded collagen matrix without (DMSO) or with DAPT. Colors indicate distance browsed by BC cells on *z* axis (*d*_max_180 μm, red). Scale bar, 50 μm. **g** Maximal distance of BC cells in CAF-S4-embedded collagen with DAPT relative to control (DMSO) (*n* = 6, ~700 BC cells/*z*-stack). **h** Same as in **g** for proportion of BC cells that invaded above 50 μm. **i** Left, Velocity of MCF7 in tumor-on-chip in CAF-free (−, gray) or CAF-S4 (blue)-embedded collagen treated or not with DAPT. Each dot is one cell, *n* ≥ 95/condition. Right, Same as left for median velocity of MCF7 (*n* = 3). **j** Same as **i** for T47D. In all panels, boxplots are median ± 25%–75% quantiles, whisker values range 1.5 × IQR above 75th or below 25th percentiles. Barplots mean ± SEM. **a**–**e** *p* Values from Mann–Whitney test. **g**–**i** *p* Values from paired *t*-test. At least four CAF-S1 and CAF-S4 tested. Source data provided in Source Data file, with R scripts used.

We next sought to define the molecular actors involved in CAF-S1 and CAF-S4 pro-invasive effects. As CAF-S1 attract cancer cell at distance and secrete high levels of CXCL12 (Supplementary Fig. 5a), we first analyzed the impact of CXCL12 silencing in CAF-S1 and CAF-S4 (Supplementary Fig. 5b) on BC cell proliferation and migration. Interestingly, CAF-S1 silenced for CXCL12 partly lost their ability to initiate EMT in cancer cells, while CXCL12-silencing had no impact in CAF-S4 (Fig. 7a). Indeed, BC cells were more clustered and E-cadherin protein levels in tumor cells increased after CXCL12 silencing in CAF-S1 but not in CAF-S4 (Fig. 7a and Supplementary Fig. 5c). We also observed that CXCL12-silencing in CAF-S1 decreased BC cell chemo-attraction without affecting their proliferation rate (Supplementary Fig. 5d, e). In agreement with these observations, even partial inhibition of CXCR4 in cancer cells (Supplementary Fig. 5f) affected their scattering and EMT initiation by CAF-S1 (Fig. 7b). This suggests that both CXCL12 secretion by CAF-S1 and CXCR4 expression in cancer cells are involved in CAF-S1-mediated EMT initiation in BC cells. As we observed an upregulation of the TGFβ-signaling pathway in CAF-S1 (Supplementary Fig. 5g), we wondered whether this pathway, strong EMT inducer, could also be involved in CAF-S1-mediated EMT initiation. As the expression of TGFβ receptors (TGFBR1, 2 and 3) was upregulated in CAF-S1 cells compared to CAF-S4 (Supplementary Fig. 5h), we compared the impact of TGFBR inhibitor on CAF-S1- and CAF-S4-induced EMT initiation on cancer cells (Fig. 7c and Supplementary Fig. 5i). TGFBR inhibition affected initiation of EMT in cancer cells in presence of CAF-S1 cells, while it had no impact on cancer cells alone and a weak effect with CAF-S4 (Fig. 7c). Taken together, these results show the role of TGFβ and CXCL12 signaling pathways in CAF-S1-dependent pro-tumorigenic phenotype.

Concerning CAF-S4, NOTCH pathway and three out of the four NOTCH receptors (NOTCH 1–3) were significantly upregulated in CAF-S4 (Supplementary Fig. 6a, b). We observed that DAPT, a γ-secretase inhibitor and pan-NOTCH inhibitor, severely reduced CAF-S4 contractile capacity assessed in collagen gel assays, while DAPT had no impact on CAF-S1 fibroblasts (Fig. 7d). Due to this strong effect of NOTCH inhibition on CAF-S4 contractility, we next analyzed in-depth the effect of NOTCH on CAF-S4. We verified that DAPT had no effect on CAF-S4 viability (Supplementary Fig. 6c). As expected, Blebbistatin and Y27632, potent myosin and ROCK inhibitors used as positive controls, also abolished CAF-S4 contraction (Supplementary Fig. 6d). Using TFM, we confirmed that DAPT treatment also diminished the Strain Energy Density globally exerted by CAF-S4 cells (Fig. 7e), although it did not significantly affect the magnitude of the stress which averages the local traction forces (Fig. S6e). Moreover, the inverted Transwell invasion assay confirmed the role of NOTCH in CAF-S4-mediated BC cell invasion. Indeed, both the maximal distance reached and the percentage of invading tumor cells were reduced by NOTCH inhibition in CAF-S4-embedded matrix (Fig. 7f, h), while it had no or much lower impact without CAF-S4 (Supplementary Fig. 6f, g). Finally, using tumor-on-chip devices, we further showed that, although cancer cell motility was not affected by DAPT treatment in absence of CAF-S4, CAF-S4 were completely unable to induce BC cell invasion in presence of DAPT (Fig. 7i, j). Altogether, these data demonstrate that NOTCH signaling in CAF-S4 is essential to remodel the matrix and promote BC cell invasion in 3D. Finally, by using a cytokine array, we verified that the release of other chemokines and interleukins, such as IL6, IL8, CCL2, CCL5, known to be involved in crosstalk between CAF and cancer cells, was not statistically different between CAF-S1 and CAF-S4 (Supplementary Fig. 6i, j). In conclusion, CAF-S1 fibroblasts promote BC cell migration and EMT initiation in cancer cells in a CXCL12- and TGFβ-dependent manner. In addition, CAF-S4 fibroblasts stimulate cancer cell invasion and motility in 3D by increasing contractility through the NOTCH signaling pathway.

### CAF-S4-enriched LNs are associated with poor patient survival

**Fig. 8:**
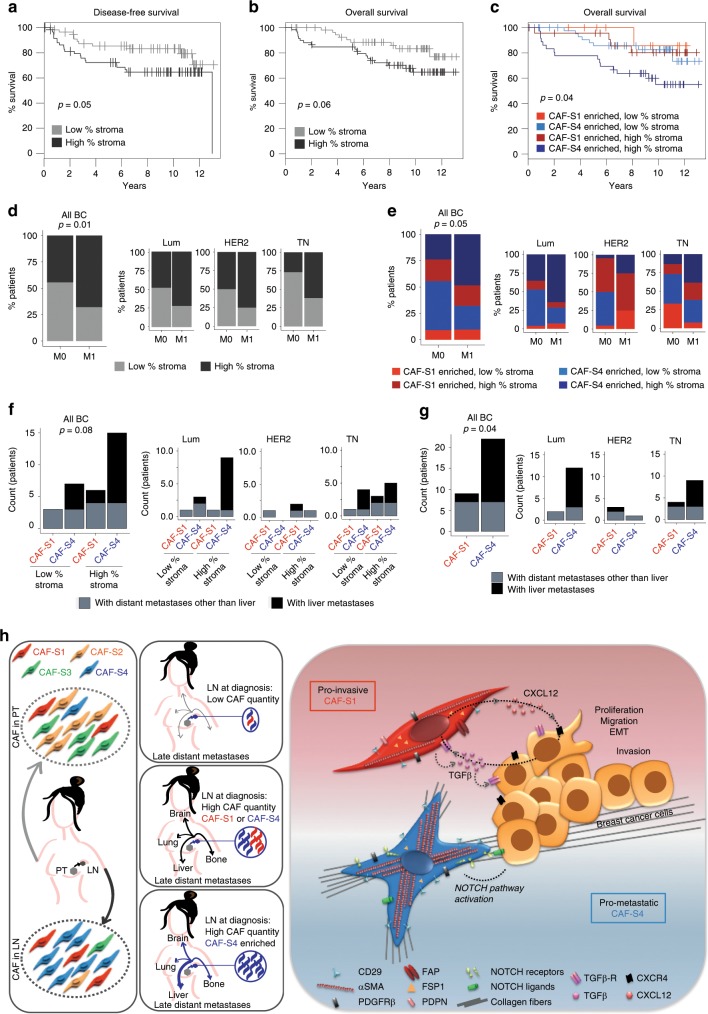
CAF subset content in LNs is a prognostic marker. **a** Kaplan–Meier curves showing patient disease-free survival according to percentage (%) of stroma relative to epithelium in LN sections (*n* = 119). Patient subgroups defined by median. **b** Same as in **a** for overall survival. **c** Overall survival according to both LN stromal quantity (as in **a**) and CAF-subset enrichment, as defined in Fig. 2 (*n* = 119). **a**–**c** *p* Values from log rank test. **d** Left, Distribution of patients with low- or high-LN stromal quantity (as in **a**) without (M0, *n* = 88) or with (M1, *n* = 31) metastases. Right, Same as in left per BC subtypes (65 Lum; 26 HER2; 28 TN). **e** Same as in **d** according to LN stromal quantity and CAF-subset enrichment (same groups as in **c**). **f**, **g** Left, Number of patients with metastases in any distant site except liver (gray) or with at least one metastasis in liver (black), according to CAF content in LNs at diagnosis (*n* = 31) either considering both CAF quantity and CAF-subset enrichments (same groups as in **c**) (**f**) or considering CAF-S1 and CAF-S4 enrichments (**g**). **d**–**g** *p* Values from Fisher’s exact test. **h** Model: Left: four CAF subsets (CAF-S1 to -S4) are detected in metastatic axillary LN, as in PT. Two myofibroblast subsets (CAF-S1, red and CAF-S4, blue) are highly abundant in invaded LNs and correlated with tumor cell invasion. Middle: While patients with low-CAF content in LNs at diagnosis have less risk to develop late distant metastasis, those presenting high CAF-S1/S-4 quantity are prone to develop distant metastases, in particular in liver if LNs are CAF-S4-enriched. Right: Both CAF-S1 and CAF-S4 display pro-invasive properties through distinct mechanisms. CAF-S1 promote cancer cell proliferation, attraction and EMT initiation through CXCL12-CXCR4 and TGFβ axes. Highly contractile and matrix remodeler CAF-S4 induce cancer cell invasion in 3D via NOTCH signaling pathway. Thus, our work reveals the clinical interest of defining CAF subsets content in LNs at diagnosis. Source data provided in Source Data file, with R scripts used.

We finally investigate the clinical relevance of our findings. We analyzed the impact of global stroma content and CAF subset enrichment in LN on disease-free survival, i.e. survival without local, regional or distant relapse (Fig. 8a and Table 1). As CAF-S1 are able to initiate EMT, we quantified the percentage of stromal and epithelial compartments in LNs. High content in stroma in LNs was associated with a shorter disease-free survival, with a similar tendency for overall survival (*p* = 0.06 by Log Rank test and *p* = 0.07 by Cox regression model) (Fig. 8a, b and Table 1). Using multivariate Cox regression analysis with additive model, we observed that the stromal content was independent of BC subtypes and LN status at diagnosis, two well-established independent prognostic parameters (Table 1). As invaded LNs are mainly composed of CAF-S1 or CAF-S4 (Fig. 2), these results suggested that both CAF subsets, when present in high quantity in invaded LNs at diagnosis, might be deleterious for patient survival. We found that CAF subsets on their own exhibited a faint but significant prognostic value, in particular when patients were stratified according to their stromal content in LNs (Fig. 8c and Supplementary Table 6). Indeed, among patients who displayed high stromal content in LNs at diagnosis, those enriched in CAF-S4 showed the poorest overall survival, while no difference according to CAF subset was observed in the context of low stromal content (Fig. 8c). Of note, the prognostic value of CAF subset content in LNs is interesting–although faint—as it is not observed in PT^32,33^. Considering that CAF-S1 and CAF-S4 enhance BC cell invasive capacities and that most BC patients decease from metastases, we next looked at distant metastases developed after diagnosis. In line with our results, patients who developed such metastases were mainly patients with CAF-abundant LNs at diagnosis, with a similar repartition in BC subtype (Fig. 8d). The impact on distant metastatic spread was even stronger in CAF-S4- than in CAF-S1-enriched LN patients, effect detected in all BC subtypes (Fig. 8e). Overall, patients who showed LNs with high stromal content and specific enrichment in CAF-S4 were more prone to develop late distant metastases than any other patients, in particular in liver (Fig. 8f, g). Although metastatic sites vary according to BC subtypes with bone as the most common site except in TN BC associated with other niches, such as liver^1,2^, CAF-S4 impact was not associated to a specific BC subtype. Indeed, among all patients displaying late liver metastasis, only one patient (6%) presented HER2 overexpression at diagnosis. Moreover, CAF-S4-enriched metastatic patients were numerous both within Lum and TN BC subtypes (Fig. 8g). We even detected a higher proportion of patients categorized as CAF-S4-enriched with high stromal content in LNs in the Lum BC subtype compared to TN BC patients (60% Lum versus 33% TN). Thus, patients with high-CAF-S4 content in LNs, who tended to metastasize into liver, were not enriched in TN BC subtype in the cohort analyzed. The impact of CAF subset on metastatic spread was thus not related to a bias linked to BC subtype. On the whole, these data highlight the role of CAF-S1 and particularly CAF-S4 subsets in human BC relapse and metastases (see Model Fig. 8h).

**Table 1:** Univariate and multivariate Cox regression analyses for progression-free survival and overall survival in N+ breast cancer patients–119 cases (LN cohort).

		Disease-free survival						Overall survival					
		Univariate			Multivariate			Univariate			Multivariate		
		Hazard ratio	95% CI	*p* Value	Hazard ratio	95% CI	*p* Value	Hazard ratio	95% CI	*p* Value	Hazard ratio	95% CI	*p* Value
LN stromal percentage	Low % (reference)	1	–	–	1	–	–	1	–	–	1	–	–
	High %	2.1	1–4.3	0.05	2.6	1.2–5.9	0.02	2.1	0.9–4.6	0.07	2.8	1.2–6.6	0.02
LN CAF enrichment	CAF-S1 (reference)	1	–	–	1	–	–	1	–	–	1	–	–
	CAF-S4	1.1	0.5–2.3	0.9	1.4	0.6–3.3	0.4	2.1	0.8–5.4	0.1	3.4	1.2–9.8	0.02
LN status at diagnosis	N1 (reference)	1	–	–	1	–	–	1	–	–	1	–	–
	N2	2.1	1–4.8	0.07	2.5	1.1–5.8	0.03	1.9	0.8–4.6	0.1	2.0	0.8–4.9	0.1
	N3	5.3	2.1–13	0.0004	4.6	1.8–12	0.002	4.5	1.7–11.8	0.002	3.3	1.2–8.9	0.02
BC subtype	Lum A (reference)	1	–	–	1	–	–	1	–	–	1	–	–
	Lum B	1.1	0.4–3.2	0.8	0.7	0.2–2.0	0.5	2.6	0.7–9.4	0.1	1.7	0.5–6.3	0.4
	HER2	0.7	0.2–2.4	0.5	0.7	0.2–2.7	0.6	1.4	0.3–6.2	0.7	1.8	0.4–8.7	0.5
	TN	3.0	1.1–8.0	0.03	3.9	1.4–11	0.009	5.3	1.5–19	0.01	7.3	2.0–27	0.003

*CI* confidence interval

## Discussion

Although the role of CAF in metastatic spread is well established, when analyzed as a global population^9,12,14,18,20,43^, we address here the link between CAF subsets and BC cell spread. Axillary metastatic LN exhibit four CAF subsets (CAF-S1 to S4) but are highly enriched in αSMA+ CAF-S1 and CAF-S4 subsets. The abundance of αSMA+ CAF at PT has been associated with poor prognosis in BC^17,44–48^. In agreement with our findings, a recent study reported that the global stromal content in metastatic LN provides a prognostic stratification of BC patients^49^. We go a step further by differentiating CAF-S1 and CAF-S4 and by showing that the CAF-S1/CAF-S4 status in LNs exhibits a prognostic value, while this is not the case when considering CAF subset enrichment in PT^9,12,14,18,20,43^. This underlines the relevance of our study in BC pathology and indicates that assessing the content in CAF-S1 and CAF-S4 subsets in LNs might be helpful for clinical diagnosis.

Both CAF-S1 and CAF-S4 subsets exhibit pro-invasive properties yet with distinct modes of action. CAF-S1 enhance tumor cell migration and EMT initiation, while CAF-S4 favor cancer cell invasion and motility in 3D. Although the impact of CAF in metastatic spread has been previously demonstrated^50–53^, we show here the complementary role of two distinct CAF subpopulations in metastases. We identify a crosstalk between CAF-S1 and cancer cells involving both CXCL12 and TGFβ, consistent with data showing that CAFs secrete CXCL12 and promote cancer cell migration^16,17,54–58^. In addition, we highlight the role of TGFβ, a well-known EMT inducer^4,59,60^ and an important player in fibroblast activation^50,61–63^. Both CXCL12 and TGFβ expression are found in BC metastatic sites, in particular lung and bones, supporting they are particularly relevant in BC. In contrast to CAF-S1, CAF-S4 are highly contractile and exert a pro-invasive effect on cancer cells. Although mechanisms linking CAF contractility to cancer cell invasion have been described^22,25,64,65^, we unveil here the NOTCH pathway as a key player. NOTCH pathway has been studied in cancer with a strong focus on cancer cells^66–68^. Our data show that NOTCH exerts a key role in CAF-S4 contractility and its pro-invasive action in BC. Thus, we report specific CAF-S1 and CAF-S4-mediated processes and mechanisms that promote metastatic spread in BC.

The four CAF subsets we identified in LN mirror CAF subpopulations in PTs^32,33^. This is consistent with studies showing that LN stroma mimics PT microenvironment^28,30^. But, our results provide new insights. Indeed, if the four CAF subsets are detected in both PT and LN, their abundance strikingly differs. Compared to PT, LNs are almost exclusively enriched in activated CAF-S1 and CAF-S4. The transcriptomic signatures of CAF subsets from LN validated molecular similarities between CAF-S1 (or CAF-S4) from PT and LN. If few studies addressed CAF transcriptomic profiles in both PTs and LNs from BC patients^37,69^, a short gene list identified in ref. ^37^ allows to discriminate LN from PT bulk samples. Interestingly, most of these genes are highly detected in CAF-S4, consistent with their abundance in metastatic LNs. Although CAF origin remains an open question, CAF-S1 and CAF-S4 transcriptomic profiles generated from PT and LN indicate that subset identity is similar between the two tissues. A recent study identified nine stromal populations by single-cell RNAseq in mouse LN, including the established FRCs, FDC and perivascular cells^70^. Although these cells were identified in non-cancerous mouse lymphoid tissues, CAF-S1 are close to CD34+ stromal cells and CAF-S4 to perivascular cells. Our data on CAF-S4 are consistent with previous studies showing the existence of pericyte-like CAF and their potential pericytes-derived origin^32,38–41^. Thus, CAF subsets in invaded LNs could derive from different resident mesenchymal cells. In conclusion, by combining the study of BC patient samples and performing functional assays on human CAF subset cultures, we demonstrate that CAF subsets exhibit distinct functions in metastatic spread. Considering the deleterious effect of metastases on BC patients’ survival, our data might strengthen the interest in assessing CAF subsets content in LN for clinical diagnosis and in using anti-TGFβ and/or anti-NOTCH therapies in BC.

## Methods

### Cohorts of BC patients

The projects developed here are based on surgical residues, available after histopathological analyses, and not required for diagnosis. There is no interference with clinical practice. Analysis of PT and metastatic LN samples was performed according to the relevant national law on the protection of people taking part in biomedical research. All patients included in our study were informed by their referring oncologist that their biological samples could be used for research purposes and they gave their verbal informed consent. In case of patient refusal, that could be either orally expressed or written, residual tumor samples were not included in our study. Human experimental procedures were approved by the Institutional Review Board and Ethics committee (CRI) of the Institut Curie Hospital group (approval 12 February 2014) and CNIL (Commission Nationale de l’informatique et des Libertés) (No. approval: 1674356 delivered 30 March 2013). HER2-amplified carcinomas have been defined according to ERBB2 immunostaining using ASCO’s guideline. Luminal (Lum) tumors were defined by positive immunostaining for ER (estrogen receptor) and/or PR (progesterone receptor). The cut-off used to define hormone receptor positivity was 10% of stained cells. Ki67 (proliferation) score further distinguishes Lum A from Lum B tumors (below 15%: Lum A, above: Lum B). TN immunophenotype was defined as follows: ER^−^PR^−^ ERBB2^−^ with the expression of at least one of the following markers: KRT5/6^+^ or EGF-R^+^.

BC PTs and metastatic LNs from prospective cohorts were collected as part of routine standard of care and included in this study after evaluation by a pathologist. CAF subsets were analyzed from fresh PT and LN samples by FACS (16 PTs and 20 LN), RNA sequencing (5 PTs and their corresponding LN), and cultured in vitro (14 PT and 8 LN). Prospective cohorts mainly include Lum subtypes.

PT (up to 75) and LN samples (up to 124) from retrospective cohorts of BC patients suffering from invasive BC cancers with at least one metastatic LN at diagnosis, have been collected as part of routine standard of care and analyzed by IHC. Retrospective cohorts include Lum, HER2 and TN subtypes. IHC were performed on residual surgery samples prior to any treatment (i.e. prior to radiation, hormonal or chemo-therapy). Overall survival and disease-free survival have been defined as followed: overall survival is defined as the period of time going from diagnosis to death of the patient; disease-free survival is the period of survival without local, regional or distant relapse, meaning the period of time going from diagnosis to the first event occurring among local, regional or distant relapse.

Clinical features of prospective and retrospective cohorts are listed in Tables S1 and S2.

### Flow cytometry on BC PT and LN samples

Fresh human BC PT and LN samples were collected directly from the operating room, after surgical specimen’s macroscopic examination and selection of areas of interest by a pathologist. Samples were cut into small pieces (around 1 mm^3^) and digested in CO_2_-independent medium (Gibco #18045-054) supplemented with 150 μg/ml liberase (Roche #05401020001) and DNase I (Roche #11284932001) during 40 min at 37 °C with shaking (180 rpm). Cells were then filtrated through a 40-μm cell strainer (Fisher Scientific #223635447) and resuspended in PBS+ solution (PBS, Gibco #14190; EDTA 2 mM, Gibco #15575; Human Serum 1%, BioWest #S4190-100) at a final concentration between 5 × 10^5^ and 10^6^ cells in 50 μl.

Cells in suspension were then stained with an antibody mix containing anti-EpCAM^−^BV605 (1:50; BioLegend, # 324224), anti-CD31-PECy7 (1:100, BioLegend, #303118), anti-CD45−APC-Cy7 (1:20, BD Biosciences, #BD-557833), anti-CD235a-PerCP/Cy5.5 (1:50, Biolegend, #349109), anti-CD29-Alexa Fluor 700 (1:100, BioLegend, #303020), anti-FAP-APC (primary antibody, 1:100, R&D Systems, #MAB3715), anti-PDGFRβ-PE (1:40, BioLegend, #323606) and anti-PDPN-Alexa Fluor 488 (1:50, BioLegend, #337006) for surface staining and anti-SMA-Alexa Fluor 594 (1:25, R&D Systems, #IC1420T-025) for intracellular staining. All antibodies except FAP were purchased already conjugated with fluorescent dyes. Anti-FAP antibody was conjugated with fluorescent dye Zenon APC Mouse IgG1 labeling kit (1:100, Thermo Fisher Scientific, #Z25051). Isotype control antibodies for each CAF marker used were: iso-anti-CD29 (1:100, BioLegend, #400144), iso-anti-FAP (primary antibody, 1:200, R&D Systems, #MAB002), iso-anti-PDGFRβ (1:20, BioLegend, #400114), iso-anti-PDPN (1:125, BioLegend, #400525) and iso-anti-SMA (1:25, R&D Systems, #IC003T). For intracellular staining, cells were incubated with violet LIVE/DEAD dye (1:1000, Thermo Fisher Scientific, #L34955) for 20 min at room temperature (RT) in PBS (Gibco, #14190) to exclude dead cells, and then fixed in 4% paraformaldehyde (PFA, Electron Microscopy Sciences, #15710) overnight at 4 °C. After a rapid washing step in PBS + solution, cells were incubated for 45 min at RT with the antibody mix in PBS + supplemented with 0.1% saponin (Sigma-Aldrich, #S7900). For surface staining, cell suspensions were stained immediately after dissociation of BC PT and LN samples during 15 min at RT with the antibody mix in PBS+ solution. 2.5 μg/ml DAPI (Thermo Fisher scientific, #D1306) was added just before flow cytometry analysis. For primary CAF-S1 and CAF-S4 culture characterization, cells at 80% confluency were collected after trypsin treatment, resuspended in PBS + solution at a final concentration of 5 × 10^5^ cells in 50 μl and intracellularly stained with the above-described protocol.

In both conditions (surface and intracellular staining), signals were acquired on the LSRFortessa^TM^ analyzer (BD biosciences) for flow cytometry analysis. At least 5 × 10^5^ events were recorded. Compensations were performed using single staining on anti-mouse IgG and negative control beads (BD biosciences, #552843) for each antibody except anti-PDPN, on ArC reactive beads (Molecular probes #A10346) for live/dead staining and on AbC Total Antibody Compensation Beads (Thermo Fisher Scientific, #A10513) for anti-PDPN.

Data analysis was performed using FlowJo version X 10.0.7r2. Cells were first gated based on forward (FSC-A) and side (SSC-A) scatters (measuring cell size and granularity, respectively) to exclude debris. Dead cells were excluded based on their positive staining for Live/Dead (fixed conditions) or DAPI (surface staining). Single cells were next selected based on SSC-A versus SSC-W parameters. Gating included EPCAM^−^, CD45^−^, CD31^−^, CD235a^−^ cells, to remove epithelial (EPCAM^+^), hematopoietic (CD45^+^), endothelial (CD31^+^) and red blood cells (CD235a^+^). Cells from the negative fraction were next examined using the 5 CAF markers FAP, CD29, αSMA, PDGFRβ and PDPN.

FlowSom algorithm was performed using R packages FlowSOM (1.12.0) and flowCore (1.462), according to the methodology described in ref. ^35^. Self-Organizing Map was performed on all EPCAM^−^, CD45^−^, CD31^−^, CD235a^−^ cells from 16 PTs and from 20 LNs to generate unified trees based on the expression of the five CAF markers. CAF subset populations were manually annotated.

### IHC analyses

BC PT and LN cases were selected by Pathologists (see Supplementary Table 2) and serial sections of paraffin-embedded tissues (PT: Tissue-Micro-Arrays (TMA) using two cores (1 mm diameter, selected as representative from whole-tumor tissue sections by Pathologists) of tumor per case and cut in slices of 3 µm; LN: whole sections of 3-μm thickness) were stained on the Lab Vision Autostainer (Thermo Fisher Scientific). Dewaxing and antigen retrieval on slides prior to immunohistochemical staining were performed with EnVision FLEX Target Retrieval Solution (high- or low-pH, as required—see below—Dako, #K800421 or #K800521) on the Lab Vision PT Module (Thermo Fisher Scientific). Antigen detection was done using the EnVision FLEX/HRP (Dako, #K8006) for CD29, αSMA and PDGFRβ or the streptavidin-peroxidase protocol (Vectastain ABC kit; Vector Labs #PK-6101/6102/6104) plus detection with 3,3′-diaminobenzidine for 5 min (DAB, Dako, #K3468) for FSP1, EPCAM (on LN) and FAP. The following antibodies and respective conditions were used: anti-FAP (pH = 6, 1 h at 1:200, Vitatex #MABS1001), anti-CD29 (pH = 6, 1 h at 1:100, Abcam #ab3167), anti-FSP1 (pH = 6, 1 h at 1:250, Abcam #ab27957), anti-αSMA (pH = 6, 30 min at 1:200, Dako #M0851), anti- PDGFRβ (pH = 9, 1 h at 1:100, Abcam #ab32570), anti-EPCAM (pH = 6, 1 h at 1:200, Dako #M0804), anti-E-cadherin (pH = 9, 1 h at 1:100, Invitrogen #180223). Counterstaining was performed with Mayer hematoxylin freshly prepared (Dako, #S3309). Tissue sections were then submitted to serial gradients of xylen and mounted with coverslip in an automatic device (Sakura, Tissue-Tek DRS).

In each sample, CAF marker staining was evaluated as a histological score (H-score), defined by staining intensity (ranging from 0 to 4) multiplied by the percentage of stained fibroblasts. For LNs, only fibroblasts in metastatic zones—assessed by EPCAM staining—were evaluated. For each PT and LN sample, CAF enrichment was established applying an algorithm developed by the team, which takes as input CAF markers H-scores, as described in refs. ^32,33^. In brief, the thresholds were first defined in a learning dataset on the distribution (1st quartile, median and 3rd quartile) of each marker using FACS data. Thresholds were then transposed to IHC data. For each section, the percentages of stroma and epithelial compartments were evaluated by combining three distinct methods: evaluation on morphological criteria by a referent pathologist; quantification with an automated approach using the QuantCenter image analysis platform (3DHistech) based on morphology assessment and EPCAM staining performed by an independent researcher; EPCAM and E-cadherin staining of epithelial cells. All quantifications gave very consistent results whatever the person (pathologist versus researcher), the method (manually versus automated) or the staining (EPCAM/E-cadherin), thereby arguing that evaluation of stromal and epithelial content was accurate. In each section, percentage of stroma was evaluated as the fibroblastic area multiplied by 100 divided by the total sample area.

### Design of a decision tree for CAF subset prediction

CAF identity was determined by using an algorithm developed by the team (shown in Fig. 2d), which takes as input histological scores of CAF markers^32,33^. The thresholds were first defined, in a learning dataset, on the distribution (1st quartile, median and 3rd quartile) of each marker using FACS data. Thresholds were then transposed to IHC data.

### CAF subsets and epithelial cells at cellular level in situ in invaded LNs

IHC staining from consecutive sections were scanned on Philips Ultra Fast Scanner; 5× images of CAF markers including FAP, CD29, FSP1 and SMA (markers used in the decision tree algorithm, see also Fig. 2d) as well as EPCAM staining from the same areas in representative invaded LNs were further analyzed. Images were aligned using elastic transformation from Fiji software plugin (bUnwarpJ). This plugin uses landmarks manually defined on hematoxylin & eosin staining of the sections to compute the optimal correlation between images and alignment at cellular level. Images were divided into tiles of 225 µm^2^ to mimic the approximate size of one fibroblast and each tile was annotated according to the position in the section. Aligned and annotated images of the CAF markers were then submitted to color deconvolution and the intensity of each DAB staining was measured by densitometry analysis using ImageJ software. Each tile was classified into a specific CAF subset using the algorithm developed by the team (see ‘Design of a decision tree for CAF subset prediction' and Fig. 2d), which takes as input DAB intensities of CAF markers measured within each tile. Epithelial tumor cells were detected based on EPCAM staining to better visualize the stromal compartment and each tile was colored according to the classification into CAF-S1 to CAF-S4, with CAF-S1 red, CAF-S2 orange, CAF-S3 green and CAF-S4 blue.

### Gene expression profiling by RNAseq

CAF-S1, CAF-S4 and EPCAM^+^ cells (from PTs and LNs of five patients, *n* = 28 samples in total, see Supplementary Table 1) were sorted after surface staining (see ‘Flow Cytometry on BC PT and LN samples') on FACSARIA (BD biosciences), directly into RNase-free tubes (Thermo Fisher Scientific #AM12450). At least 100 cells were collected per cell population. Total RNA was extracted with Single Cell RNA Purification kit (Norgen Biotek #51800) following manufacturer’s instructions. RNA integrity and quality were checked with Agilent RNA 6000 Pico Kit (Agilent, #5067-1513) and apparatus. cDNA synthesis and amplification were synthetized using SMART-Seq v4 Ultra Low Input RNA Kit for Sequencing (Clontech #634892). cDNA quality was checked on Agilent 2100 Bioanalyzer using Agilent High Sensitivity DNA kit (Agilent #5067-4626) and quantified using the Qubit dsDNA HS Assay kit (Life Technologies, #Q32854). cDNA libraries were prepared using Nextera XT preparation kit (Illumina #FC-131-10). Samples were sequenced on HiSeq 2500 (Illumina) rapid run flow cells with an average sequencing depth of 34.5 million paired-end reads. Read length was 100 bp. Reads were mapped on reference human genome (hg19/GRCh37 from UCSC genome release) using Tophat_2.0.6 algorithm with the following parameters: global alignment, no mismatch in seed alignment (of size 22), three mismatches in read length. Quality control was performed using FastQC software and duplicates were removed using Samtools rmdup. Gene expression quantification was performed using HTSeq-count and featureCounts (implemented in Bioconductor R package Rsubread (1.18.0)). Only genes with at least one read in 5% of all samples were kept for further analyses. Normalization was performed with DESeq2 R package (1.8.2) method. Analysis strategy includes unsupervised analyses such as PCAs and HC, as well as paired differential expression analyses (done with DESeq2 bioconductor package). Biological interpretation of the identified genes was done by computational functional analyses on Metascape (http://metascape.org), which is based on several bioinformatics resources (Gene Ontology, KEGG, Reactome). RNAseq data have been deposited on EGA website (EGA number: EGAS00001003238).

### Cell culture

The human BC cell lines MCF-7(-GFP), T47D(-GFP) and MDA-MB-231-GFP were propagated in DMEM (GE, #SH30243.01) supplemented with 10% fetal bovine serum (FBS) (Biosera, #1003/500), penicillin (100 U/ml) and streptomycin (100 μg/ml) (Gibco #15140122). Each cell line identity was verified by Short Tandem Repeat (STR) DNA profiling (Promega #B9510).

CAF-S1 and CAF-S4 primary cultures were established from PT and LN human samples. All along the duration of the project, we isolated 22 CAF-S1 and 22 CAF-S4 paired primary cell lines (referred to as CAF pairs because isolated from the same patient) using FACS-sorted cells full-filling marker criteria shown in Fig. 1. Among them, 64% were from PTs and 36% from LNs. In agreement with the strong transcriptomic analogy of CAF subsets from PT or LN (shown Fig. 3), we did not observe any difference in functional assays when we performed experiments with CAF-S1 or CAF-S4 isolated either from PT or from LN. Thus, we observed no effect of the site of isolation (PT or LN) on CAF-S1/CAF-S4 properties. Transcriptomic signatures and in vitro properties were thus equivalent whatever the site of CAF initial localization (PT or LN). Although CAF-S1 were quite easily expanded in vitro, CAF-S4 were difficult to maintain. As they exhibit a pericyte-like signature, we adapted the culture conditions to expand and maintain both CAF-S1 and CAF-S4 in similar and comparable conditions. BC samples were processed and sorted after surface staining as described above (see ‘Flow Cytometry on BC PT and LN samples') by cell sorting and gating strategy using FACS shown in Fig. 1. Purified CAF-S1 and CAF-S4 were then plated in 96-well plates and expanded in a pericyte medium (Sciencell, #1201) in a humidified, 1.5% O_2_ and 5% CO_2_ incubator. Doubling time was assessed during this expansion phase for six CAF pairs over five passages. All cultures were systematically validated by qRT-PCR between passages 3 and 6 using genes upregulated in either CAF-S1 or CAF-S4 (including *SFRP2, HMCN1, TINAGL1, PDE3A*). Other types of validation were done, such as RNAseq on four CAF-S1 and CAF-S4 pairs and FACS analyses (see ‘Flow Cytometry on BC PT and LN samples') to assess CAF marker protein levels (as shown in Supplementary Fig. 3). For RNAseq experiment, cells at 80% confluency were collected after trypsin treatment, total RNA was extracted (Qiagen #217004) and DNAse-treated (Life technologies #EN0525). mRNAs were retro-transcribed, amplified and cDNA libraries were obtained with TruSeq Stranded mRNA library prep (Illumina #RS-122-2101). Samples were then sequenced and analyzed as PT and LN fresh CAF samples (see ‘Gene expression profiling by RNAseq'). To validate their identity, PCA and HC were performed using CAF-S1 and CAF-S4 top-500 gene signatures. These signatures were defined as the 500 genes upregulated and the 500 genes downregulated between PT + LN CAF-S1 (*n* = 10) and CAF-S4 (*n* = 10) fresh paired samples (paired differential analysis by DESeq2). Twenty-two different CAF-S1 and CAF-S4 paired primary cell lines were isolated from BC PTs (*n* = 14) and LNs (*n* = 8) (see Supplementary Table 1 for clinical information) were used for functional experiments. All experiments were performed in DMEM with 10% FBS at 1.5% O_2_ unless otherwise specified. For BC cells and primary CAF cell lines, the absence of mycoplasma contamination has been tested and confirmed.

### Silencing of CXCL12, CXCR4 and transient transfection

For the short interfering RNA (siRNA) experiment, CAF-S1, CAF-S4 or breast cancer cells were transfected with 20 nM siRNA. Control was non-targeting siRNA (siCTR, AllStars negative control, #1027281), CXCL12 silencing was performed with a siRNA pool targeting both α- and β-CXCL12 isoforms (Dharmacon, #L-007873-00) and CXCR4 silencing was achieved with a pool of four specific siRNA (Dharmacon, #L-005139-00). Transfections were performed with DharmaFECT 1 (Dharmacon, #T-2001-02) transfection reagent according to manufacturer’s instructions.

### qRT-PCR from CAF subsets and BC cell lines

For gene expression analysis, total RNA isolation was performed using miRNEasy kit (Qiagen, #217004) according to manufacturer’s instructions. RNA concentrations were determined using a NanoDrop apparatus (NanoDrop Technologies, Inc.). For each sample, 1 μg of total RNA was reverse transcribed using an iScript Reverse Transcription Kit (Bio-Rad #1708840). qRT-PCR was performed using Power SYBR Green PCR Master Mix (Applied Biosystems, #4367659) on a CFX96 or CFX384 Touch Real-Time PCR Detection System (Bio-Rad) with primers at 300 nM final concentration. Primers (forward and reverse) used for quantitative (q)RT–PCR amplification were: CXCL12: 5′–CTACAGATGCCCATGCCGAT–3′ 5′–CAGCCGGGCTACAATCTGAA–3′, CXCR4: 5′–TGGCCTTATCCTGCCTGGTATTGT–3′ 5′–AGGAGTCGATGCTGATCCCAATGT–3′. Silencing validation was performed from at least four independent experiments.

### Transwell assay

Twelve-well cell culture inserts (Corning, 8 μm pore size, #353182) were used for migration assays, and migration was assessed 24 h after plating.

For CAF migration, after 24 h serum deprivation (0.5% FBS), 4 × 10^4^ cells were plated in the upper side of the Transwell device in 0.5% FBS medium. Six CAF-S1 and CAF-S4 pairs were tested in four independent experiments, in duplicates.

For CAF chemo-attraction experiments, 10^5^ CAF (CAF-S1 and CAF-S4) were plated in duplicates in 12-well plates. After CAF attachment (6 h), cells were gently washed with PBS and medium changed to 0.5% FBS. In parallel, MCF7 and T47D that had been serum deprived (0.5% FBS) for 24 h were collected and 10^5^ cells were plated in the upper side of the Transwell device in 0.5% FBS medium, on top of 0.5% FBS medium ± CAF. Representative images of Transwell membrane underside shown in Fig. 4 are obtained 24 h after CAF-S1/CAF-S4 seeding in upper part. At least eight independent experiments including at least four different CAF-S1 and CAF-S4 pairs were performed with each BC cell line, each condition in duplicates.

To assess CXCL12 involvement in CAF-S1 attraction of BC cells, 10^5^ CAF-S1 were plated as described above, after 48 h of transient silencing (see ‘Silencing of CXCL12, CXCR4 and transient transfection'). BC cell number was increased to 3 × 10^5^ cells per insert and plated in triplicates. Three independent experiments including at least two different CAF-S1 cell lines per BC cell type were performed.

At the end of the experiment, the remaining cells in the upper side of the Transwell device were gently removed with cotton swabs moistened with PBS. Migrating cells at the bottom side of the Transwell device were fixed and stained with crystal violet for 30 min and then counted in seven different representative fields per insert (X5 objective, Zeiss Axioplan microscope, AxioCamERc 5s, manual counting on Fiji software) and averaged per condition. When we adjusted the migration to proliferation rate, we divided the migration capacity by the fold change in tumor cell proliferation in presence/absence of CM from CAF subsets.

### Cell exclusion zone assay

For cell exclusion zone assay, Ibidi culture inserts (#80209) were added to 12-well plates (one insert per well) and 10^4^ CAF-S1 or 10^4^ CAF-S4 were plated per chamber of each insert, in duplicates. After cell attachment, inserts were gently removed, delicately washed with PBS and 2 ml of medium was added to each well. Time-lapse videos were then immediately acquired with an inverted motorized Leica video-microscopes, equipped with motorized stage, 37 °C incubator and CO_2_ controller (Leica DMi8 with a Retiga R6 camera and illumination by Lumencor SOLA SE 365). The automated imaging system was controlled by the software Metamorph (Universal Imaging). Transmission images were acquired every 15 min until 50 h and one position per insert (hence two per condition) was taken with a 5× objective as it encompassed 80% of the cell-free zone. For data analyses, Fiji software was used. In each position, manual tracking of 40 CAF-S1 and CAF-S4 cells per condition positioned on the free edges were tracked over 24 h, taking into consideration the first image of each hour. Velocity (distance of migration divided by the interval time) and direction (absolute value of sin(*α*), where *α* is the angle between the velocity and the initial cell-free zone border – absolute because cells were taken from both sides of the cell-free zone) were calculated for each successive pair of time points for each cell. Means per cell over the global tracking are shown. Persistence of cell trajectory, defined as the ratio *d* */* *l* where *l* is the contour length of the total trajectory and *d* the direct end-to-end distance, was also calculated per cell over the same 24 h. Two different CAF-S1 and CAF-S4 pairs were tested in two independent experiments.

### CAF spheroids and invasion assay

For spheroid invasion assays, CAFs were trypsinized and resuspended at a concentration of 4 × 10^5^ cells/ml. Atotal of 20 μl drops were pipetted down on a 10-cm dish lid. Lids were then gently put back on the plates filled with medium to humidify the system. After 4 days, CAF spheroids—formed in the hanging drops—were carefully transferred into 2.5 mg/ml collagen solution drops (Corning, #354249) in the center of 6-well plates. At least six different spheroids per CAF were embedded in collagen and further analyzed. After polymerization, 0.5% FBS medium was added to the wells. Spheroids were imaged 3 days after inclusion on a Nikon Eclipse TE300 microscope with a 4× objective equipped with a Nikon DS-Fi1 camera and invasion was assessed with Fiji software as an invasion index defined as the total area covered by CAFs (encompassing single cells escaped from the spheroid and the spheroid) divided by the area of the spheroid core center. Three CAF-S1 and CAF-S4 pairs were tested in three independent experiments. For time-lapse imaging, time-lapse videos were immediately acquired after collagen polymerization with an inverted motorized Leica video-microscopes, equipped with motorized stage, 37 °C incubator and CO_2_ controller (Leica DMi8 with a Retiga R6 camera and illumination by Lumencor SOLA SE 365). The automated imaging system was controlled by the software Metamorph (Universal Imaging). Transmission images were acquired every 15 min until 42 h with a 5× objective to encompass the full spheroid. Videos were mounted with eight frames per second.

### Collagen contraction assay

To assess force-mediated collagen contraction, 3 × 10^4^ CAFs were embedded in 100 μl of a 2.5-mg/ml collagen solution (Corning, #354249) and seeded in triplicates in 96-well plates. After polymerization, culture medium was added on top of the gels and in the surrounding empty wells to limit evaporation. To assess DAPT (Sigma, #D5942), BLEBBISTATIN (Sigma, #B0560) and Y27632 (STEMCELL Technologies, #72302) impact on CAF contractility, 10 μM of each drug was added to the medium, DMSO as control. Gel contraction was assessed 4 days after plating: plates were scanned (EPSON Perfection V700 Photo scanner) and quantification was performed on Fiji software. Percentage of contraction was calculated using the formula:

$100 \times \frac{{{\mathrm{{Area}}}_{{\mathrm{{well}}}} - {\mathrm{{Mean}}}\left( {{\mathrm{{Area}}}_{{\mathrm{{Gel}}}}} \right)_{3{\mathrm{{replicates}}}}}}{{{\mathrm{{Area}}}_{{\mathrm{{well}}}}}}$. Six CAF-S1 and CAF-S4 pairs were tested in four independent experiments. For DAPT experiments, three independent experiments were performed, including four different CAF-S4 cell lines.

### Traction force microscopy

For TFM experiments, 2.5 × 10^4^ CAFs were seeded on collagen pre-coated 25 kPa hydrogels containing fluorescent beads (Matrigen, #ST-0.2YG-SW6G-COL-25 EA). These commercial hydrogels contain fluorescent beads of 0.2 µm diameter at a density of 0.2 ± 0.04 beads/µm^2^. Three CAF-S1 and CAF-S4 pairs were tested. Cells were plated at low density (~25 cells/mm^2^) to ensure that traction forces were measured on single cells. When DAPT (Sigma, #D5942) treatment was evaluated, 10 μM was added to the medium, DMSO as control. Gels (transmission and GFP channels) were imaged on an inverted Leica video-microscope (Leica DMi8 with a Retiga R6 camera and illumination by Lumencor SOLA SE 365) 24 h post-plating with 20× objective, the large size of CAF cells with elongated protrusions precluding the use of higher magnification. Images of the relaxed gels were acquired after removing the cells with trypsin. Beads images (with and without the cells) were first aligned using the ImageJ plugin “Align slices in stack”.

Bead displacements (with respect to the resting state after trypsination) were measured and converted to cell traction stresses using the open source Fourier transform traction cytometry Fiji plugins (available at https://sites.google.com/site/qingzongtseng/tfm or org.insb.bib.cnrs.fr/10.1016/bs.mcb.2014.10.008)

Images were processed with ImageJ software. Further analyses were performed with Matlab (MathWorks, Natick, MA). To quantify cell contractility, we measured the strain energy (SE) which is the total energy transferred from the cell to the elastic substrate, the strain energy density which is the energy per unit area (SE divided by the cell area) and the mean traction stress amplitude which corresponds to the average stress per cell. ${\mathrm{{SE}}} = \frac{1}{2}\int \nolimits_{{\mathrm{{Cell}}}} {\vec T(\vec r).\vec u(\vec r)d^2\vec r},$

where $\vec T$ is the traction stress field and $\vec u()$ is the displacement field.

### Collagen evaluation by second harmonic generation

CAFs were seeded in home-made holes in 30 mm^2^ tissue culture plates as described in ref. ^23^: three holes of 3–4 mm diameter were drilled in a plate and widened around the edges using a scalpel. The bottom of the dish was covered with epoxy (Loctite), and 20 × 20 mm square coverslips were glued to the dish overnight at RT. The day before the experiment, dishes were silanized with 3-aminopropyl-trimethoxysilane (Sigma-Aldrich) and extensively washed with water treated for 30 min with 0.5% glutaraldehyde followed by a final wash, to avoid collagen detachment from the plastic holes caused by CAF contractility. A total of 3000 CAF—2 CAF-S1 and CAF-S4 pairs were tested—were embedded in 15 μl of a 2.5-mg/ml collagen solution (Corning, #354249) and seeded in duplicates in the afore-mentioned holes. After polymerization, medium was added to the plates. Cells were left 3 days in this system, then fixed for 30 min in 4% PFA, permeabilized with 0.1% Triton X-100 in PBS for 30 min and F-actin was stained using phalloidin (Thermo Fisher Scientific, #R415, 1:200 O/N in PBS at 4 °C). Images (of at least 10 cells per CAF over two wells) were acquired with an inverted AOBS two-photon laser-scanning confocal microscope (SP8; Leica) coupled with a femtosecond laser (Chameleon Vision II; Coherent Inc.) using a 25×/1.0 NA water immersion objective. The microscope was equipped with three nondescanned HyD detectors: NDD1 (500–550 nm), NDD2 (≥590 nm) and NDD3 (405 nm). Fluorescence channel was recorded using the excitation wavelength 561 nm. Collagen was visualized by second harmonic generation (SHG) using the excitation wavelength 910 nm. 3D stacks were acquired at a step size of 1 µm intervals to encompass one whole cell. Images were further analyzed with Fiji software. All collagen images were processed in the same way: light was adjusted (min = 0 and max = 50), thresholded (min = 0, max = 30), converted to mask, smoothed, despeckled and collagen area was measured on all slices of each 3D-stack. Collagen density around each cell was then computed as $d = \frac{{\mathop {\sum }\nolimits_{i = 1}^n \Delta z \times {\mathrm{{surf.coll}}}_{{\mathrm{{slice}}}(i)}}}{{V_{{\mathrm{{stack}}}}}}$, where Δ*z* is the step between two slices in the stack, *n* the number of slices in the stack, *V*_stack_ the volume of the stack and surf.coll_slice(s)_ the area covered by collagen in the slice *i* of the stack (Fiji output).

### Proliferation assays

CAF doubling time was assessed over five passages for six pairs or CAF-S1 and CAF-S4 primary cell lines, CAFs being sub cultured when reaching 90% confluency in the plate (see ‘Cell culture').

For co-culture proliferation assay, 10^5^ CAF (-S1 or -S4) and/or 5 × 10^4^ MCF7 cells (1.5 × 10^5^ CAF (-S1 or -S4) and/or 7.5 × 10^4^ T47D cells) were plated in duplicates in 6-well plates. After cell attachment, cells were washed in PBS and 0.5% medium was added; 24 h or 72 h later, cells were collected (cells in supernatant as well), washed with PBS+ and stained for 15 min at RT with anti-EpCAM-BV605 (BioLegend, #324224) and anti-FAP-APC (primary antibody, R&D Systems, #MAB3715 conjugated with fluorescent dye Zenon APC Mouse IgG1 labeling kit, Thermo Fisher Scientific, #Z25051) to distinguish CAFs from BC cells. After a last washing step, all samples were resuspended in 50 μl of PBS+ containing 2.5 μg/ml DAPI (Thermo Fisher scientific, #D1306) and 1:100 carboxylated beads (Polyscience, #18133). Total samples were acquired on the LSRFortessa^TM^ analyzer (BD biosciences) and precision beads were used to normalize viable BC cell (DAPI^−^ EPCAM^+^ FAP^−^) and CAF (DAPI^−^ EPCAM^−^ FAP^+^) counts. At least six independent experiments including at least three different CAF-S1 and CAF-S4 pairs were performed per BC cell line.

For BC cell viability assessment with CAF CM (three CAF pairs for each three independent experiments and four different CAF-S1 in two independent experiments for CXCL12 silencing), as well as CAF viability assessment with DAPT, BLEBBISTATIN (Sigma, #B0560) or Y27632 (STEMCELL Technologies, #72302) treatment (three independent experiments with two different CAF-S4 each time), we used resazurin assay. Briefly, 10^4^ BC cells were seeded in triplicates in 96-well plates. To evaluate CM-CAF effect, once BC cells attached, cells were gently washed with PBS and 100 μl of CAF-CM were added to each well. Cell viability was assayed 24 h later. To obtain CM-CAF, 10^5^ CAF were plated in 12-well plates in duplicates (24 h post siRNA transfection for CXCL12 silencing). After attachment, cells were gently washed with PBS and 1 ml of 0.5% FBS medium was added per well. After 24 h, supernatants were collected, duplicates were mixed, centrifugated and immediately used for experiments. For CAF viability assessment, 7 × 10^3^ CAFs were seeded in triplicates in 96-well plate wells with the different drugs at 10 μM in the medium (DMSO was used as control). Cell viability was assessed after 24 h, 72 h and 7 days. To do so, 25 μl of resazurin reagent (0.05 mg/ml, Sigma #R7017) was added to each well. Plates were incubated at 37 °C for 4 h and read in a Multi Detection plate reader (Fluostar, BMG Labtech).

### Immunofluorescence

For immunofluorescence experiments, 200,000 BC cells ± 200,000 CAF (24 h post-silencing when CXCL12 and CXCR4 roles in EMT-triggering was assessed) were seeded on coverslips placed in 6-well plate wells. When TGFβ-R involvement was assessed in EMT-triggering, the TGFβ-RI/II inhibitor LY2109761 (Selleckchem, #S2704) was added at 5 μM in the medium (DMSO was used as control). Forty-eight hours later, cells were fixed in 4% PFA for 15 min, permeabilized with 0.1% SDS in PBS for 10 min, blocked in PBS-Tween 0.1% with 5% BSA (Euromedex, #04-100-812-C) for 30 min and incubated with antibodies diluted in PBS-Tween 0.1% with 5% BSA overnight at 4 °C. Antibodies were anti-Vinculin (1:1000, Sigma, #V9131) or E-cadherin (1:300, Cell Signaling Technology, #3195). Cells were then incubated with Cy3-anti-mouse secondary (1:500, JacksonImmunoResearch, #715-165-150) or Cy3-anti-rabbit secondary (1:500, JacksonImmunoResearch, #711-165-152) in parallel with Alexa Fluor TM 488 phalloidin (1:200, Invitrogen, #A12379) for 30 min at RT in PBS-Tween 0.1% with 5% BSA. After several washing steps, coverslips were mounted on slides with a drop of Vectashield mounting medium with DAPI (Vector, #H-1200). Slides were then examined using an upright Epifluorescence Microscope with Apotome (Zeiss) with a 40× oil-immersion objective and images (at least five positions per condition) were acquired with identical exposure times and settings using a digital camera (Photometrics CoolSNAP HQ2). At least two independent experiments were performed for each setting and each BC cell line (one CAF pair for CAF-S1 versus CAF-S4 impact in two independent experiments; three CAF-S1 in three independent experiments for CXCL12 involvement; two CAF-S1 in two independent experiments for CXCR4 involvement; at least three CAF-S1 in at least three independent experiments for TGFβ-R involvement).

### Tumor cell density, E-cadherin in BC cells, F-actin and Vinculin in CAF

For tumor cell density and assessment of staining in CAF subsets, quantifications were performed in two steps on FiJi software. (1) Masks were manually drawn on each merged image to either keep tumor cell zones of CAF zones according to the analyses. Masks were then applied to the corresponding single channel (Dapi, E-cadherin, F-actin or Vinculin depending on the analyses) images. (2) For BC cell density, evaluation of cell number was automatically performed by applying a threshold filter (0, 20) on Dapi channel (raw images opened and auto-scaled with Bio-formats plug-in, saved in jpeg format) followed by a mask conversion, segmentation and then particles were analyzed (size 2000–25,000; circularity > 0.2). Cell number was then divided by the tumor zone area. For F-actin or Vinculin signal extraction in CAF, corresponding images were thresholded (a single threshold was set up per experiment on raw images opened and auto-scaled with Bio-formats plug-in, saved in jpeg format: 125 for F-actin, 150–175 for Vinculin), converted to masks, integrated density was retrieved and then divided by CAF zone area. For E-cadherin signal extraction, as CAFs were negative for E-cadherin, step (1) was skipped and step (2) was directly performed. E-cadherin signal area shown in Figs. 5, 7 was defined on ImageJ, and next divided by tumor cell surface. Images were thresholded (single threshold per experiment ranging from 50 to 225 applied on raw images opened and auto-scaled with Bio-formats plug-in, saved in jpeg format), converted to masks, integrated density was retrieved and then divided by tumor zone area.

### Inverted Transwell assay

For inverted Transwell assays, CAF were first labeled with fluorescent dyes (Thermo Fisher Scientific, for CAF-S1: CellTrace Yellow #C34573, for CAF-S4: CellTrace Violet, #C34571) according to manufacturer’s protocol; 2 × 10^4^ cells were then embedded in 100 μl of a 2.5-mg/ml collagen solution (Corning, #354249) and seeded inside 24-well cell culture inserts (Corning, 8 μm pore size, #3422). CAF-free collagen was used as control. After polymerization, inserts were put upside-down on plate lids and 4 × 10^5^ MDA-MD-231-GFP were seeded on the bottom part of the inserts and left to adhere for 2 h. Inserts were then placed in 24-well plates containing 0.5% FBS and complete medium was added inside the insert plates. After 24 h, inserts were gently transferred into new 24-well plates containing 0.5% FBS and further incubated for 6 days. All conditions were done in duplicates. After 7 days of invasion, inserts were gently washed in PBS, fixed in 4% PFA for 15 min, washed again and mounted with Mounting medium (Aqua-Poly/Mount #18606) on glass-bottom dishes (FisherScientific #15159112). Images were then acquired on an inverted Laser Scanning Confocal Microscope with Spectral Detection and Multi-photon Laser (LSM880NLO/Mai Tai Laser—Zeiss/Spectra Physics) using a 25×/0.8 NA oil immersion objective. 3D stacks were acquired at a step size of 10 µm intervals to encompass all invading cells (initial *Z* set up so that membrane pores were in focus and highest *Z* where no MDA-MB-231-GFP cells could be detected anymore). Fluorescence channels were recorded using the excitation wavelengths 405 nm (for CAF-S4), 488 nm (for BC cells) and 561 nm (for CAF-S1). At least four stacks were acquired per insert, *x* and *y* = 566.8 μm; *z* = 200 μm. Images were further analyzed with IMARIS software to retrieve the distance of invasion of all BC cells in each stack, after modeling cells on GFP signals done with the same parameters across stacks (surface detail 2 μm; threshold absolute intensity 3.00; option “enable splitting of touching objects” was selected; seed points diameter 15 μm; filtered on voxels above 350). Each stack was normalized to the number of detected cells in the stack to express frequencies of cells. Maximal distance of invasion was defined as the value of *Z* above which 1% of cells in stack was found. Data were then averaged per condition per experiment. Three independent experiments with two different CAF-S4 cell lines were performed.

For DAPT (Sigma, #D5942) treatment, when CAF-S4 were used, they were labeled and included in collagen as described above. MDA-MB-231-GFP cells were seeded as described above (below CAF-S4-free collagen for control experiments or CAF-S4-embedded collagen). 10 μM DAPT or DMSO was added on both sides of the inserts and media were changed at day 3. All conditions were done in duplicates. Inserts were fixed, acquisition and analysis were performed as described above with the following Imaris parameters: surface detail 2 μm; Threshold absolute intensity 2.00; option “enable splitting of touching objects” was selected; seed points diameter 15 μm; filtered on voxels above 300. 5 independent control experiments were performed and 6 independent experiments including 5 different CAF-S4 cell lines were performed to assess DAPT effect on CAF-S4-induced invasion of BC cells.

### Tumor-on-chip assay

The devices were made of PDMS (polydimethylsiloxane), a biocompatible, gas-permeable and transparent silicone rubber. The devices were micro-fabricated at the Institut *Pierre-Gilles de Gennes pour la Microfluidique* (IPGG, Paris) using standard soft-lithography or advanced micro-milling for the mold fabrication and soft-lithography methods to prepare the chips. The microfluidic device were sterilized in UV-oven (365 nm wavelength) for 30 min, coated with 10 µg/ml human fibronectin (Sigma, #F0895) overnight at 37 °C, 5% CO_2_, rinsed with PBS and then dried for at least 5 h in a sterile room. Cells were embedded in 2.3 mg/ml collagen hydrogels (PureCol, Advanced Matrix, # 5005 diluted in MEM medium (Sigma Aldrich, # M0275) containing 0.28% NaHCO_3_ (pH = 8) to adjust the final pH to 7). MCF7-GFP or T47D-GFP targeted density was 1.2 × 10^6^ cells/ml, CAF density was 3 × 10^5^ cells/ml. When DAPT (Sigma, #D5942) was used, CAF-S4 were pre-treated overnight before inclusion in collagen and a final concentration of 10 μM was added to the medium. We verified that DMSO did not interfere with BC cell velocity in the system. Time-lapse videos were then acquired with an inverted motorized Leica video-microscopes, equipped with motorized stage, 37 °C incubator and CO_2_ controller (Leica DMi8 with a Retiga R6 camera and illumination by Lumencor SOLA SE 365) in a 20% O_2_ environment. The automated imaging system was controlled by the software Metamorph (Universal Imaging). Transmission and fluorescent (GFP) images were acquired every 2 h over 30 h and at least four positions per condition were recorded with a 10× objective. For data analyses, Fiji software was used. In each position, cells were manually tracked over all frames (‘Manual tracking’ plugin, at least 65 cells per condition). Cell velocity (distance of migration divided by the interval time) was calculated for each successive pair of time points. Means per cell over the global tracking are shown. For CAF-S1 versus CAF-S4 experiments, for each BC cell line: three independent experiments were performed, with three different CAF-S1 and CAF-S4 pairs. For DAPT experiments, for each BC cell lines: three independent experiments were performed, including two different CAF-S4 cell lines.

### Cytokine antibody-pair-based assay

Cytokine antibody-pair-based assays were performed using human cytokine array kit (R&D Systems, #ARY005B) according to the manufacturer’s protocol. In brief, 80,000 CAF-S1 or CAF-S4 fibroblasts were cultured alone or in presence of 80,000 BC cells (MCF7 or T47D) in 1 ml of DMEM medium in 12-well plates at 1.5% O_2_ during 48 h. Culture supernatants were next collected (CM) and centrifuged at 13,000 rpm during 10 min for eliminating debris. Array membranes, previously spotted with capture antibodies by the manufacturer, were incubated with 0.5 ml of CM overnight at 4 °C. Membranes were then washed three times with 50 ml of washing buffer at RT, and incubated with a streptavidin–horseradish peroxidase-coupled antibody (1:2000) for 30 min at RT and revealed using Chemi-Reagent Mix. The immunoblot images were captured and visualized using the ChemiDoc Imaging System (Bio-Rad) and intensity of each spot in the captured images was measured using ImageJ software. Two spots per membrane were used to measure the levels of each cytokine. Intensity mean of the two spots—revealing each cytokine level per experiment—was reported to the intensity mean of three internal controls spotted on the membrane and assessed by the following ratio: specific intensity mean / control intensity mean, to take into consideration variation in hybridization efficiency in the different experiments.

### Software

Software used for the above analyses are: R (https://cran.r-project.org, R versions 3.3.1, 3.4.0 and 3.5.0), Fiji (ImageJ v2.0.0-rc-19/1.49m, v2.0.0-rc-65/1.52b), IMARIS (v8.4.1), Matlab.

### Statistical analyses

Data graphical representation and statistical analyses were done using R or GraphPad Prism environment. Barplots represent means ± standard errors of the mean (SEM). Boxplots: box limits indicate the inter-quartile range (IQR) (25th to 75th percentiles), with a center line indicating the median. Whiskers show value ranges up to 1.5 × IQR above the 75th or below the 25th percentiles, with outliers beyond those ranges shown as individual points. The number of independent experiments is specified in each figure legend, with at least three independent experiments, unless otherwise specified. The statistical tests used are in agreement with the data distribution: normality was first checked using the Shapiro–Wilk test and parametric or non-parametric two-tailed tests were applied according to normality. Statistical test types are indicated in figure legends. Survival analyses were carried out with survival R package by Kaplan–Meier curves (*p*-values from Log-rank test) and by univariate and multivariate Cox regressions using additive hazards regression models. Stratification of patients for Kaplan–Meier analyses were performed using median value of the stromal content distribution and CAF-S1 or CAF-S4 enrichments (defined by our algorithm, see ‘IHC analyses'). Differences were considered statistically significant when *p* ≤ 0.05.

### Reporting summary

Further information on research design is available in the Nature Research Reporting Summary linked to this article.

## Supplementary information

Supplementary Information

Supplementary data

Additional Supplementary Files

Supplementary Movie 1

Supplementary Movie 2

Reporting Summary

## Data Availability

RNAseq data from EPCAM^+^, CAF-S1 and CAF-S4 sorted from BC samples, without or with culture, generated in this study are available on European Genome-Phenome Archive platform (https://ega-archive.org) under accession number: EGAS00001003238. A source data file corresponding to all panels of the figures and supplementary figures that support the findings of this study is provided with the paper.

## Code availability

R scripts used to generate panels of the figures and supplementary figures are provided within the data source file of the paper, available at 10.6084/m9.figshare.9924275.
