# Supplementary material for: Cancer-associated fibroblast heterogeneity in axillary lymph nodes drives metastases in breast cancer through complementary mechanisms
Source: Nat Commun. 2020 Jan 21;11:404. doi: 10.1038/s41467-019-14134-w (PMC6972713; doi:10.1038/s41467-019-14134-w)
Supplement: Supplementary file 1 — Supplementary Information [file 41467_2019_14134_MOESM1_ESM.pdf]

SUPPLEMENTARY INFORMATION

Cancer-associated fibroblast heterogeneity in axillary lymph nodes drives metastases in breast cancer through complementary mechanisms

Floriane Pelon[1,2], Brigitte Bourachot[1,2], Yann Kieffer[1,2], Ilaria Magagna[1,2], Fanny Mermet-Meillon[3], Isabelle Bonnet[4], Ana Costa[1,2], Anne-Marie Givel[1,2], Youmna Attieh[5], Jorge Barbazan[5], Claire Bonneau[1,2], Laetitia Fuhrmann[6], Stéphanie Descroix[7], Danijela Vignjevic[5], Pascal Silberzan[4], Maria Carla Parrini[3], Anne Vincent-Salomon[6] and Fatima Mechta-Grigoriou[1,2,*]

[1] Institut Curie, Stress and Cancer Laboratory, Equipe labélisée par la Ligue Nationale contre le Cancer, PSL Research University, 26, rue d'Ulm, F-75005 Paris, France.

[2] Inserm, U830, 26, rue d'Ulm, Paris, F-75005, France.

[3] Analysis of transduction pathway, Institut Curie, Inserm, U830, PSL Research University, 26 rue d'Ulm, Paris, F-75005, France.

[4] Institut Curie, Biology-inspired Physics at MesoScales Laboratory, Equipe labélisée par la Ligue Nationale contre le Cancer, CNRS UMR168, PSL Research University, Sorbonne Université, 26, rue d'Ulm, F-75005 Paris, France.

[5] Institut Curie, Cell migration and invasion, UMR144, PSL Research University, 26, rue d'Ulm, F-75005 Paris, France.

[6] Department of Pathology, Institut Curie Hospital, 26, rue d'Ulm, F-75248 Paris, France.

[7] Institut Curie, Laboratoire Physico Chimie Curie, Institut Pierre-Gilles de Gennes, CNRS UMR168, 75005 Paris, France.

Running title: Dual impact of CAF subsets on breast cancer metastatic spread

* Correspondence: F. Mechta-Grigoriou (ORCID Number: 0000-0002-3751-6989) Phone: +33 (0)1 56 24 66 53; Fax: +33 (0)1 56 24 66 50; E-mail address: fatima.mechta-grigoriou@curie.fr

Supplementary Fig. 1

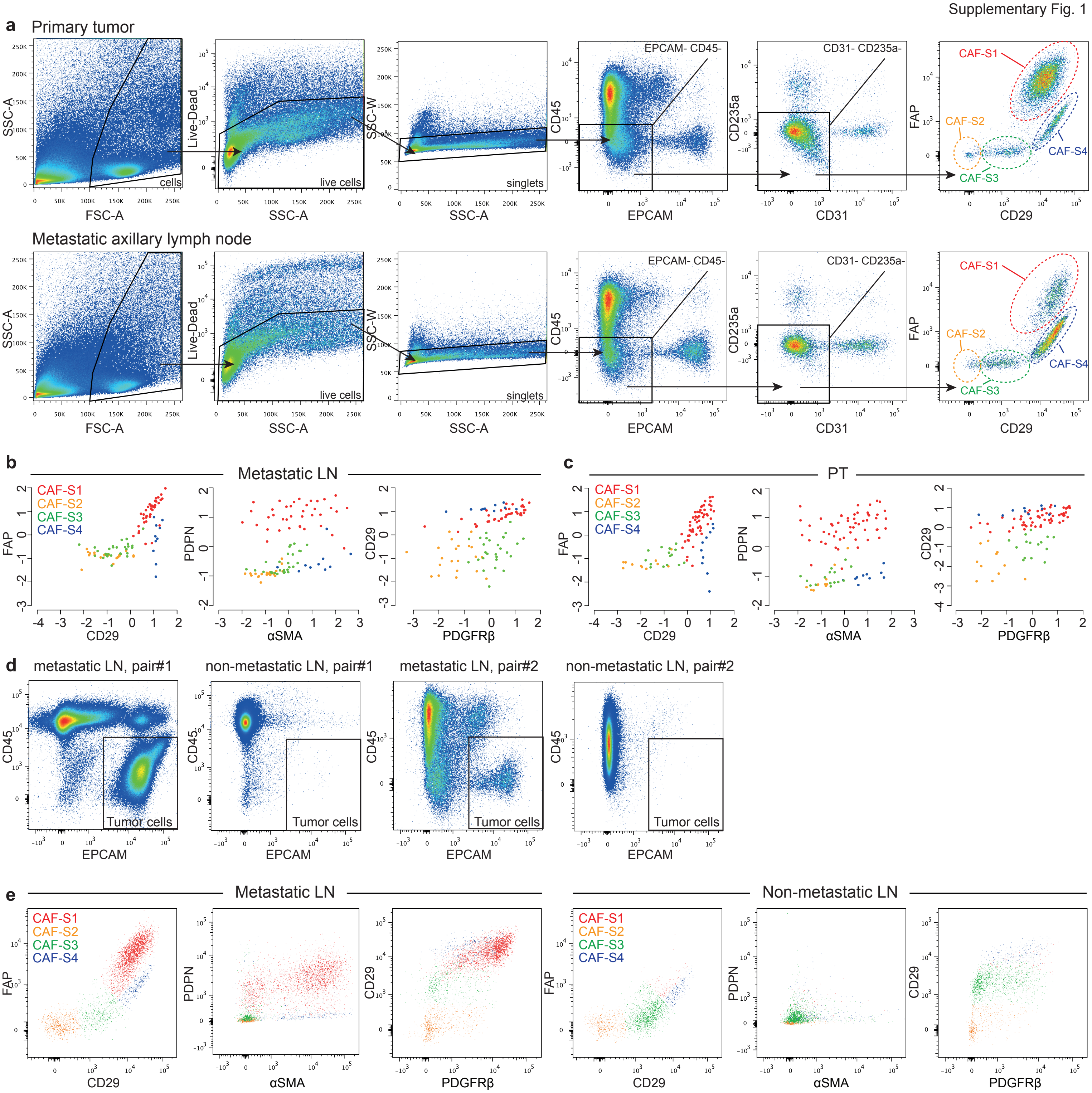

Supplementary Figure 1 Gating strategy of the 4 CAF subsets by FACS (related to Fig. 1) (**a**) Gating strategy to identify CAF subsets in BC PT (top) and its corresponding invaded axillary LN (bottom), shown in Fig. 1a. Cells were gated on $DAPI^-$ $EPCAM^-$ $CD45^-$ $CD31^-$ $CD235a^-$ to exclude dead, epithelial, hematopoietic, endothelial and red blood cells, respectively. (**b**) Scatter plots showing FAP, CD29, PDPN, αSMA, and PDGFRβ staining from FlowSom output. Each dot represents a node from FlowSom tree (shown in Fig. 1b) from invaded LN and colors match the cluster (i. e. CAF subset) to which the node belongs. For each CAF marker, staining intensities are FACS speMFI centered and reduced within FlowSom algorithm. FlowSom plots validate the CAF subsets manual gating that we performed, as shown with the correspondence with Fig. 1a (n = 20 LN). (**c**) Same as in (**b**) for primary tumors (PT) and FlowSom tree shown in Fig. 1c (n = 16 PT). (**d**) Representative FACS plots showing CD45 and EPCAM staining in 2 pairs of metastatic and non-metastatic axillary LN. (**e**) Representative FACS plots of FAP, CD29, PDPN, αSMA, and PDGFRβ on $DAPI^-$ $EPCAM^-$ $CD45^-$ $CD31^-$ $CD235a^-$ cells from a metastatic LN (Left) and its corresponding non-invaded axillary LN (Right). Source data are provided as a Source Data file, as well as R scripts used to generate the figure panels.

Supplementary Fig. 2

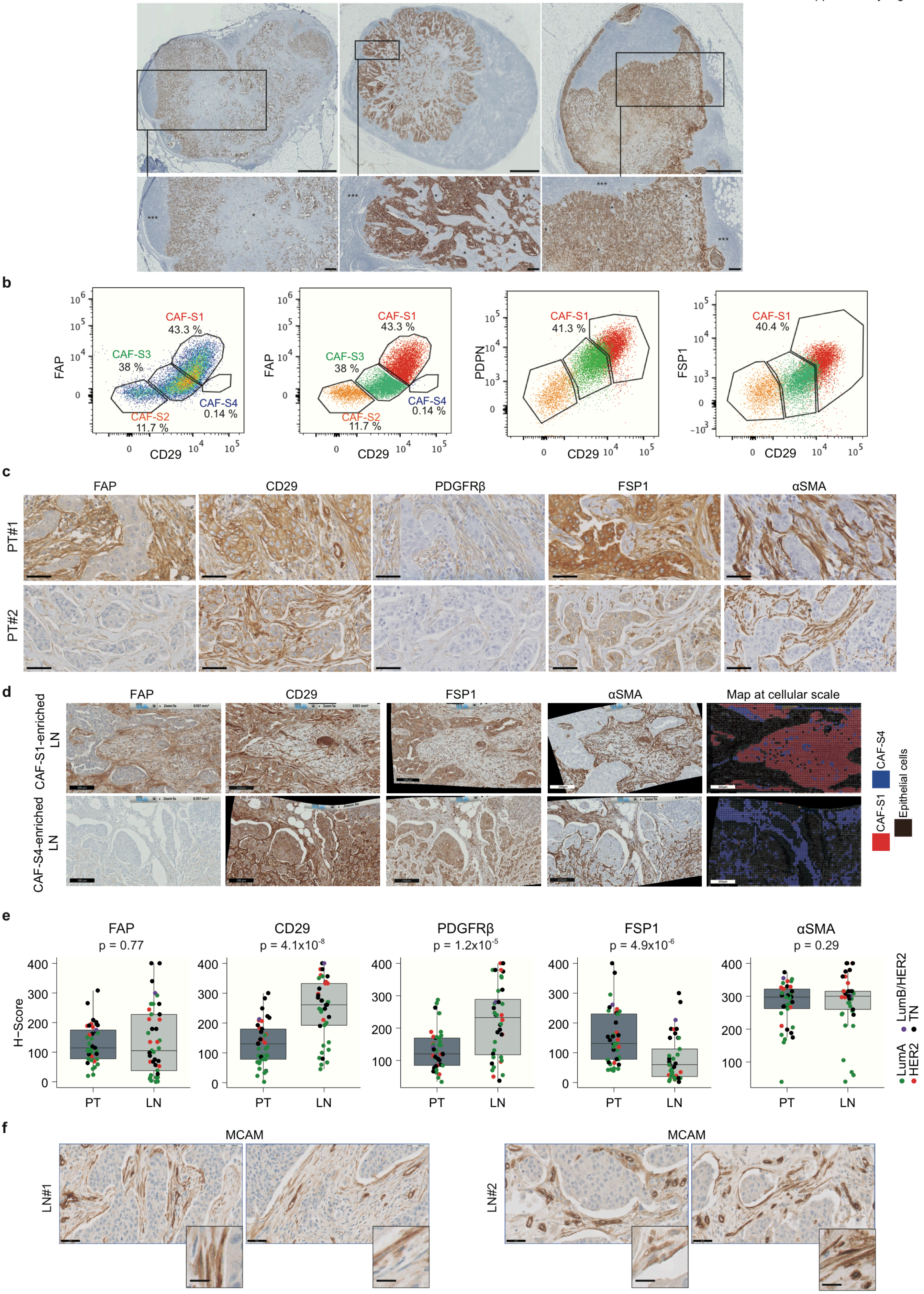

Supplementary Figure 2 Histological staining of CAF markers (related to Figs. 2 and 3) (**a**) Representative images of EPCAM staining from Lum A, HER2 and TN LN revealing metastatic zones. In high-magnification insets, *** defines non-metastatic zones and * shows metastatic zones that were considered for the analyses. Scale bars, 1 mm (Lum A and TN LN) and 2.5 mm (HER2 LN). Scale bars in insets, 200 μm. (**b**) Representative FACS plots showing FAP, CD29, PDPN, and FSP1 protein levels in DAPI$^-$ EPCAM$^-$ CD45$^-$ CD31$^-$ CD235a$^-$ cells from a BC. The CAF-S1 subset and its percentage (%) are shown. (**c**) Representative images of FAP, CD29, PDGFRβ, FSP1 and αSMA staining on serial sections of two PT cases corresponding to the 2 metastatic LN shown in Fig. 2c. Scale bar, 50 μm. (**d**) Representative views of FAP, CD29, FSP1, αSMA immunostaining of serial consecutive sections from invaded LN from BC patients, used for defining maps of CAF subsets at cellular scale. Examples from different patients of CAF-S1- or CAF-S4-enriched LN are shown. Each CAF subset is represented by a color code (red, CAF-S1; blue, CAF-S4) and epithelial tumor cells are in black. These images were reconstructed by applying mathematical modeling (see Methods and decision tree shown in Fig. 2d) on staining intensities of CAF markers in each cell analyzed on serial sections. Scale bar, 200 μm. (**e**) CAF marker H-Scores in PT and matched LN (n = 41 pairs). Boxplots are median ± 25%-75% quantiles, whisker values range 1.5 × IQR above 75th or below 25th percentiles. p values from Wilcoxon signed rank test. Dot colors represent BC subtypes, Lum A in green, Lum B in blue, HER2 in red, Lum B/HER2 in purple, TN in black. (**f**) Representative views of MCAM/CD146 immunostaining in invaded LN. Two different BC patients are shown. Scale bar, 50 μm, with 25 μm in inset. Source data are provided as a Source Data file, as well as R scripts used to generate the figure panels.

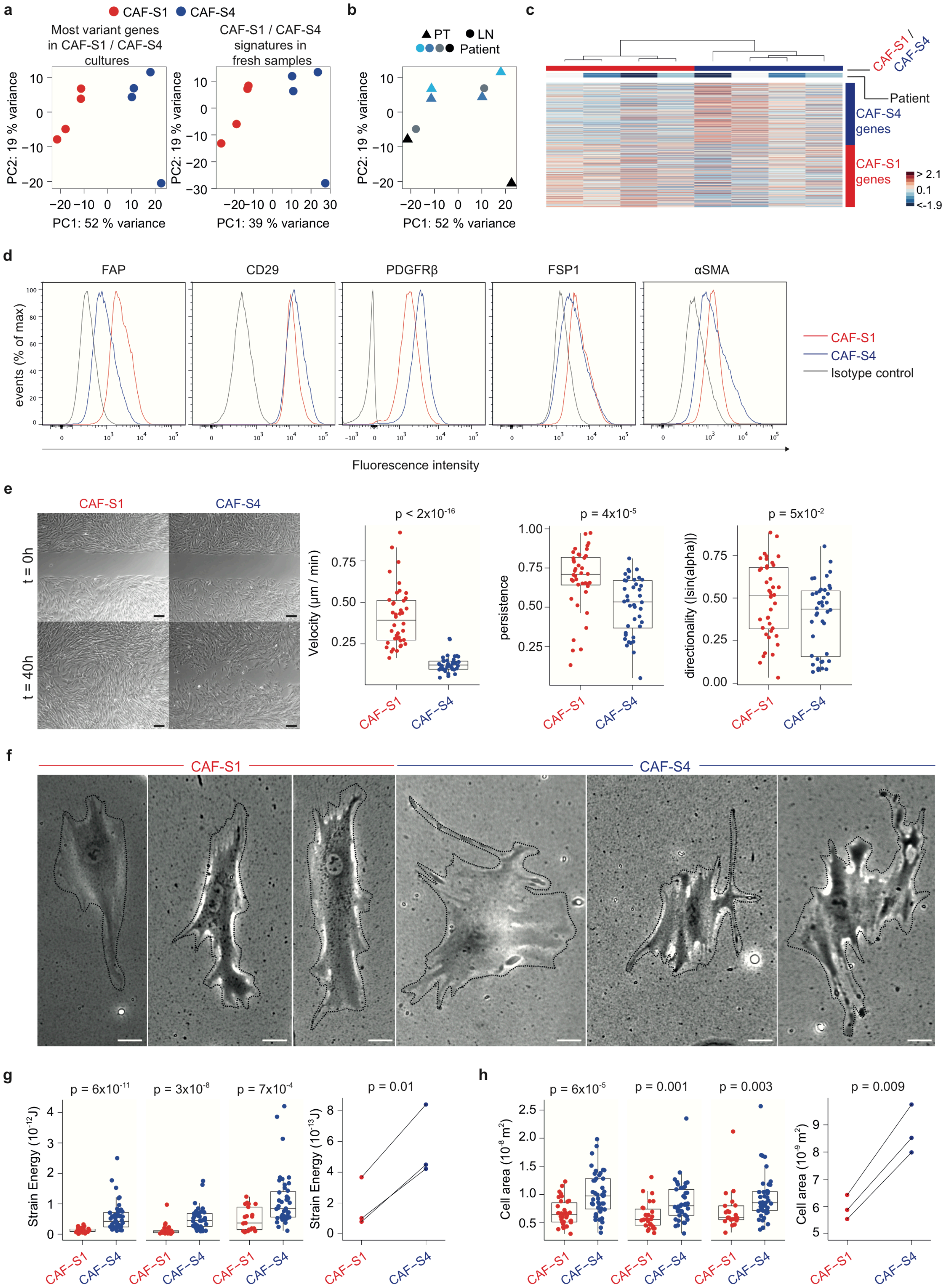
a
CAF-S1 CAF-S4
Most variant genes in CAF-S1 / CAF-S4 cultures
CAF-S1 / CAF-S4 signatures in fresh samples
PC2: 19 % variance
PC1: 52 % variance
PC1: 39 % variance
b
PT LN
Patient
c
CAF-S1 / CAF-S4
Patient
CAF-S4 genes
CAF-S1 genes
> 2.1
0.1
<-1.9
d
FAP
CD29
PDGFRβ
FSP1
αSMA
events (% of max)
Fluorescence intensity
CAF-S1
CAF-S4
Isotype control
e
CAF-S1
CAF-S4
t = 0h
t = 40h
p < 2x10-16
p = 4x10-5
p = 5x10-2
Velocity (µm / min)
persistence
directionality (|sin(alpha)|)
f
CAF-S1
CAF-S4
g
p = 6x10-11
p = 3x10-8
p = 7x10-4
p = 0.01
Strain Energy (10-12 J)
Strain Energy (10-13 J)
h
p = 6x10-5
p = 0.001
p = 0.003
p = 0.009
Cell area (10-8 m2)
Cell area (10-9 m2)

Supplementary Figure 3 CAF-S1 and CAF-S4 fibroblasts show distinct properties *in vitro* (related to Fig. 4)

(**a**) PCA analysis of CAF-S1 and CAF-S4 primary cultures (n = 4 pairs of CAF-S1 and CAF-S4, each pair being isolated from the same patient). Left, PCA based on the 500 most variant genes from RNAseq data of primary cultures. Right, PCA based on top 500 gene-signatures from RNAseq data of fresh samples (as shown in Fig. 3). (**b**) Same PCA as in (**a**, left) showing the patient and tissue of origins of each CAF-S1 and CAF-S4 primary cultures. (**c**) Hierarchical clustering using Ward's method with Pearson distances on the same primary CAF-S1 and CAF-S4 as in (**a**). Columns represent *in vitro* cultured CAF-S1 and CAF-S4 cells and lines stand for genes of CAF-S1 and CAF-S4 top 500 gene-signatures from samples. Color saturation shows gene expression deviation from the mean (above in red, below in blue). (**d**) Representative FACS plots of FAP, CD29, PDGFRβ, FSP1 and αSMA protein levels from primary CAF-S1 and CAF-S4 cultures (paired samples). (**e**) Cell exclusion zone assay (independent experiment using other CAF-S1 and CAF-S4 cells as those tested in Fig. 4c). Left, Representative images of a free-zone (t0, top) closing after 40 h (bottom) of CAF subset migration. Scale bar, 200 µm. Graphs show velocity (left), persistence (middle) and direction (|sin(α)|, right), which were assessed by tracking 40 CAF-S1 and 40 CAF-S4 cells. p values from Mann-Whitney test. 1 representative experiment is shown. (**f-h**) Traction Force Microscopy. (**f**) Phase contrast images of cells shown in Fig. 4f. Scale bar, 20 µm. (**g**) Left, Strain energy (expressed in Joules, J) of CAF-S1 and CAF-S4 pairs (each dot represents a single cell, n ≥ 21 cells per CAF subset, corresponding to Fig. 4g). p values from Mann-Whitney test. Right, Same as left panel but showing median strain energy per CAF subset (n = 3). p value from paired t-test. (**h**) Left, Cell area of CAF-S1 and CAF-S4 cells (as in **g**). p values from Mann-Whitney test. Right, Same as left panel but showing median areas per CAF subset (n = 3 independent experiments). p value from paired t-test. In all panels, boxplots are medians ± 25%-75% quantiles, whisker values range 1.5 × IQR above 75th or below 25th percentiles. Source data are provided as a Source Data file, as well as R scripts used to generate the figure panels.

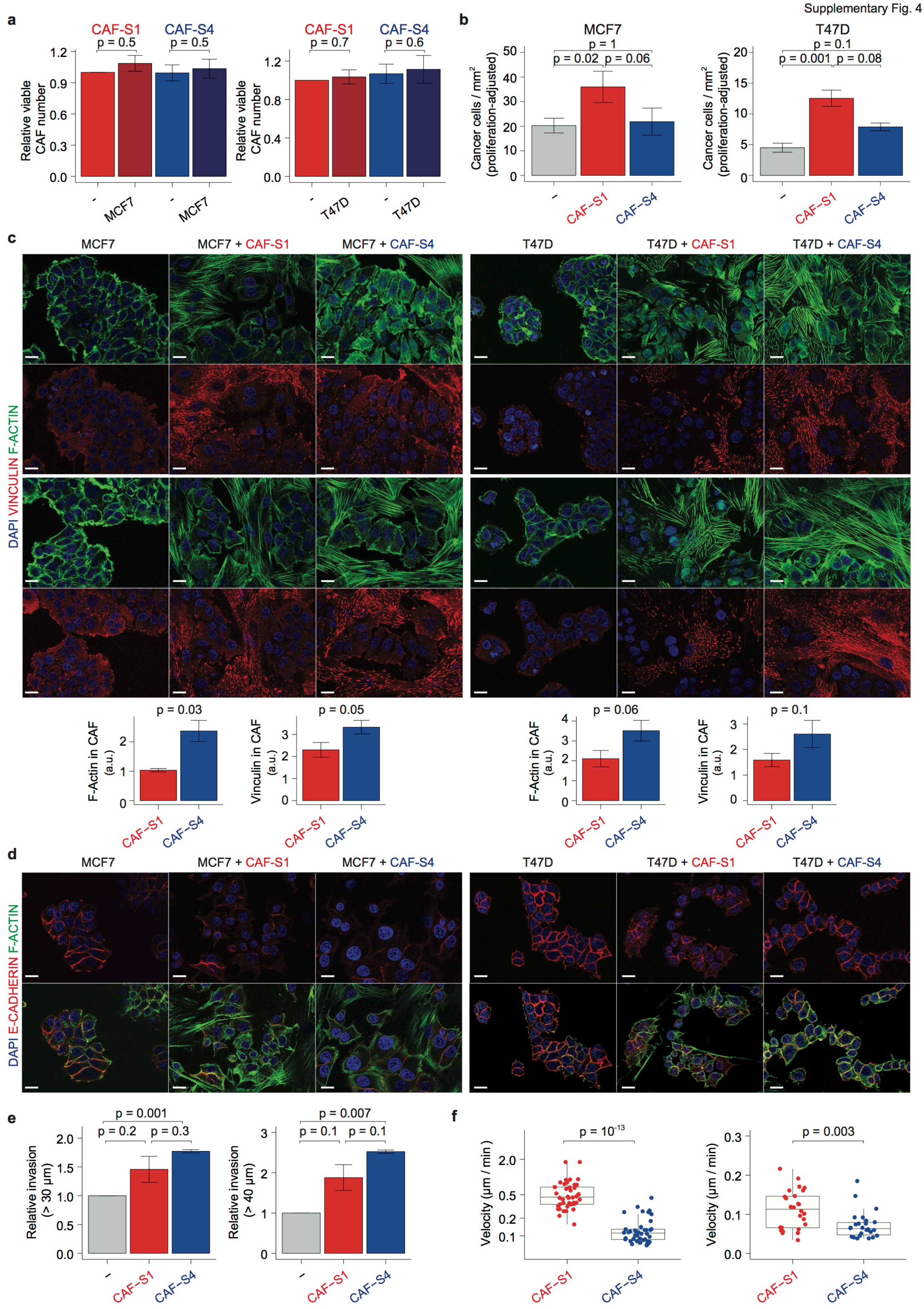
a
CAF-S1
CAF-S4
p = 0.5
p = 0.5
Relative viable CAF number
MCF7
MCF7
p = 0.7
p = 0.6
T47D
T47D
b
MCF7
p = 1
p = 0.02
p = 0.06
Cancer cells / mm2 (proliferation-adjusted)
T47D
p = 0.1
p = 0.001
p = 0.08
c
MCF7
MCF7 + CAF-S1
MCF7 + CAF-S4
T47D
T47D + CAF-S1
T47D + CAF-S4
DAPI VINCULIN F-ACTIN
p = 0.03
F-Actin in CAF (a.u.)
p = 0.05
Vinculin in CAF (a.u.)
p = 0.06
p = 0.1
d
DAPI E-CADHERIN F-ACTIN
e
p = 0.001
p = 0.2
p = 0.3
Relative invasion (> 30 μm)
p = 0.007
p = 0.1
p = 0.1
Relative invasion (> 40 μm)
f
p = 10-13
Velocity (μm / min)
p = 0.003

Supplementary Figure 4 CAF-S1 and CAF-S4 modulate BC cell phenotype and invasion capabilities (related to Fig. 5 and 6)

(**a**) Impact of CAF-S1 or CAF-S4 co-culture with MCF7 (left, n = 7 independent experiments) and T47D (right, n = 6) co-culture with CAF-S1 or CAF-S4 on the total number of viable (Dapi^{-} assessed by FACS) CAF subset relative to control (-, CAF-S1 without cancer cells (red bar) considered as the reference). p values from Wilcoxon signed rank test. (**b**) CAF-S1 and CAF-S4 chemo-attraction capacities evaluated by Transwell assays on MCF7 (left, n ≥ 10 independent experiments) and T47D (right, n ≥ 8), corrected by their pro-proliferation properties. p values from Wilcoxon signed rank test. (**c**) Images of F-actin (green, top) or Vinculin (red, bottom) with DAPI (blue) staining in MCF7 (left) and T47D (right) cultured alone, or in presence of CAF-S1 or CAF-S4. Merge images are shown in Fig. 5e. Scale bars, 20 µm. Quantifications show F-Actin and Vinculin staining in CAF subset per CAF cell area (F-Actin and Vinculin signal areas defined on ImageJ, divided by CAF surface and expressed in arbitrary units (a.u.). Evaluation on at least 5 images per CAF subset (CAF subset co-cultured with MCF7 (left) or T47D (right)). p values from Mann-Whitney test (F-Actin far left graph) and Student's t-test (all other 3 graphs). (**d**) Representative images (different from those shown in Fig. 5f) showing staining of E-Cadherin (red) and DAPI (blue) (top), or co-staining of E-Cadherin (red), F-actin (green) and DAPI (blue) (bottom) in MCF7 or T47D cultured alone, or in presence of CAF-S1 or CAF-S4. Scale bars, 20 µm. (**e**) Proportion of BC cells that invaded (in Transwell inverted assay) above 30 µm (left) and 40 µm (right) in CAF-S1- or CAF-S4-embedded collagen, relative to CAF-free condition (n = 3 independent experiments, ~500 BC cells per analyzed z-stack). p values from paired t-test. (**f**) CAF-S1 and CAF-S4 velocity assessed in tumor-on-chip devices. The graphs display 2 different pairs, each dot represents a single cell (n ≥ 27 per CAF subset). p values from Mann-Whitney test. Boxplots are median ± 25%-75% quantiles, whisker values range 1.5 × IQR above 75th or below 25th percentiles. Barplots are mean ± SEM. At least 2 CAF-S1 and CAF-S4 pairs have been tested. Source data are provided as a Source Data file, as well as R scripts used to generate the figure panels.

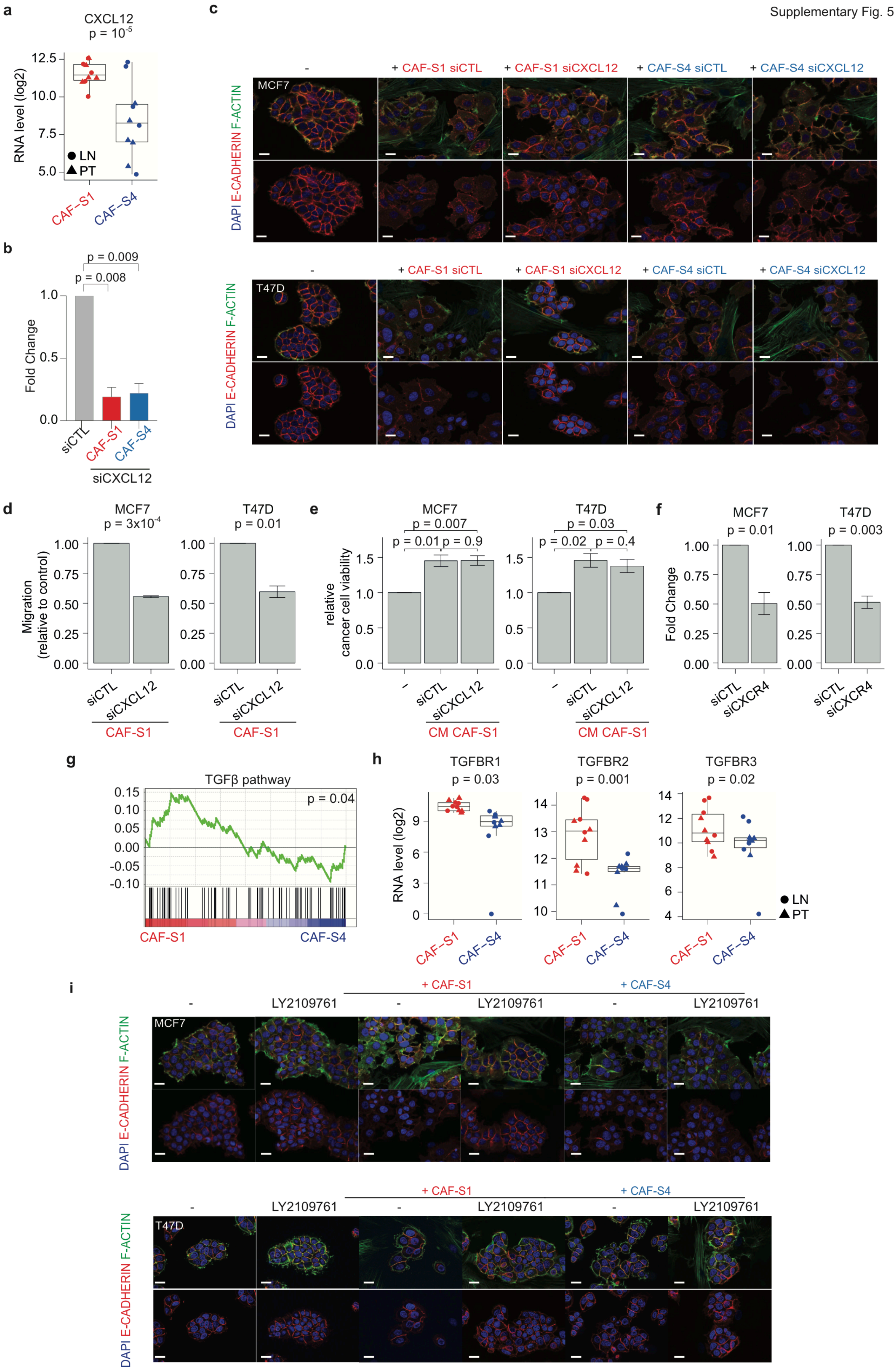
a
CXCL12
p = 10^-5
RNA level (log2)
12.5
10.0
7.5
5.0
LN
PT
CAF-S1
CAF-S4
b
p = 0.009
p = 0.008
Fold Change
1.0
0.5
0.0
siCTL
CAF-S1
CAF-S4
siCXCL12
c
-
+ CAF-S1 siCTL
+ CAF-S1 siCXCL12
+ CAF-S4 siCTL
+ CAF-S4 siCXCL12
MCF7
T47D
DAPI E-CADHERIN F-ACTIN
DAPI E-CADHERIN
d
MCF7
p = 3x10^-4
T47D
p = 0.01
Migration (relative to control)
1.00
0.75
0.50
0.25
0.00
siCTL
siCXCL12
CAF-S1
e
MCF7
p = 0.007
p = 0.01
p = 0.9
T47D
p = 0.03
p = 0.02
p = 0.4
relative cancer cell viability
1.5
1.0
0.5
0.0
-
siCTL
siCXCL12
CM CAF-S1
f
MCF7
p = 0.01
T47D
p = 0.003
Fold Change
siCTL
siCXCR4
g
TGFβ pathway
p = 0.04
0.15
0.10
0.05
0.00
-0.05
-0.10
CAF-S1
CAF-S4
h
TGFBR1
p = 0.03
TGFBR2
p = 0.001
TGFBR3
p = 0.02
RNA level (log2)
LN
PT
CAF-S1
CAF-S4
i
+ CAF-S1
+ CAF-S4
-
LY2109761
MCF7
T47D
DAPI E-CADHERIN F-ACTIN
DAPI E-CADHERIN

Supplementary Figure 5 TGFβ and CXCL12 pathways are involved in CAF-S1-mediated BC cell invasion (related to Fig. 7)

(**a**) CXCL12 mRNA levels in CAF-S1 and CAF-S4 (n = 10) assessed by RNAseq. p values from DESeq2. (**b**) CXCL12 mRNA levels after silencing of CXCL12 in CAF-S1 and CAF-S4. Data are shown as fold change to control (n = 3). p value from Student t-test. (**c**) Representative images showing immunofluorescence co-staining of E-Cadherin (red), F-actin (green) and DAPI (blue) in MCF7 (Up) or T47D (Down) cultured alone (-), or in presence of CAF-S1 or CAF-S4 transfected with non-targeting siRNA (siCTL) or with siRNA targeting CXCL12 (siCXCL12). Scale bars, 20 µm. (**d**) Impact of CXCL12 silencing in CAF-S1 on CAF-S1 capacities to chemo-attract MCF7 (left, n = 3) and T47D (right, n = 3) assessed by Transwell assays. Data are shown as fold change to control and p values are from one sample t-tests. (**e**) Impact of 24 h treatment with conditioned medium (CM) from CAF-S1 transfected with non-targeting siRNA (siCTL) or from CAF-S1 silenced for CXCL12 (siCXCL12) on the total number of viable MCF7 (left, n = 4) and T47D (right, n = 4) (assessed by Resazurin staining) relative to control (without CAF-S1). p values from paired t-tests. (**f**) CXCR4 mRNA levels after silencing of CXCR4 in MCF7 (left) and T47D (right). Data are shown as fold change to control (n = 4 independent experiments). p value from one sample t-tests. (**g**) Gene set enrichment analysis of TGFβ pathway in CAF-S1 *versus* CAF-S4 transcriptomic signatures. p refers to false discovery rate q-value. (**h**) TGFBR1, -2 and -3 mRNA levels in CAF-S1 and CAF-S4 (n = 10) assessed by RNAseq. p values from DESeq2. (**i**) Same as in (**c**) with/without CAF-S1 or CAF-S4 and with/without TGFβR inhibitor (LY2109761). In all panels, at least 3 CAF-S1 and CAF-S4 pairs have been analyzed. Each pair of CAF-S1 and CAF-S4 was isolated from the same patient. Source data are provided as a Source Data file, as well as R scripts used to generate the figure panels.

Supplementary Fig. 6

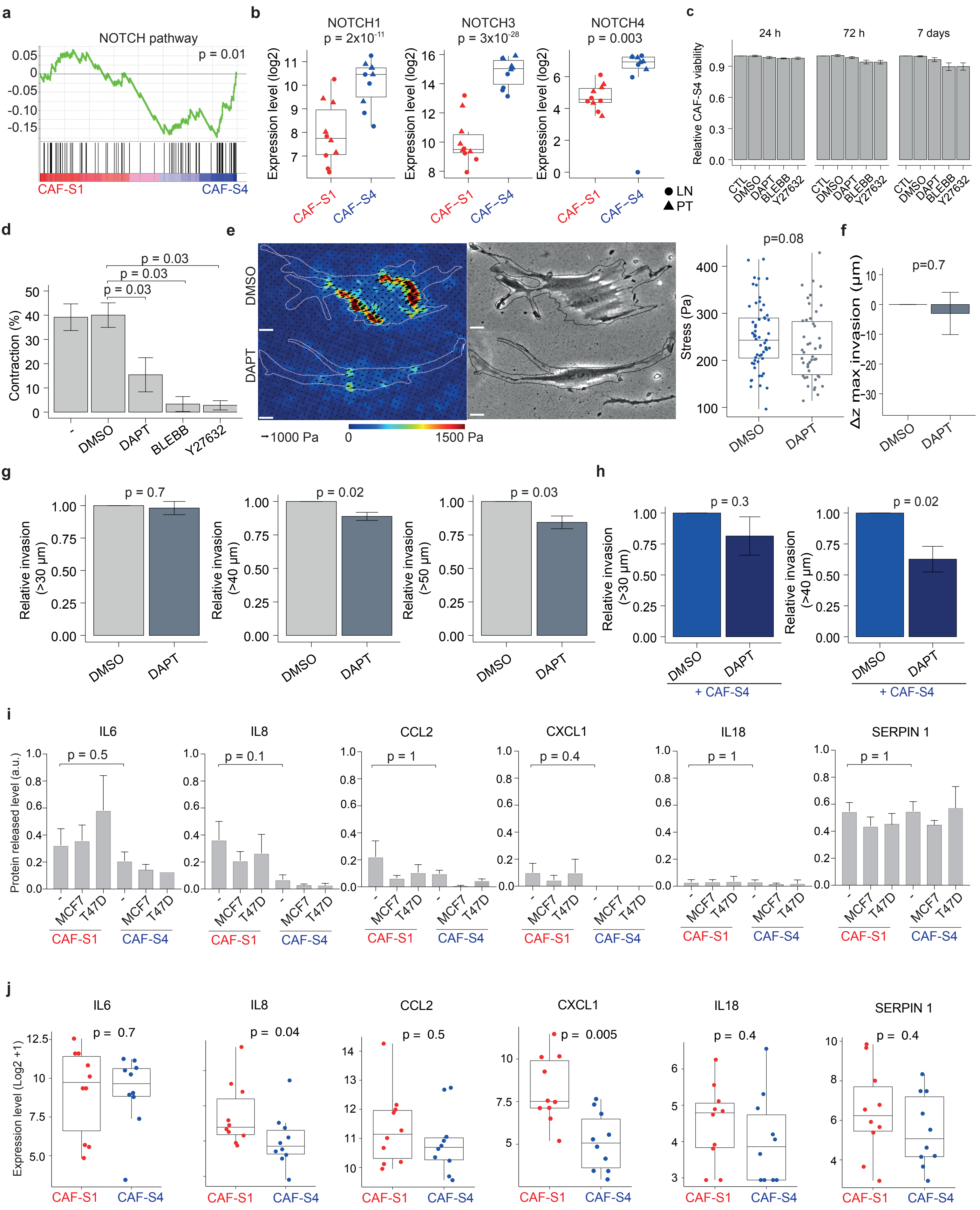

Supplementary Figure 6 NOTCH pathway contributes to CAF-S4-mediated ECM contraction (related to Fig. 7)

(**a**) Gene set enrichment analysis of NOTCH pathway in CAF-S1 *versus* CAF-S4 transcriptomic signatures. p refers to false discovery rate q-value. (**b**) NOTCH1, -3 and -4 mRNA levels in CAF-S1 and CAF-S4 (n = 10) assessed by RNAseq. p values from DESeq2. (**c**) Impact of 24 h to 7 days DAPT, Blebbistatin (BLEBB) or Y27632 treatment on CAF-S4 viability relative, assessed by Resazurin staining, to untreated control (CTL) (n = 6 independent experiments). (**d**) Percentage (%) of collagen gel contraction by CAF-S4 upon DAPT, Blebbistatin (BLEBB) and Y27632 treatment (n = 6 independent experiments). p values from Wilcoxon signed rank test. (**e**) Contractility of CAF-S4 without or with DAPT treatment assessed by Traction Force Microscopy. Representative images of traction stress applied by CAF-S4 on their substrate (left) upon DAPT treatment. Traction forces (arrows) and cell outlines (dashed white lines) are shown. Color represents traction stress magnitude (Pascal, Pa). The corresponding phase contrast images are shown on right. Scale bar, 20 µm. Right, Traction stress developed by CAF-S4 upon DAPT treatment (each dot is a single cell, corresponding to Fig. 6e). p value from Mann-Whitney test. (**f**) Maximal vertical distance browsed by BC cells in collagen, in presence of DAPT relative to control (no DAPT). (n = 5 independent experiments. ~700 BC cells per analyzed z-stack). p value from paired t-test. (**g**) Proportion of BC cells that invaded above 30 µm (left), 40 µm (middle) and 50 µm (right) in collagen, in presence of DAPT relative to control condition (n = 5 independent experiments, ~700 BC cells per analyzed z-stack). p values from paired t-test. (**h**) Proportion of BC cells that invaded above 30 µm (left) and 40 µm (right) in CAF-S4-embedded collagen, in presence of DAPT relative to control condition (n = 6 independent experiments, ~700 BC cells per analyzed z-stack). p values from paired t-test. In all panels, boxplots are median ± 25%-75% quantiles, whisker values range 1.5 × IQR above 75th or below 25th percentiles. Barplots mean ± SEM. In all panels, at least 3 CAF-S1 or CAF-S4 cell lines have been tested, except S5C (MCF7) where 2 CAF-S1 cell lines and S5K where 1 CAF-S4 cell line were used. (**i**) Levels of cytokine release in supernatants collected from CAF-S1 and CAF-S4

fibroblasts cultured either alone (-) or in presence of MCF7 or T47D, as indicated. Cytokine release is measured using cytokine antibody-paired array and assessed by the following ratio: Cytokine intensity mean (intensity mean of 2 spots per cytokine per experiment) / Control mean intensity (2 spots of 3 internal controls). P values from Mann-Whitney test (n = 3). In all panels, at least 2 CAF-S1 and CAF-S4 pairs have been analyzed. Each pair of CAF-S1 and CAF-S4 was isolated from the same patient. (**j**) Boxplots showing RNA levels of the genes tested in the cytokine antibody array shown in (**i**) in CAF-S1 and CAF-S4 cells (n = 20). Data are from RNA-seq. P values from Mann-Whitney test. Expression of G-CSF, GM-CSF and MIF was also analyzed but was not detected at either protein or RNA levels. Source data are provided as a Source Data file, as well as R scripts used to generate the figure panels.

Supplementary Table 1. Clinical description of the prospective cohorts of breast cancer patients

		FACS: Paired samples	FACS: PT	FACS: LN	RNAseq: Paired PT/LN	Culture: PT	Culture: LN
Number of patients		15 (100 %)	16 (100 %)	20 (100 %)	5 (100 %)	14 (100%)	8 (100 %)
Gender	Female	15 (100 %)	16 (100 %)	20 (100 %)	5 (100 %)	14 (100%)	8 (100 %)
	Male	0	0	0	0	0	0
Inclusion		2015-2017	2015-2017	2015-2017	2015-2016	2016-2018	2016-2017
Follow-up (years)	median	1.2	1.2	1.6	2.0	1.0	1.1
	range	0.9 - 2.4	0.1 - 2.4	0.5 - 2.4	1.1 - 2.3	0.3 - 1.7	0.4 - 1.4
Age at diagnosis (years)	median	51.7	56.2	52.0	78.6	54.8	77.0
	range	38.6 - 91.2	38.6 - 91.2	38.6 - 91.2	45.7 - 85.8	45.6 - 80.0	43.9 - 89.8
Sample localization	Tumor	15	16	/	5	14	/
	invaded LN	15	/	20	5	/	8
Size of infiltrating tumor (mm)	median	23	24	22.5	30	23	24
	range	13-80	13-80	10-80	23-50	19-42	17-80
Histological Grade (EE)	I	0	0	1 (5 %)	0	2 (14.3 %)	1 (12.5 %)
	II	7 (46.7 %)	7 (43.8 %)	9 (45 %)	4 (80 %)	9 (64.3 %)	2 (25 %)
	III	8 (53.3 %)	9 (56.2 %)	10 (50 %)	1 (20 %)	3 (21.4 %)	5 (62.5 %)
Pathological tumor Size pT	pT0	0	0	0	0	0	0
	pT1	5 (33.3 %)	5 (31.2 %)	8 (40 %)	0	3 (21.4 %)	3 (37.5 %)
	pT2	7 (46.7 %)	8 (50.0 %)	9 (45 %)	5 (100 %)	11 (78.6 %)	4 (50 %)
	pT3	3 (20.0 %)	3 (18.8 %)	3 (15 %)	0	0	1 (12.5 %)
	pT4	0	0	0	0	0	0
Pathological Lymph node status pN	pN0	0	1 (6.2 %)	0	0	5 (35.7 %)	0
	pN1	8 (53.3 %)	8 (50.0 %)	10 (50 %)	2 (40 %)	6 (42.9 %)	3 (37.5 %)
	pN2	3 (20.0 %)	3 (18.8 %)	5 (25 %)	3 (60 %)	2 (14.3 %)	2 (25 %)
	pN3	4 (26.7 %)	4 (25.0 %)	5 (25 %)	0	1 (7.1 %)	3 (37.5 %)
	NA	0	0	0	0	0	0
Metastasis status pM	M0	13 (86.7 %)	13 (81.3 %)	17 (85 %)	5 (100 %)	12 (85.7 %)	5 (62.5 %)
	M1	1 (6.7 %)	1 (6.2 %)	1 (5 %)	0	1 (7.1 %)	3 (37.5 %)
	NA	1 (6.7 %)	2 (12.5 %)	2 (10%)	0	1 (7.1 %)	0
Late distant metastases	M0	14 (93.3 %)	14 (87.5 %)	19 (95 %)	3 (60 %)	14 (100%)	7 (87.5 %)
	M1	1 (6.7 %)	1 (6.2 %)	1 (5 %)	2 (40 %)	0	1 (12.5 %)
	NA	0	1 (6.2 %)	0	0	0	0
Subtype	LumA	2 (13.3 %)	2 (12.5 %)	5 (25 %)	1 (20 %)	6 (42.9 %)	0
	LumB	8 (53.3 %)	8 (50.0 %)	10 (50 %)	3 (60 %)	7 (50 %)	3 (37.5 %)
	HER2	4 (26.7 %)	4 (25.0 %)	4 (20 %)	1 (20 %)	1 (7.1 %)	3 (37.5 %)
		2 LumB/HER2 included	2 LumB/HER2 included	2 LumB/HER2 included		1 LumB/HER2 included	2 LumB/HER2 included
	TN	0	1 (6.2 %)	0	0	0	1 (12.5 %)
	NA	1 (6.7 %)	1 (6.2 %)	1 (5 %)	0	0	1 (12.5 %)
Hormonotherapy	Yes	12 (80.0 %)	12 (75 %)	16 (80 %)	4 (80 %)	12 (85.7 %)	6 (75%)
	No	3 (20.0 %)	3 (18.8 %)	4 (20 %)	1 (20 %)	2 (14.3 %)	2 (25 %)
	NA	0	1 (6.2 %)	0	0	0	0
Radiotherapy	Yes	13 (86.7 %)	13 (81.3 %)	17 (85 %)	5 (100 %)	12 (85.7 %)	7 (87.5 %)
	No	2 (13.3 %)	2 (12.5 %)	3 (15 %)	0	2 (14.3 %)	1 (12.5 %)
	NA	0	1 (6.2 %)	0	0	0	0
Chemotherapy	Yes	13 (86.7 %)	13 (81.3 %)	18 (90 %)	3 (60 %)	6 (42.9 %)	4 (50 %)
	No	2 (13.3 %)	2 (12.5 %)	2 (10%)	2 (40 %)	8 (57.1 %)	4 (50 %)
	NA	0	1 (6.2 %)	0	0	0	0
Targeted Therapy	Yes	5 (33.3 %)	5 (31.2 %)	5 (25 %)	1 (20 %)	0	2 (25 %)
	No	10 (66.7 %)	10 (62.5 %)	15 (75 %)	4 (80 %)	14 (100%)	6 (75 %)
	NA	0	1 (6.2 %)	0	0	0	0

Supplementary Table 1 Clinical description of the prospective cohorts of BC patients (Related to Figs. 1, 3-7)

PT and invaded LN samples have been used for multicolor flow cytometry, RNAseq or culture of primary CAF-S1 and CAF-S4 fibroblasts. For FACS samples, paired samples are included in the 16 PT and 20 LN samples. Source data are provided as a Source Data file, as well as R scripts used to generate the figure panels.

Supplementary Table 2. Clinical description of the retrospective cohorts of breast cancer patients

		LN	Paired cohort PT/LN	Unpaired cohort PT (N+)	Unpaired cohort LN
Number of patients		124 (100%)	41 (100%)	75 (100%)	84 (100%)
Gender	female	123 (99.2 %)	40 (97.6 %)	74 (98.7 %)	83 (98.8 %)
	male	1 (0.8 %)	1 (2.4 %)	1 (1.3 %)	1 (1.2 %)
Inclusion		2004-2006	2004-2006	2004-2012	2004-2006
Follow-up (years)	median	9.3	9.2	9.2	8.9
	range	0.02 - 13.1	0.07 - 13.1	0.07 - 13.1	0.07-13.1
Age at diagnosis (years)	median	56.9	56.1	55.9	57.3
	range	26.4 - 84.4	26.4 - 84.4	22.4 - 84.4	26.4 - 84.4
Sample localization	Tumor	/	41	75	/
	invaded LN	124	41	/	84
Size of infiltrating tumor (mm)	median	22	20	19	21
	range	8 - 90	8 - 45	7 - 55	8 - 60
Histological Grade (EE)	I	16 (12.9 %)	7 (17.1 %)	16 (21.3 %)	12 (14.3 %)
	II	30 (24.2 %)	12 (29.3 %)	23 (30.7 %)	20 (23.8 %)
	III	78 (62.9 %)	22 (53.6 %)	36 (48.0 %)	52 (61.9 %)
Pathological tumor Size pT	pT0	0	0	0	0
	pT1	57 (46.0 %)	22 (53.6 %)	48 (64.0 %)	41 (48.8 %)
	pT2	56 (45.2 %)	17 (41.5 %)	24 (32.0 %)	34 (40.5 %)
	pT3	8 (6.4 %)	0	1 (1.3 %)	6 (7.1 %)
	pT4	3 (2.4 %)	2 (4.9 %)	2 (2.7 %)	3 (3.6 %)
Pathological Lymph node status pN	pN0	0	0	0	0
	pN1	68 (54.8 %)	26 (63.4 %)	55 (73.3 %)	49 (58.4 %)
	pN2	42 (33.9 %)	11 (26.8 %)	15 (20.0 %)	27 (32.1 %)
	pN3	14 (11.3 %)	4 (9.8 %)	4 (5.4 %)	8 (9.5 %)
	NA	0	0	1 (1.3 %)	0
Metastasis status pM	M0	116 (93.6 %)	39 (95.2 %)	73 (97.4 %)	78 (92.8 %)
	M1	5 (4 %)	1 (2.4 %)	1 (1.3 %)	4 (4.8 %)
	NA	3 (2.4 %)	1 (2.4 %)	1 (1.3 %)	2 (2.4 %)
Late distant metastases	M0	87 (70.2 %)	28 (68.3 %)	59 (78.7 %)	58 (69.0 %)
	M1	33 (26.6 %) **	12 (29.3 %)	15 (20.0 %)	24 (28.6 %)
	NA	4 (3.2 %)	1 (2.4 %)	1 (1.3 %)	2 (2.4 %)
Subtype	LumA	30 (24.2 %)	19 (46.3 %)	38 (50.7 %)	30 (35.7 %)
	LumB	40 (32.2 %)	0	0	0
	HER2	26 (21.0 %)	8 (19.5 %)	16 (21.3 %)	26 (31.0 %)
		4 LumB/HER2 included	1 LumB/HER2 included	2 LumB/HER2 included	4 LumB/HER2 included
	TN	28 (22.6 %)	14 (34.2 %)	21 (28.0 %)	28 (33.3 %)
Hormonotherapy	Yes	64 (51.6 %)	17 (41.5 %)	35 (46.7 %)	31 (36.9 %)
	No	58 (46.8 %)	23 (56.1 %)	39 (52.0 %)	52 (61.9 %)
	NA	2 (1.6 %)	1 (2.4 %)	1 (1.3 %)	1 (1.2 %)
Radiotherapy	Yes	115 (92.8 %)	39 (95.2 %)	72 (96.0 %)	79 (94.0 %)
	No	6 (4.8 %)	1 (2.4 %)	2 (2.7 %)	4 (4.8 %)
	NA	3 (2.4 %)	1 (2.4 %)	1 (1.3 %)	1 (1.2 %)
Chemotherapy	Yes	106 (85.5 %)	35 (85.4 %)	56 (74.7 %)	70 (83.3 %)
	No	16 (12.9 %)	5 (12.2 %)	18 (24.0 %)	13 (15.5 %)
	NA	2 (1.6 %)	1 (2.4 %)	1 (1.3 %)	1 (1.2 %)
Targeted Therapy	Yes	27 (21.8 %)	8 (19.5 %)	15 (20.0 %)	26 (31.0 %)
	No	95 (76.6 %)	32 (78.1 %)	59 (78.7 %)	57 (67.8 %)
	NA	2 (1.6 %)	1 (2.4 %)	1 (1.3 %)	1 (1.2 %)

** Number of patients according to late distant metastatic sites (patients can exhibit different metastatic localizations): Liver (17); Lung (16); Bone (12); Brain (8); Skin (6); Other (1)

Supplementary Table 2 Clinical description of the retrospective cohorts of BC patients (Related to Figs. 2 and 8)

Two retrospective cohorts have been studied here, based either on paired (Left) and unpaired (Right) samples form PT and invaded LN. Paired cases are included in the unpaired cohort and the global LN cohort encompasses LN unpaired samples. Source data are provided as a Source Data file, as well as R scripts used to generate the figure panels.

Supplementary Table 3. Significant enriched pathways in CAF subsets from PT and LN

CAF	Category	Term	Description	LogP	Log (q-value)	nTerm InList	Symbols (part of total list)
CAF-S1 (PT)	GO Biological Processes	GO:0043062	extracellular structure	-33.33	-29.03	127/393	ABCA1, ANXA2, BMP7, CAPG, SCARB1, CD47, CDH1, COL1A1, COL1A2, COL3A1, COL4A3, COL4A4, COL5A1, COL5A2, COL6A1, COL6A2, COL6A3, COL7A1, COL8A1, COL8A2, COL10A1, COL11A1, COL12A1, COL16A1,
	Canonical Pathways	M5884	NABA CORE MATRISOME	-32.52	-28.52	102/275	AEBP1, COL1A1, COL1A2, COL3A1, COL4A3, COL4A4, COL5A1, COL5A2, COL6A1, COL6A2, COL6A3, COL7A1, COL8A1, COL8A2, COL10A1, COL11A1, COL12A1, COL15A1, COL16A1, COMP, HAPLN1, VCAN, CTGF, DCN,
	Canonical Pathways	M5885	NABA MATRISOME	-27.27	-23.58	178/753	SERPINA3, ANXA1, ANXA2, ANXA4, ANXA13, BMP4, BMP6, BMP7, CPN2, CST1, CST2, CST4, CSTA, CTSB, CTSH, CTSK, CTSL, CTSO, CTSS, MEGF6, F7, F13A1, GPC4, FGF7, FGF9, FGF10, FGF12, FGF14, FLG, GDF10,
	GO Biological Processes	GO:0001503	ossification	-18.75	-15.41	98/370	ACVR2A, ALOX15, ALPL, BMP4, BMP6, BMP7, BMPR1B, RUNX2, RUNX1, CDH11, CCR1, COL1A1, COL5A2, COL6A1, COL11A1, VCAN, CTGF, CTNNB1, CTSK, CYP27B1, ECM1, EGR2, FASN, FBN2, FGF9, FGFR2, FHL2,
	Canonical Pathways	M3468	NABA ECM REGULATORS	-16.72	-13.53	71/238	SERPINA3, CPN2, CST1, CST2, CST4, CSTA, CTSB, CTSH, CTSK, CTSL, CTSO, CTSS, F7, F13A1, LOX, LOXL1, MMP2, MMP3, MMP11, MMP12, MMP13, MMP14, MMP19, P4HA1, SERPINE1, SERPINA5, PCSK5, SERPINF1,
	GO Biological Processes	GO:0009100	glycoprotein metabolic process	-16.68	-13.53	106/444	PARP4, ARF4, BMPR1B, COL11A1, VCAN, CTNNB1, DAD1, DCN, DDOST, EXTL1, FUT8, GCNT1, B4GALT1, GOLGA2, HEXA, HEXB, IGF1, STT3A, LMAN1, MAN1A1, MAN2A1, MAN2B1, MGAT1, MGAT2, MUC1, PGM3,
	GO Biological Processes	GO:0030574	collagen catabolic process	-15.97	-12.84	34/68	COL1A1, COL1A2, COL3A1, COL4A3, COL4A4, COL5A1, COL5A2, COL6A1, COL6A2, COL6A3, COL7A1, COL8A1, COL8A2, COL10A1, COL11A1, COL12A1, COL15A1, CTSB, CTSK, CTSL, CTSS, FAP, MMP2, MMP3, MMP11,
	GO Biological Processes	GO:0042330	taxis	-13.99	-11.10	120/577	ANXA1, BMP4, BMP7, BMPR1B, BST1, C3AR1, CALR, TNFRSF8, CD74, CCR1, CCR4, ACKR2, CX3CR1, CXADR, CYP19A1, LPAR1, EDN3, EFNA4, EFNA5, EGR2, EPHB2, ETV1, F2RL1, F7, FCER1G, FGF7, FGF10, FGFR1,
	GO Biological Processes	GO:0010463	mesenchymal cell proliferation	-11.46	-8.85	24/48	BMP4, BMP7, CTNNB1, FGF7, FGF9, FGFR1, FGFR2, GPC3, PRRX1, SHOX2, SMO, STAT1, ZEB1, TGFBR2, VEGFA, WNT2, WNT5A, WNT11, DCHS1, HAND2, DCHS2, CTNNBIP1, FAT4, OSR1, G6PD, HSPA5, SMAD1, MBP,
CAF-S4 (PT)	GO Biological Processes	GO:0006936	muscle contraction	-31.16	-26.91	97/353	ACTA2, ACTC1, ACTG2, ADORA2B, ADRA1B, ADRA1A, ADRA2A, ADRA2B, GRK2, AGT, ANXA6, ATP1A2, CACNA1C, CACNA1S, CACNA2D1, CACNB2, CALCA, CALD1, CALM1, CASQ2, CAV1, CKMT2, CNN1, CRYAB,
	GO Biological Processes	GO:0030029	actin filament-based process	-27.77	-23.95	138/698	ABR, ACTC1, ACTN4, ACTN1, ADRA2A, AQP1, ARHGAP6, ARHGDIB, ATP1A2, BCL2, CACNA1C, CACNA2D1, CACNB2, CAPN3, CASQ2, CAV1, CFL2, CNN1, DES, DIAPH2, DMD, EPB41L1, EPHA3, EPS8, FAT1, HCLS1, ILK,
	KEGG Pathway	hsa04270	Vascular smooth muscle contraction	-22.37	-18.77	47/121	ACTA2, ACTG2, ADCY1, ADCY3, ADCY5, ADCY6, ADCY9, ADORA2B, ADRA1B, ADRA1A, AVPR1A, CACNA1C, CACNA1S, CALD1, CALM1, CYP4A11, EDNRA, GNA12, GUCY1A2, GUCY1A1, GUCY1B1, ITPR1, KCNMB1,
	GO Biological Processes	GO:0007264	small GTPase mediated signal	-20.33	-16.81	97/479	A2M, ABR, ADCYAP1R1, ADRA1A, ADRA2A, ARHGAP1, ARHGAP6, ARHGDIB, CDH13, CDK2, CDKN1A, CHN1, EPS8, GNA12, GNB1, GPR4, GPR20, INPP5B, ITGA3, ITPKB, NGF, NOTCH1, PAK1, PDGFRB, RAC1, RAP2A, RB1,
	Reactome Gene Sets	R-HSA-397014	Muscle contraction	-17.26	-14.04	55/206	ACTA2, ACTC1, ACTG2, ANXA6, ATP1A2, ATP1B2, ATP1B3, ATP2B1, ATP2B4, CACNA1C, CACNA1S, CACNA2D1, CACNB2, CALD1, CALM1, CAMK2G, CASQ2, DES, DMD, DMPK, FGF13, FKBP1B, ITGA1, ITPR1,
	GO Biological Processes	GO:0034329	cell junction assembly	-13.49	-10.49	49/204	ACTN4, ACTN1, AGT, ARHGAP6, BCL2, CAV1, CD151, COL17A1, EPHA3, GJA4, ILK, ITGB3, KRT5, KRT14, LAMA3, LAMA5, LAMB3, LAMC1, PTK2, PTPRK, RAB13, RAC1, ROCK1, SRC, TLN1, TNS1, VASP, VCL, CNTNAP1,
	KEGG Pathway	hsa04510	Focal adhesion	-10.88	-8.25	44/199	ACTN4, ACTN1, BCL2, CAV1, CAV2, COL4A1, COL4A2, COL4A5, IBSP, ILK, ITGA1, ITGA3, ITGA4, ITGA7, ITGB3, LAMA3, LAMA4, LAMA5, LAMB2, LAMB3, LAMC1, MYLK, PPP1R12A, PPP1R12B, PAK1, PDGFA, PDGFB,
	GO Biological Processes	GO:0030155	regulation of cell adhesion	-10.75	-8.13	94/639	ACTN4, JAG1, ANGPT1, ANGPT2, ARHGAP6, ARHGDIB, ATP5F1B, BCL2, BMP2, CAV1, CD4, CD36, CDH13, CDK6, CEBPB, CSK, DHPS, DMP1, CELSR2, EPHA2, EPHA3, ETS1, FOXF1, FOXC2, LRRC32, HLA-DPB1, HES1,
	GO Biological Processes	GO:0031032	actomyosin structure organization	-10.53	-7.96	41/181	ACTC1, ARHGAP6, CAPN3, CASQ2, CFL2, CNN1, EPB41L1, MEF2C, MYH3, MYH11, PAK1, PDGFRB, RAC1, ROCK1, SRC, TMOD1, TNFAIP1, TPM1, TTN, MKKS, CUL3, MYOM1, NRP1, MYOM2, ROCK2, CDC42BPB, WDR1, SORBS3
CAF-S1 (LN)	Canonical Pathways	M5884	NABA CORE MATRISOME	-29.43	-25.13	65/275	COL1A1, COL1A2, COL3A1, COL4A4, COL5A1, COL6A3, COL8A1, COL8A2, COL10A1, COL11A1, COL12A1, COL16A1, COMP, VCAN, DCN, DPT, EPYC, ECM1, FBLN2, FBN1, EFEMP1, FMOD, LAMA2, VWA5A, LTBP2, LUM,
	GO Biological Processes	GO:0043062	extracellular structure	-25.95	-21.95	73/393	ANXA2, CAPN2, CD34, COL1A1, COL1A2, COL3A1, COL4A4, COL5A1, COL6A3, COL8A1, COL8A2, COL10A1, COL11A1, COL12A1, COL16A1, COMP, VCAN, CYP1B1, DCN, DPT, ERCC2, FAP, FBLN2, FBN1, FMOD, B4GALT1,
	Canonical Pathways	M5885	NABA MATRISOME	-19.06	-15.46	92/753	ANXA2, ANXA3, ANXA4, BMP4, CSF3, CST1, CST2, EGF, F12, F13A1, FGF7, FGF9, FGF12, FGF14, GPC3, CXCL1, CXCL2, NRG1, IGF1, IL11, IL16, LOX, LOXL1, MMP1, MMP2, MMP3, MMP7, MMP11, MMP13, MMP16, P4HA1,
	GO Biological Processes	GO:0001503	ossification	-13.93	-10.63	53/370	ALPL, BMP4, CLTC, COL1A1, COL11A1, VCAN, ECM1, S1PR1, EGFR, EGR2, ERCC2, FASN, FGF9, GJA1, GPC3, GLI2, IGF1, LRP4, SMAD6, MMP2, MMP13, MMP16, MSX2, ROR2, OMD, ENPP1, PENK, PTGER4, PTGS2, SFRP2,
	Reactome Gene Sets	R-HSA-3781865	Diseases of glycosylation	-11.51	-8.51	28/135	VCAN, DCN, FMOD, B4GALT1, GPC3, LUM, NOTCH2, OMD, OGN, PMM2, SDC1, SDC4, THBS2, PAPSS2, ADAMTS3, ADAMTS2, GPC6, SPON1, KERA, ADAMTS5, ADAMTSL4, THSD1, GALNT12, ALG2, ADAMTSL1,
	GO Biological Processes	GO:0070848	response to growth factor	-10.54	-7.60	70/694	ANXA3, BMP4, CDH5, COL1A1, COL1A2, COL3A1, DAB2, DCN, DUSP6, EGFR, FBN1, FGF7, FGF9, FGF12, FGFR1, FMOD, FUT8, GAS1, GPC3, HAS1, HAS2, LRP4, LTBP2, LUM, SMAD6, MSX2, NCBP1, NGFR, ROR2,
	GO Biological Processes	GO:0006024	glycosaminoglycan biosynthetic	-9.90	-7.06	23/107	CLTC, VCAN, DCN, EGF, FMOD, B4GALT1, GPC3, HAS1, HAS2, LUM, OMD, OGN, SDC1, SDC4, UGDH, B4GALT6, GPC6, KERA, GALNT5, CHST15, CHPF, HS6ST2, DSEL, PAPSS2, EPYC, SPOCK1, PRG4, ASPN,
	GO Biological Processes	GO:0030335	positive regulation of cell migration	-8.50	-5.81	50/468	ANXA3, BDKRB1, BMP4, COL1A1, DAB2, S1PR1, LPAR1, EGF, EGFR, F2R, F2RL1, F3, FGF7, FGFR1, CXCL1, CXCL2, HAS2, IGF1, IL1R1, NOS3, ROR2, PDGFRA, PLAU, PODXL, RELN, PTGS2, CCL11, CXCL6, CXCL11,
	GO Biological Processes	GO:0061448	connective tissue development	-8.35	-5.69	33/242	BMP4, CD34, CHI3L1, COL1A1, COL5A1, COL11A1, COMP, EFEMP1, FGF9, FGFR1, GLI2, LUM, MMP13, MSX2, NOV, ROR2, SFRP2, TGFBR2, WNT5A, WNT10B, WNT11, WNT2B, WNT9A, WT1, ALX1, SERPINB7, FGF18,
CAF-S4 (LN)	GO Biological Processes	GO:0006936	muscle contraction	-28.62	-24.32	64/353	ACTA2, ACTC1, ACTG2, ADRA1B, ADRA1A, ADRA2B, AGT, ATP1A2, CACNA1C, CACNB2, CALD1, CASQ2, CAV1, CNN1, CRYAB, DES, DMD, DMPK, EDNRA, EDNRB, FKBP1B, GUCY1A1, HRC, ITGA1, KCNA5, KCNJ8, MYH11,
	GO Biological Processes	GO:0030029	actin filament-based process	-23.21	-19.39	82/698	ABR, ACTC1, ACTN4, ACTN1, ARHGAP6, ARHGDIB, ATP1A2, CACNA1C, CACNB2, CASQ2, CAV1, CFL2, CNN1, CSF1R, DES, DIAPH2, DMD, EPB41L1, EPS8, FAT1, GPM6B, ID1, ILK, KCNA5, KCNJ8, MEF2C, MYH11, MYL6,
	KEGG Pathway	hsa04270	Vascular smooth muscle contraction	-16.63	-13.24	29/121	ACTA2, ACTG2, ADCY5, ADRA1B, ADRA1A, AVPR1A, CACNA1C, CALD1, EDNRA, GUCY1A2, GUCY1A1, GUCY1B1, ITPR1, KCNMB1, MYH11, MYL6, MYLK, PPP1R12A, PPP1R12B, PRKCD, PRKG1, PTGIR, PLA2G4C,
	Reactome Gene Sets	R-HSA-397014	Muscle contraction	-16.57	-13.23	37/206	ACTA2, ACTC1, ACTG2, ATP1A2, ATP1B2, CACNA1C, CACNB2, CALD1, CASQ2, DES, DMD, DMPK, FKBP1B, ITGA1, ITPR1, KCNK3, MYH11, MYL6, MYLK, PLN, RYR2, SCN3A, SCN4B, SLC8A1, TLN1, TPM1, TPM2, TTN,
	GO Biological Processes	GO:0006939	smooth muscle contraction	-12.34	-9.35	23/106	ADRA1B, ADRA1A, ADRA2B, AGT, ATP1A2, CAV1, CNN1, EDNRA, EDNRB, FKBP1B, GUCY1A1, MYH11, MYLK, P2RX1, PRKG1, SMTN, SLC8A1, SSTR2, CALCRL, NMU, PLCE1, MYOCD, ARHGAP42, ABR, ANGPT1, AVPR1A,
	GO Biological Processes	GO:0030155	regulation of cell adhesion	-10.09	-7.29	55/639	ACTN4, JAG1, ANGPT1, ANGPT2, ARHGAP6, ARHGDIB, CAV1, CD3E, CD4, CD36, DHPS, DMP1, EPHA2, ETS1, FOXC2, GPM6B, HFE, HES1, IBSP, IGFBP2, ILK, ITGA3, LAMA3, LAMA5, PPP1R12A, NOTCH1, PAWR, SERPINI1,
	KEGG Pathway	hsa04510	Focal adhesion	-9.35	-6.68	27/199	ACTN4, ACTN1, CAV1, CAV2, COL4A1, COL4A2, IBSP, ILK, ITGA1, ITGA3, ITGA7, LAMA3, LAMA5, MYLK, PPP1R12A, PPP1R12B, PDGFA, PGF, PTEN, PTK2, RAC2, SPP1, TLN1, BCAR1, AKT3, MYL9, PPP1R12C,
	GO Biological Processes	GO:0001568	blood vessel development	-9.10	-6.45	57/718	ACTA2, ACVR2B, JAG1, AGT, ANGPT1, ANGPT2, ADGRB3, PRDM1, CAV1, COL4A1, COL4A2, CSPG4, EDNRA, EPHA2, ENPEP, EPAS1, ETS1, FGF1, FOXC2, FOXS1, GJA4, GPR4, HES1, ID1, LAMA5, MCAM, MEF2C, MFGE8,
	GO Biological Processes	GO:0030048	actin filament-based movement	-8.67	-6.13	21/132	ACTC1, ACTN4, ATP1A2, CACNA1C, CACNB2, CAV1, DES, DMD, KCNA5, KCNJ8, MYL6, PLN, RYR2, SCN4B, SNTA1, TPM1, TPM2, TTN, GJC1, SYNE2, CTNNA3, ADRA1A, ATP1B2, CACNA1A, CASQ2, CBLN1, DMPK,
CAF-S1 (common to PT and LN)	Canonical Pathways	M5884	NABA CORE MATRISOME	-34.94	-30.63	59/275	COL1A1, COL1A2, COL3A1, COL4A4, COL5A1, COL6A3, COL8A1, COL8A2, COL10A1, COL11A1, COL12A1, COL16A1, COMP, VCAN, DCN, DPT, EPYC, ECM1, FBLN2, FBN1, EFEMP1, FMOD, LAMA2, LTBP2, LUM, MFAP2,
	Canonical Pathways	M5885	NABA MATRISOME	-18.01	-14.48	69/753	ANXA2, ANXA4, BMP4, CST1, CST2, F13A1, FGF7, FGF9, FGF12, FGF14, GPC3, CXCL1, CXCL2, NRG1, IGF1, IL11, IL16, LOX, LOXL1, MMP2, MMP3, MMP11, MMP13, P4HA1, PCSK5, SERPINE2, PLAU, PLXNB3, CCL11,
	GO Biological Processes	GO:0030199	collagen fibril organization	-12.79	-9.59	16/50	ANXA2, COL1A1, COL1A2, COL3A1, COL5A1, COL11A1, COL12A1, CYP1B1, DPT, FMOD, LOX, LUM, MMP11, P4HA1, SFRP2, TNXB, COL4A4, COL6A3, COL8A1, COL8A2, COL10A1, COL16A1, LOXL1, MMP3, MMP13, P4HB,
	GO Biological Processes	GO:0001501	skeletal system development	-12.60	-9.44	47/505	ALPL, ANXA2, BMP4, CHI3L1, COL1A1, COL1A2, COL3A1, COL6A3, COL10A1, COL11A1, COL12A1, COMP, VCAN, FBN1, EFEMP1, FGF9, FGFR1, GLI2, HAS2, IGF1, INSIG1, LUM, MMP2, MMP13, NOTCH2, NOV, NPR3,
	GO Biological Processes	GO:0001503	ossification	-11.32	-8.29	38/377	ALPL, BMP4, COL1A1, COL11A1, VCAN, ECM1, EGR2, FASN, FGF9, GJA1, GPC3, GLI2, IGF1, LRP4, MMP2, MMP13, ROR2, OMD, ENPP1, PENK, PTGS2, SFRP2, SOX11, TEK, TUFT1, WNT5A, WNT10B, WNT11, MRC2,
	GO Biological Processes	GO:0006024	glycosaminoglycan biosynthetic	-11.08	-8.07	20/106	VCAN, DCN, FMOD, B4GALT1, GPC3, HAS1, HAS2, LUM, OMD, OGN, SDC1, SDC4, UGDH, B4GALT6, KERA, GALNT5, CHST15, CHPF, HS6ST2, DSEL, EPYC, SPOCK1, PRG4, ASPN, PODN, PAPSS2, CHI3L1, PIM1, ADCY2,
	GO Biological Processes	GO:0070848	response to growth factor	-10.63	-7.71	54/722	BMP4, CDH5, COL1A1, COL1A2, COL3A1, DAB2, DCN, DUSP6, FBN1, FGF7, FGF9, FGF12, FGFR1, FMOD, FUT8, GAS1, GPC3, HAS1, HAS2, LRP4, LTBP2, LUM, ROR2, P2RY1, PDGFRA, PENK, PXN, SFRP2, SFRP4, SKIL,
	GO Biological Processes	GO:0097435	supramolecular fiber organization	-9.16	-6.43	48/657	ADD2, ADD3, ANXA2, COL1A1, COL1A2, COL3A1, COL5A1, COL11A1, COL12A1, CYP1B1, DPT, DPYSL3, LPAR1, F2RL1, FMOD, LOX, LOXL1, LTBP2, LUM, MFAP4, MID1, MMP11, P4HA1, PDGFRA, PXN, CCL11, SDC4, SFRP2,
	GO Biological Processes	GO:0032963	collagen metabolic process	-8.64	-6.03	17/102	BMP4, COL1A1, COL5A1, F2R, FAP, MFAP4, MMP2, MMP3, MMP11, MMP13, MYB, TNXB, SERPINB7, ADAM15, MRC2, RGCC, CREB3L1
CAF-S4 (common to PT and LN)	GO Biological Processes	GO:0006936	muscle contraction	-35.61	-31.30	63/349	ACTA2, ACTC1, ACTG2, ADRA1B, ADRA1A, ADRA2B, AGT, ATP1A2, CACNA1C, CACNB2, CALD1, CASQ2, CAV1, CNN1, CRYAB, DES, DMD, DMPK, EDNRA, EDNRB, FKBP1B, GUCY1A1, HRC, ITGA1, KCNA5, KCNJ8, MYH11,
	GO Biological Processes	GO:0030029	actin filament-based process	-25.92	-22.08	75/724	ABR, ACTC1, ACTN4, ACTN1, ARHGAP6, ARHGDIB, ATP1A2, CACNA1C, CACNB2, CASQ2, CAV1, CFL2, CNN1, DES, DIAPH2, DMD, EPB41L1, EPS8, FAT1, HIP1, ILK, KCNA5, KCNJ8, MEF2C, MYH11, MYL6, PAWR, PDGFA,
	KEGG Pathway	hsa04270	Vascular smooth muscle contraction	-20.16	-16.70	29/121	ACTA2, ACTG2, ADCY5, ADRA1B, ADRA1A, AVPR1A, CACNA1C, CALD1, EDNRA, GUCY1A2, GUCY1A1, GUCY1B1, ITPR1, KCNMB1, MYH11, MYL6, MYLK, PPP1R12A, PPP1R12B, PRKCD, PRKG1, PTGIR, PLA2G4C,
	Reactome Gene Sets	R-HSA-397014	Muscle contraction	-19.94	-16.53	36/206	ACTA2, ACTC1, ACTG2, ATP1A2, ATP1B2, CACNA1C, CACNB2, CALD1, CASQ2, DES, DMD, DMPK, FKBP1B, ITGA1, ITPR1, KCNK3, MYH11, MYL6, MYLK, PLN, RYR2, SCN3A, SCN4B, SLC8A1, TLN1, TPM1, TPM2, TTN,
	GO Biological Processes	GO:0006939	smooth muscle contraction	-16.27	-13.19	24/105	ACTA2, ADRA1B, ADRA1A, ADRA2B, AGT, ATP1A2, CAV1, CNN1, EDNRA, EDNRB, FKBP1B, GUCY1A1, MYH11, MYLK, P2RX1, PRKG1, SMTN, SLC8A1, SSTR2, CALCRL, NMU, PLCE1, MYOCD, ARHGAP42, ABR, ANGPT1,
	GO Biological Processes	GO:0001568	blood vessel development	-11.28	-8.52	53/756	ACTA2, JAG1, AGT, ANGPT1, ANGPT2, ADGRB3, CAV1, COL4A1, COL4A2, CSPG4, EDNRA, EPHA2, ENPEP, EPAS1, ETS1, FOXC2, FOXS1, GJA4, GPR4, HES1, LAMA5, MCAM, MEF2C, MFGE8, MYLK, NOTCH1, NOTCH3,
	GO Biological Processes	GO:0030048	actin filament-based movement	-11.06	-8.31	21/132	ACTC1, ACTN4, ATP1A2, CACNA1C, CACNB2, CAV1, DES, DMD, KCNA5, KCNJ8, MYL6, PLN, RYR2, SCN4B, SNTA1, TPM1, TPM2, TTN, GJC1, SYNE2, CTNNA3, ADRA1A, FKBP1B, P2RX1, SCN3A, SLC8A1, CACNA1H,
	KEGG Pathway	hsa04510	Focal adhesion	-9.16	-6.74	23/199	ACTN4, ACTN1, CAV1, CAV2, COL4A1, COL4A2, IBSP, ILK, ITGA1, ITGA3, ITGA7, LAMA3, LAMA5, MYLK, PPP1R12A, PPP1R12B, PDGFA, PGF, PTK2, TLN1, BCAR1, MYL9, PPP1R12C, CFL2, DIAPH2, PIP5K1B,
	GO Biological Processes	GO:0030155	regulation of cell adhesion	-9.02	-6.62	44/647	ACTN4, JAG1, ANGPT1, ANGPT2, ARHGAP6, ARHGDIB, CAV1, CD4, CD36, DHPS, DMP1, EPHA2, ETS1, FOXC2, HES1, IBSP, IGFBP2, ILK, ITGA3, LAMA3, LAMA5, PPP1R12A, NOTCH1, PAWR, SERPINI1, PRKCD, PRKG1,

Supplementary Table 3 Significant enriched pathways in CAF subsets from PT and LN (Related to Fig. 3)

Pathways are defined using Metascape gene annotation resource according to the up-regulated genes in CAF-S1 *versus* CAF-S4, and in CAF-S4 *versus* CAF-S1 when isolated from PT (1st and 2nd tabs) or from LN (3rd and 4th tabs). 5th (CAF-S1) and 6th (CAF-S4) tabs show pathways and corresponding genes, which are common in PT and LN. Source data are provided as a Source Data file, as well as R scripts used to generate the figure panels.

Supplementary Table 4. Top differential genes between CAF-S1 and CAF-S4

TOP 250 GENES UP CAF-S1 (PT+LN) [total n = 2670 genes]				
DAB2	CHPF	EFEMP1	ADC	WNT5A
LRRC15	ENPP1	P4HB	HOOK1	CALU
FBN1	SHISA2	HSD17B6	BZW1	SAMD11
TSKU	CCL11	CILP2	CRABP2	PCSK5
COL11A1	FRMD6	CHRDL1	RSAD2	CHRNA1
CTHRC1	COL3A1	GJA1	EGR2	CLMP
MMP13	TMEM119	TPST1	WT1	MPP3
PTGFRN	CGREF1	CYP11A1	ISLR	SERPINB7
THBS2	C3	GPR133	LOC100288077	LOC100192378
MXRA5	WNT2	DEPDC7	CPXM2	CCDC109B
COMP	GFPT2	GPX8	SH3D19	FAM3C
EMP1	COPZ2	COLEC12	BNC2	PI16
ODZ4	NHSL1	KDELR3	ARL1	CPZ
LUM	VCAN	LXN	OGN	DPYSL3
MORC4	MFAP5	BMP4	LSP1	CST2
COL10A1	PODN	KIAA0930	FGFR1	NEK6
GAS7	SERINC2	SEMA3D	ARHGAP20	GRIA3
MMP2	DPEP1	CYBRD1	MEGF10	C13orf15
EPYC	LOC283867	NRK	IL16	LOC100507632
PTGIS	GPR1	TSPAN18	LOC100129034	B4GALT1
CST1	CNIH3	PLXDC2	IL20RA	F2RL1
SPOCK1	ITGA11	ADAM12	PLEKHA6	BOC
MMP3	SURF4	HSPA12B	DEPTOR	COL12A1
LOX	SFRP2	KCNJ6	TTC3	FAIM2
VDR	GLT8D2	TMED10	PRRG3	ALPL
PPAPDC1A	LMCD1	UGDH	PLAUR	SEMA3C
DIO2	RPN1	TSHZ3	GJB2	ARSI
SYNDIG1	SFRP4	LOXL1	DMGDH	IL11
EPB41L3	UAP1	MEG3	FAM155A	STMN2
CCDC80	CERCAM	XPNPEP2	TLR1	PRLR
MOXD1	SPON1	FAP	NKD2	SRPX
MXRA8	IGJ	IGFL2	LOC100134229	FAM114A1
KLHL4	DCN	PENK	PMP22	FSTL1
PDGFRA	KCNK2	DRP2	OMD	DRAM1
FNDC1	COL1A1	ADD2	MME	SLC16A14
PLAU	MFSD2A	COL8A1	TNFRSF8	PPIC
TNFSF4	BCL6B	CELF2	S100A16	CDH5
CYP1B1	MRC2	P4HA1	FBLN5	CDSN
SLC20A1	CILP	SKIL	SLC12A8	HS6ST2
KERA	C1QTNF3	PYCR1	FLNB	CD55
COL1A2	ASAP3	IGF1	SDC1	SFT2D2
MPZL1	MFAP2	XG	TSHZ2	KCNK1
F13A1	SCN7A	STEAP2	AGTRAP	CNGA3
LTBP2	KDELR2	GRINA	ELTD1	TTC39C
PDGFRL	TEK	COL16A1	CXCL11	SULF2
C1S	NUCB2	SLC41A2	MMP11	LINC00574
ALDH1A3	PRICKLE1	HMCN1	F2RL2	RALGPS2
LRP1	SLC1A5	SCML2	ABCA12	BDKRB2
RTN4	WNT2B	IRX6	ZCCHC5	EXPH5
COL6A3	KLK4	CMYA5	EHD4	ASPN

TOP 250 GENES UP CAF-S4 (PT+LN) [total n = 1825 genes]				
C21orf7	TBX2	RBPMS2	EGFL6	SEPT7
MCAM	PDE3A	KCNK3	SPRY4	NCK2
MEF2C	SLC38A11	CSPG4	LZTS1	PLA2G4C
MOCS1	TINAGL1	COL18A1	FAM13C	IL17B
PTP4A3	ATP1A2	CSRP2	SMTN	ARHGDIB
NRIP2	ARHGAP44	SEPT4	FOXS1	ECEL1
MYH11	CABP1	MAP2	ATP8B1	CLMN
PARM1	GPR4	WFDC1	KCNJ8	SORBS3
CDH6	OAZ2	ANGPT1	DOCK10	RAB15
GJA4	RASD2	PCDH1	FRMD4A	COL4A2
CACNB2	TRPC4	CACNA1H	SH2D3C	GRIN2C
OR51E1	KCNA5	PPARGC1B	CHN1	CHST2
LINGO1	FADS3	CRYAB	FABP4	SSTR2
ESAM	GPR116	CTNNA3	PI15	C1QTNF1
NDUFA4L2	SCN4B	EEF1DP3	PHLDA1	KIAA0040
PPP1R14A	FAM162B	CPM	EGFLAM	LOC400456
CASQ2	KCNMB1	ANGPT2	CRTC3	SORT1
COX4I2	JPH2	GMFG	NDRG2	DACT3
ABTB1	KIAA1274	RCAN2	ITGA1	PYGM
ACAN	LGALSL	KIAA1751	SPEG	CDK18
MICAL1	LRRC10B	SEPT7P2	CCDC3	PDE1A
ENPEP	CD4	HEYL	PELI1	RAB6B
FHL5	OR51E2	LYPD1	OLFML2A	NR2F2
LDB3	HEY2	FRMD3	CALCRL	DMD
MYOCD	SPTB	MAPRE2	P2RX1	SYNE2
DGKG	HIGD1B	LBH	DLX5	UBA2
ADAP2	ABCC9	C3orf70	GRM8	GUCY1A3
SEMA5B	PPP1R12B	EPAS1	SCN3A	PRR16
ARHGEF17	GABRD	FBLIM1	ARHGAP10	MIR143HG
GPR20	ATP1B2	LAMA5	LGR6	CNN1
RGS5	ZAK	RASL11A	RASL12	CD93
ACTC1	RBPMS	PTPLA	TESC	AVPR1A
ITGA7	PGF	DMPK	LOC100506035	BAI3
PLN	PPP1R12A	DAAM2	ACTA2	MTHFD2
RCSD1	A2M	RGS6	KLHL30	NMNAT2
LGI4	APOLD1	EPS8	SNTA1	CAV2
TRPC6	AGAP11	PLEKHH3	PVRL1	LOC100507463
DPY19L2	AKAP6	PRPH	POPDC2	CACNA1A
REM1	SUSD5	ITIH4	SLC8A1	ARHGEF7
RERGL	PDGFA	CCDC102B	LOC100131347	ANGPT4
SLC35F1	AOC3	FOXC2	WNT6	FAM212B
GJC1	ARID5A	HRH2	MYLK	GRIN2A
NFASC	P2RY14	SLCO1C1	ISYNA1	CD36
NRARP	HTR1F	ASB2	COL4A1	PDE4C
SOX5	SERPINI1	HIP1	CRIM1	ARHGAP42
KCNK17	CBFA2T3	ARHGAP26	CSRNP3	FILIP1
RASGRP2	CCR10	PTK2	GUCY1A2	CAV1
NEURL1B	HRC	UTRN	ITGA3	NRGN
TMEM74B	NOTCH3	ACTG2	TMEM136	ARHGAP6
DGKB	MYOM1	PLXND1	SLC2A4	HDAC5

Supplementary Table 4 Top differential genes between CAF-S1 and CAF-S4 (Related to Fig. 3)

Paired differential analyses were performed between CAF-S1 and CAF-S4, considering all human breast fresh samples from PT and LN (n = 10 pairs). The 250 most up-regulated genes in each CAF subsets are shown in the table (left part, in CAF-S1; right part, in CAF-S4). Genes are ranked by column, from left to right, starting from the most differential gene. Source data are provided as a Source Data file, as well as R scripts used to generate the figure panels.

Supplementary Table 5. List of differential genes between PT and LN in CAF-S1 or in CAF-S4

CAF-S1 UP PT (n = 164)			CAF-S1 UP LN (n = 147)			CAF-S4 UP PT (n = 24)	CAF-S4 UP LN (n = 42)
HSD17B6	CALB2	PIK3AP1	DMRT3	KLHL13	CLU	ROR2	CILP
INHBA	FIBIN	NUAK1	MATN4	RP1-177G6.2	C1QTNF2	PAX3	SFRP4
GRP	RFX8	PCDH7	DACT2	MYPN	SFRP4	KRT14	RAMP3
HSD17B14	ST8SIA4	TAGLN	KCNK5	CACNB4	TRPV3	KRT5	L1CAM
MRVI1	TMEM130	LEF1	LAMA3	FAM70A	CCBP2	MMP16	DACT2
HPSE2	KIF26B	ACTN1	SEMA3C	MYO5B	RSPO2	CHI3L1	TNFSF13B
EGFL6	CAPN6	TMEM204	AIF1L	XPNPEP2	EFNA5	KRT23	PI16
RSPO1	MUC6	DERL3	TPPP	PCSK6	NOV	OXTR	CP
NRN1	GBP5	VSNL1	THBD	ANXA3	DSCAML1	WDR67	TMEM150C
LINC00340	ACTA2	POSTN	GPR64	PRG4	LOC100130705	TRIM29	MYOC
PRDM1	C9orf167	ALDH1A2	MFAP5	MLIP	ANGPTL1	KLK5	TMEM176B
CNIH3	FHAD1	KRT17	CAB39L	TRERF1	CEP152	GPC6	IL7R
LAMP5	RIMBP2	NTF3	WNT10B	PLA2G4A	HAS1	CCDC165	GREM1
LMOD1	SLC7A7	GPC4	SPRR2F	EFEMP1	SH2D4A	KRT6B	PCOLCE2
MITF	LAYN	RUNX3	FNDC5	FABP3	GADD45G	JAG1	MFAP5
SYNDIG1	CTSK	PLCB4	CADM3	FAM180B	HLA-DRB1	MMP7	ADAM28
SGIP1	FAM180A	CDH11	PDE1C	PAMR1	CCDC102B	KRT17	C6
SEPT4	IGFBP3	MPPED2	KCNS2	LOC100507632	PTHLH	EDIL3	SCN3B
GJA5	TMEM200A	IGFL2	KIRREL3	CXCR7	C4orf34	IRX5	SCARA5
COL10A1	COL11A1	PAWR	SGCG	EBF2	AGXT2L1	C10orf12	TMEM154
SIM1	TRIM59	SERPINA11	DOCK8	SCRG1	CLEC3A	SYT8	CXCL13
MMP11	GREM1	DYNC1I1	PCOLCE2	SSH2	FANCI	ZDHHC2	PLA2G2A
NXN	HS3ST3A1	RBP1	ABCC2	WSCD2	UAP1	VWA3B	GP1BA
CDC42EP3	MYH2	LRRC15	STXBP6	TMEM233	ADAMTSL4	GRIK3	HMGCS2
MYH13	FBXO32	LOC283867	NFKBIZ	FAM5C	RAD51		CYP1B1
MYH4	FAM101B	LOC100499467	MSTN	CREB5	SIGLEC1		TEX264
CTHRC1	MDK	NRP2	KIAA1908	SLPI	HSPB8		SLC39A7
CORIN	DLL1	HS3ST3B1	ITGB3	C7			HN1
MOB3B	PTK7	PCDHB10	TGM5	C6			CENPW
F2RL2	ATP6V0D2	MMP28	TSPAN8	SPTBN1			KCNT2
PGR	WHAMMP2	IRS2	SCARA5	CD44			NBEAL2
KRT23	BMPR1B	OLFM2	KRT222	ABLIM1			CYBA
TPM2	FGF1	DTX4	FHDC1	PROCR			CPZ
LOC100506013	WFDC1	SPECC1	PXDNL	LOC100505633			TMEM176A
CNN2	SDC1	SCG5	GPR133	CIT			SFRP2
ARSE	KCNS3	DCBLD1	EPHA3	DMRT2			ARHGEF3
C11orf41	DPEP1	ISL1	GGT1	ADAMTS5			CDRT1
MIAT	SEPT6	NKX3-2	PLA2G5	C9orf66			CLDN5
NKD2	CADM1	WNT5B	GRK5	ARHGAP29			HAS2
FAM176A	JPH2	SUSD3	ADD2	KIF11			MATL2963
ALDH1B1	PCSK1N	LGALS9	SDK1	FGD5			IGFBP6
EPHX3	RUNX2	FOXQ1	LBP	EMILIN2			MAPK13
GSTT2	ERMN	ITGB2	TIAM1	LURAP1L			
EDNRA	MYLK	AEBP1	NFIB	CP			
PRKG1	IQCA1		TPPP3	PIK3C2B			
DCX	GPC1		ACE	CD70			
NPTX2	GAD1		PLEKHG3	USP53			
KLK4	FGFR2		GRIA4	AOX1			
CRABP1	DCLK2		KLHL4	AKR1C3			
GLIS1	SCUBE2		DKK1	MYOC			
SPHK1	PDE1B		EPB41L3	PTGS1			
CHST11	C11orf93		MGLL	NDFIP2			
RAB31	ETV1		GPSM2	SDPR			
PODNL1	ROR2		LGR4	C13orf33			
ZMAT4	PPP1R13L		LOC283624	ARHGAP15			
LOC100288077	MFAP2		CYSLTR1	ANGPTL5			
PRR16	CENPV		MBNL3	FAM117B			
UNC5B	MAGEL2		ACE2	SEMA3B			
CA9	C11orf92		IMPA2	AKR1C2			
KIAA1462	FADS1		HLA-DRA	PKP2			

Supplementary Table 5 List of differential genes between PT and LN in CAF-S1 or in CAF-S4 (Related to Fig. 3)

Paired differential analyses were performed between CAF-S1 from PT and from LN (n = 5 pairs, left part of the table) and between CAF-S4 from PT and from LN (n = 5 pairs, right part of the table). All differential genes between PT and LN in CAF-S1 or in CAF-S4 are shown in the table and are ranked by column, from left to right, starting from the most differential gene. Source data are provided as a Source Data file, as well as R scripts used to generate the figure panels.

Supplementary Table 6. Univariate and Multivariate Cox regression analyses for progression-free survival and overall survival in N+ breast cancer patients considering 60 cases with high stromal percentage (LN cohort)

		Disease-free survival						Overall survival					
		Univariate			Multivariate			Univariate			Multivariate		
		Hazard ratio	95% CI	p value	Hazard ratio	95% CI	p value	Hazard ratio	95% CI	p value	Hazard ratio	95% CI	p value
LN CAF enrichment	CAF-S1 (reference)	1	-	-	1	-	-	1	-	-	1	-	-
	CAF-S4	1.6	0.6-4.1	0.3	2.5	0.8-7.2	0.1	2.7	0.9-8	0.08	5.3	1.5-18	0.008
LN status at diagnosis	N1 (reference)	1	-	-	1	-	-	1	-	-	1	-	-
	N2	3.6	1.1-11.7	0.03	7	1.9-25.7	0.003	4.3	1.2-15.8	0.03	7.8	1.8-33.4	0.005
	N3	6.8	2-23.4	0.002	13	3.4-50	0.0002	7.9	2-30.6	0.003	14.9	3.3-66.5	0.0004
BC subtype	Lum A (reference)	1	-	-	1	-	-	1	-	-	1	-	-
	Lum B	0.7	0.2-2.7	0.6	0.4	0.1-1.6	0.2	1.7	0.3-8.6	0.5	0.8	0.2-4.1	0.8
	HER2	0.7	0.2-3.3	0.7	1.2	0.2-6	0.8	1.4	0.2-8.3	0.7	2.1	0.3-13.8	0.4
	TN	3.4	1-11.6	0.05	6.7	1.7-26.5	0.006	6.1	1.3-29	0.02	11.5	2.2-60.8	0.004

CI = confidence interval

Supplementary Table 6 Cox regression in N+ BC patients with high stromal content (Related to Fig. 8)

Univariate and Multivariate Cox regression analyses using additive hazards models for disease-free survival and overall survival in N+ BC patients with high stromal content (N = 60). LN stromal quantity was defined as percentage of stroma relative to epithelial compartment in LN sections (n = 119). Patient subgroup was defined according to the median. Source data are provided as a Source Data file, as well as R scripts used to generate the figure panels.

Supplementary Movie 1 CAF-S1 spheroid embedded in collagen (Related to Figure 4) Length of the video is 42h. Pictures were taken every 15 minutes, video is mounted with 8 frames per second. Size of the frame: 2 x 2.4 mm.

Supplementary Movie 2 CAF-S4 spheroid embedded in collagen (Related to Figure 4) Length of the video is 42h. Pictures were taken every 15 minutes, video is mounted with 8 frames per second. Size of the frame: 2 x 2.4 mm.
