# Supplementary material for: Cancer-associated fibroblast heterogeneity in axillary lymph nodes drives metastases in breast cancer through complementary mechanisms
Source: Nat Commun. 2020 Jan 21;11:404. doi: 10.1038/s41467-019-14134-w (PMC6972713; doi:10.1038/s41467-019-14134-w)
Supplement: Supplementary file 3 — Additional Supplementary Files [file 41467_2019_14134_MOESM3_ESM.docx]

**Description of Additional Supplementary Files**

**Supplementary Movie 1:** CAF-S1 spheroid embedded in collagen (Related to Figure 4) Length of the video is 42h. Pictures were taken every 15 minutes, video is mounted with 8 frames per second. Size of the frame: 2 x 2.4 mm.

**Supplementary Movie 2:** CAF-S4 spheroid embedded in collagen (Related to Figure 4) Length of the video is 42h. Pictures were taken every 15 minutes, video is mounted with 8 frames per second. Size of the frame: 2 x 2.4 mm.
