# Supplementary material for: Cancer-associated fibroblast heterogeneity in axillary lymph nodes drives metastases in breast cancer through complementary mechanisms
Source: Nat Commun. 2020 Jan 21;11:404. doi: 10.1038/s41467-019-14134-w (PMC6972713; doi:10.1038/s41467-019-14134-w)
Supplement: Supplementary file 6 — Reporting Summary [file 41467_2019_14134_MOESM6_ESM.pdf]

nature research

Corresponding author(s): MECHTA-GRIGORIOU Fatima

Last updated by author(s): Sep 27, 2019

Reporting Summary

Nature Research wishes to improve the reproducibility of the work that we publish. This form provides structure for consistency and transparency in reporting. For further information on Nature Research policies, see Authors & Referees and the Editorial Policy Checklist .

Statistics

For all statistical analyses, confirm that the following items are present in the figure legend, table legend, main text, or Methods section.

n/a	Confirmed	
☐	☒	The exact sample size (*n*) for each experimental group/condition, given as a discrete number and unit of measurement
☐	☒	A statement on whether measurements were taken from distinct samples or whether the same sample was measured repeatedly
☐	☒	The statistical test(s) used AND whether they are one- or two-sided<br>*Only common tests should be described solely by name; describe more complex techniques in the Methods section.*
☐	☒	A description of all covariates tested
☐	☒	A description of any assumptions or corrections, such as tests of normality and adjustment for multiple comparisons
☐	☒	A full description of the statistical parameters including central tendency (e.g. means) or other basic estimates (e.g. regression coefficient) AND variation (e.g. standard deviation) or associated estimates of uncertainty (e.g. confidence intervals)
☐	☒	For null hypothesis testing, the test statistic (e.g. *F*, *t*, *r*) with confidence intervals, effect sizes, degrees of freedom and *P* value noted<br>*Give P values as exact values whenever suitable.*
☒	☐	For Bayesian analysis, information on the choice of priors and Markov chain Monte Carlo settings
☒	☐	For hierarchical and complex designs, identification of the appropriate level for tests and full reporting of outcomes
☒	☐	Estimates of effect sizes (e.g. Cohen's *d*, Pearson's *r*), indicating how they were calculated

Our web collection on statistics for biologists contains articles on many of the points above.

Software and code

Policy information about availability of computer code

Data collection	No software was used to collect the data
Data analysis	Software used for the analyses are: R (https://cran.r-project.org, R versions 3.3.1, 3.4.0 and 3.5.0), Fiji (ImageJ v2.0.0-rc-19/1.49m, v2.0.0-rc-65/1.52b), IMARIS (v8.4.1), Matlab. Source data are provided as a Source Data file, as well as R scripts used to generate the figure panels.

For manuscripts utilizing custom algorithms or software that are central to the research but not yet described in published literature, software must be made available to editors/reviewers. We strongly encourage code deposition in a community repository (e.g. GitHub). See the Nature Research guidelines for submitting code & software for further information.

Data

Policy information about availability of data

All manuscripts must include a data availability statement. This statement should provide the following information, where applicable:

- Accession codes, unique identifiers, or web links for publicly available datasets
- A list of figures that have associated raw data
- A description of any restrictions on data availability

RNAseq data from EPCAM+, CAF-S1 and CAF-S4 sorted from breast cancer samples, without or with culture, generated in this study are available on European Genome-Phenome Archive platform (https://ega-archive.org) under accession number: EGAS00001003238

Field-specific reporting

Please select the one below that is the best fit for your research. If you are not sure, read the appropriate sections before making your selection.

Life sciences study design

All studies must disclose on these points even when the disclosure is negative.

Sample size	No sample-size calculation was performed
Data exclusions	No data was excluded from the analysis
Replication	The number of independent experiments is specified in each figure legend, with at least 3 independent experiments, unless otherwise specified.
Randomization	No method of randomization was used.
Blinding	In the prospective cohort, we collected all available tumor biopsies blindly, as patient information was not accessible at time of surgery. Method for sample collection is indicated p 23-24 of the main manuscript.

Reporting for specific materials, systems and methods

We require information from authors about some types of materials, experimental systems and methods used in many studies. Here, indicate whether each material, system or method listed is relevant to your study. If you are not sure if a list item applies to your research, read the appropriate section before selecting a response.

Materials & experimental systems

n/a	Involved in the study	
☐	☒	Antibodies
☐	☒	Eukaryotic cell lines
☒	☐	Palaeontology
☒	☐	Animals and other organisms
☐	☒	Human research participants
☐	☒	Clinical data

Methods

n/a	Involved in the study	
☒	☐	ChIP-seq
☐	☒	Flow cytometry
☒	☐	MRI-based neuroimaging

Antibodies

Antibodies used

Flow cytometry antibodies:
anti-EpCAM-BV605 (Dilution 1:50, BioLegend, # 324224, clone 9C4, lot B251369)
anti-CD31-PECy7 (Dilution 1:100, BioLegend, #303118, clone WM59, lot B190791)
anti-CD45-APC-Cy7 (Dilution 1:20, BD Biosciences, #BD-557833, lot 7061521)
anti-CD235a-PerCP/Cy5.5 (Dilution 1:50, Biolegend, #349109, clone H1264, lot B22905)
anti-CD29-Alexa Fluor 700 (Dilution 1:100, BioLegend, #303020, clone TS2/16, lot B191180)
anti-FAP-APC (primary antibody, dilution 1:200, R&D Systems, #MAB3715, clone 427819, lot CCHZ0215051)
anti-PDGFRβ-PE (Dilution 1:40, BioLegend, #323606, clone 18A2, lot B178266)
anti-PDPN-Alexa Fluor 488 (Dilution 1:50, BioLegend, #337006, clone NC-08, lot B-272519)
anti-SMA-Alexa Fluor 594 (Dilution 1:25, R&D Systems, #IC1420T-025, lot AEMXO1 18021)
iso-anti-CD29 (Dilution 1:100, BioLegend, #400144, clone MOPC-21, lot B239102)
iso-anti-FAP (primary antibody,dilution 1:200, R&D Systems, #MAB002, clone11711, lot IX2416081)
iso-anti-PDGFRβ (Dilution 1:20, BioLegend, #400114, clone MOPC-21, lot B2020...)
iso-anti-PDPN (Dilution 1:125,BioLegend, #400525, clone RTK758, lot B228070)
iso-anti-SMA (Dilution 1:25,R&D Systems, #IC003T, lot AELEO114121)

Immunohistochemistry antibodies:
anti-FAP (pH6, 1h at 1:200, Vitatex #MABS1001, Clone D8, lot A-001)
anti-CD29 (pH6, 1h at 1:100, Abcam #ab3167, clone 4B7R, lot GR166283-2)
anti-FSP1 (pH6, 1h at 1:250, Abcam #ab27957, lot GR208945-1)
anti-αSMA (pH6, 30min at 1:200, Dako #M0851, clone 1A4, lot 20025132)
anti- PDGFRβ (pH9, 1h at 1:100, Abcam #ab32570, lot GR144633-4)
anti-EPCAM (pH6, 1h at 1:200, Dako #M0804, cloneBer-EP4, lot 20020071)
anti-MCAM (pH9, 1h at 1/1000, Sigma #HPA008848, lot A61467).

Immunofluorescence antibodies:
anti-Vinculin (1:1000, Sigma, #V9131, lot 018M4779V)
E-Cadherin (1:300, Cell Signaling Technology, #3195, 24E10, lot 13)
Cy3-anti-mouse secondary (1:500, JacksonImmunoResearch, #715-165-150)

Cy3-anti-rabbit secondary (1:500, JacksonImmunoResearch, # 711-165-152, lot 136 182)
Alexa Fluor TM 488 phalloidin (1:200, Invitrogen, #A12379, lot 1885245)

Validation	For multicolor flow cytometry, antibodies were validated by titration, using cell lines expressing the proteins of interest. Data were compared to those obtained with the corresponding isotype controls. For IHC, antibody specificity was validated using a strategy of silencing of the protein of interest in cell lines. After silencing, the antibody was tested by using western blot and IHC in the exact same conditions as those used for patient samples (FFPE-embedded cell lines +/- siRNA). We also used an isotype control for IHC. The antibodies used for IF were first defined according to the literature and validated according to the expected localization of the detected signal in cell lines.

Eukaryotic cell lines

Policy information about cell lines

Cell line source(s)	MCF7 and T47D luminal breast cancer cells are from the lab bank (Institut Curie). T47D-GFP cells are from CellBiolabs (AKR-208). MCF7-GFP and MDA-231-GFP are home-made.
Authentication	For all cell lines used in the manuscript, cell line identity was verified by using the Short Tandem Repeat (STR) DNA profiling (Promega # B9510) method.
Mycoplasma contamination	All cells lines used were negative for mycoplasma. Cell lines have been tested by using the Mycoplasma Detection kit for conventionnal PCR (MB minerva biolabs #11-1250);
Commonly misidentified lines (See ICLAC register)	No misidentified cell line was used in the study

Human research participants

Policy information about studies involving human research participants

Population characteristics	Detailed descriptions of prospective and retrospective cohorts of breast cancer patients are available in Tables S1 and S2.
Recruitment	Prospective cohorts: Breast cancer primary tumors and invaded metastatic lymph nodes were included prospectively in our study. All fresh samples were collected by a referent pathologist. The surgical residues, available after histopathological analyses and used in our manuscript, were not required for diagnosis. There was no interference with clinical practice.<br><br>Retrospective cohorts: Primary tumor and lymph node samples from breast cancer patients, corresponding to invasive breast cancers with at least one metastatic lymph node at diagnosis, have been included and used for IHC analyses. Here again, there was no interference with clinical practice. Retrospective cohorts include Luminal, HER2 and TN subtypes. IHC were performed on residual surgery samples prior to any treatment (i.e. prior to radiation, hormonal or chemo-therapy).<br><br>Clinical features of prospective and retrospective cohorts are listed in Tables S1 and S2, respectively.
Ethics oversight	All patients (in both prospective and retrospective cohorts) included in our study were informed by their referring oncologist that their biological samples could be used for research purposes and they gave their verbal informed consent. In case of patient refusal, that could be either orally expressed or written, residual tumor samples were not included in our study.<br>Human experimental procedures were approved by the Institutional Review Board and Ethics committee of the Institut Curie Hospital group (approval February 12th, 2014) and CNIL (Commission Nationale de l'informatique et des Libertés) (Nº approval: 1674356 delivered March 30th, 2013).

Note that full information on the approval of the study protocol must also be provided in the manuscript.

Clinical data

Policy information about clinical studies

All manuscripts should comply with the ICMJEguidelines for publication of clinical research and a completedCONSORT checklist must be included with all submissions.

Clinical trial registration	NA
Study protocol	NA
Data collection	Prospective cohorts: all patients were included from 2015 to 2018<br>Retrospective cohort: All patients were included from 2004 to 2012.<br>Detailed descriptions of prospective and retrospective cohorts of breast cancer patients are available in Tables S1 and S2.
Outcomes	Detailed descriptions of prospective and retrospective cohorts of breast cancer patients are available in Tables S1 and S2.

Flow Cytometry

Plots

Confirm that:

☐ The axis labels state the marker and fluorochrome used (e.g. CD4-FITC).

☒ The axis scales are clearly visible. Include numbers along axes only for bottom left plot of group (a 'group' is an analysis of identical markers).

☒ All plots are contour plots with outliers or pseudocolor plots.

☐ A numerical value for number of cells or percentage (with statistics) is provided.

Methodology

Sample preparation	Fresh human breast cancer primary tumors and invaded lymph nodes were obtained directly from the operating room, after surgical specimen's macroscopic examination and selection of areas of interest for diagnosis by a pathologist. Samples were cut into small pieces (around 1mm3) and digested in a CO2-independent medium (Gibco #18045-054) supplemented with 150 µg/mL liberase (Roche #05401020001) and DNase I (Roche #11284932001) during 40 minutes (min) at 37°C with shaking (180 rpm). Cells were then filtrated through a 40 µm cell strainer (Fisher Scientific #223635447) and resuspended in PBS+ solution (PBS, Gibco #14190; EDTA 2 mM, Gibco #15575; Human Serum 1 %, BioWest #S4190-100) at a final concentration between 5 x 10^5 and 10^6 cells in 50 µl before staining.
Instrument	In both conditions (surface and intracellular staining), signals were acquired on the LSRFortessaTM analyzer (BD biosciences)
Software	Data analysis was performed using FlowJo version X 10.0.7r2.<br>FlowSom algorithm was performed using R packages FlowSOM (1.12.0) and flowCore (1.462), according to the methodology described in (Ref 45). Self-Organizing Map was performed on all EPCAM-, CD45-, CD31-, CD235a- cells from 16 primary tumors and 20 invaded lymph nodes in order to generate unified trees based on the expression of the 5 CAF markers.
Cell population abundance	Although the content of CAF is variable from one freh sample to the other, in average, the number of CAF analyzed per tumor was 13 000 CAF.
Gating strategy	Cells were first gated based on forward (FSC-A) and side (SSC-A) scatters (measuring cell size and granularity, respectively) to exclude debris. Dead cells were excluded based on their positive staining for Live/Dead (fixed conditions) or DAPI (surface staining). Single cells were next selected based on SSC-A versus SSC-W parameters. Gating included EPCAM-, CD45-, CD31-, CD235a- cells, to remove epithelial (EPCAM+), hematopoietic (CD45+), endothelial (CD31+) and red blood cells (CD235a+). Cells from the negative fraction were next examined using the 5 CAF markers FAP, CD29, αSMA, PDGFRβ and PDPN.

☒ Tick this box to confirm that a figure exemplifying the gating strategy is provided in the Supplementary Information.
