# Supplementary material for: Cancer-associated fibroblast heterogeneity in axillary lymph nodes drives metastases in breast cancer through complementary mechanisms
Source: Nat Commun. 2020 Jan 21;11:404. doi: 10.1038/s41467-019-14134-w (PMC6972713; doi:10.1038/s41467-019-14134-w)
Supplement: Supplementary file 2 — Supplementary data [file 41467_2019_14134_MOESM2_ESM.pdf]

Reviewers' Comments:

Reviewer #1:
Remarks to the Author:
In their manuscript Pelon et al describe the role of CAF subtypes in LN metastases of breast cancer patients. This work follows up on previous work published by this group in which 4 CAF subtypes were characterized in primary breast tumors. Here they use the same markers for these CAF subtypes to show that the 4 CAF subtypes exist also in LN mets, and that two of these subtypes, S1 and S4, are particularly enriched in LN mets. They then isolate S1 and S4 CAFs and functionally characterize them in vitro, with and without co-culture with cancer cells. While both CAFs promote cancer cell proliferation, this characterization shows that S1 CAFs have higher motility, whereas S4 CAFs have a stronger contractile phenotype. Moreover, S1 initiate EMT in cancer cells, whereas S4 promote cancer cell invasion through remodeling of ECM. Finally, they analyze the correlation of S1 and S4 with disease outcome, and claim that high stromal content, in particular that enriched with S4 CAFs is correlated with worse outcome and with liver metastases.

This is an elegant work, with extensive functional analysis and potential clinical relevance. Moreover, the characterization of metastatic CAF subtypes has not been addressed before in such details. It is of interest to the field of tumor microenvironment research but also generally to cancer researchers, as well as clinicians. Nevertheless, some statements are not sufficiently supported by the experimental results presented, and further experiments and analyses should be performed before this manuscript can be accepted for publication.

Major comments;
1. One of the most important and novel points in this paper is the functional characterization of the two CAF subtypes S1 and S4. SInce most of the characterization is done in vitro, one must make sure that this is well controlled. Grown alone, in cell culture, S1 and S4 show different phenotypes. When cocultured with cancer cells these differences are not as significant and some of the effects are very similar between S1 and S4. The main differences and the interesting mechanistic explanation is in figure 6 – the differences between TGF-beta + CXCL12 in S1 vs Notch in S4. However the authors don't show the effect of the reciprocal – what would inhibition of TGF-beta and CXCL12 do to S4 CAFs and what would inhibition of Notch signaling do to S1 CAFs? It is crucial to show that this would not affect the activity of the reciprocal CAF subtype.
2. The clinical relevance of the findings is described in figure 7. The definition of high/low stroma in figure 7 is based on the epithelial compartment staining with EPCAM. Epithelial cells undergoing EMT loose EPCAM staining. As the authors themselves claim, their CAF subtypes promote EMT, in vitro, and therefore some of the EPCAM negative regions may actually be cancer cells that underwent EMT. To exclude this possibility the authors should stain with a marker that is less affected by EMT, or validate the epcam negative regions to be CAF positive using the S1 and S4 markers. This is particularly concerning since the correlations with outcome are borderline significant or not significant, in most cases. Stroma % is correlated with disease free survival, but not with overall survival, however S4 enrichment in stroma high tumors is correlated with overall survival, but not with disease-free survival. Figure 7H seems to be a derivative of Figure 7G, in which case multiple comparisons were made and the results, as shown in 7G, are not statistically significant. The authors also claim that CAF subset specific accumulation is independent from BC subtype and LN status as a prognostic indicator, but the multivariate analysis shows that it is clearly dependent on another factor. The authors should better explain the multivariate analysis (and whether it is additive or multiplicative).
3. One more concern is in the definition of the 4 CAF subtypes, which is based on previous work by this group, but is tested again here, since these are different CAFs. This definition is based on exclusion of cancer cells and immune cells, as well as positive selection for several markers defined in their earlier publication. The S4 subtype shows high expression of pericyte markers such as CSPG4 and RGS5 (as the authors point out in the methods section when they say that S4 have a pericyte-like signature) suggesting that some of these cells may be pericytes, or pericyte-derived CAFs. CAF S2 and S3 exhibit low expression of almost all of the CAF markers defined, and are in

fact present in non-metastatic LN, therefore it may be misleading to refer to them as CAFs, and perhaps they should be defined normal LN fibroblasts and not CAFs.

Minor comment: all fonts in FIg S5 are distorted.

In summary, this is an interesting paper, with novel insights and important mechanistic work. Once the issues raised above are addressed, it should definitely be considered for publication.

Reviewer #2:
Remarks to the Author:
This study contains a large set of analyses from the Mechta-Grigoriou group which continue earlier important and ground-breaking definitions, and functional and molecular annotations, of multi-marker-defined subsets of cancer-associated fibroblasts in human clinical samples. The novelty of the present study relies on analyses of breast cancer CAFs from invaded, and non-invaded, lymph nodes.

The wide-ranging study makes a number of novel observations;
-node-derived CAFs are composed of a similar set of four subsets of marker-defined CAFs as previously described in primary tumors (Fig. 1A-F)

- in lymph nodes S1 and S4 subsets are positively associated, and S2 and S3 subsets negatively associated, with invasion of malignant cells (Fig. 1G)
- when these subsets are used for case-based classification of lymph-node, CAF status significant differ between molecular sub-types (Fig. 2 E)
- CAF subsets S1 and S4 occur more commonly in lymph nodes than in primary tumors (Fig. 2 G)
- concordance between primary tumor status and lymph node status is high for "S1- and S4-primary tumors" but low for "S2- and S3-primary tumors" which display mostly S4-lymph nodes (2H-J)
- S1 and S4 CAFs, regardless of primary tumor or lymph-node origin, display distinct global gene expression profiles (Fig. 3)
- S1 and S4 CAFs show different cell-intrinsic functional characteristics (Fig. 4), and regulate cancer cell migration (Fig. 5) and cancer cell invasion (Fig. 6) in a differential manner
- Node S1-S4 status is associated with outcome (Fig. 7)
- additional data also report on associations between single-marker-defined case-status and associations with molecular sub-types (Fig. 2 A-C)
The study thus presents a set of novel observation regarding human breast cancer CAF of potential clinical relevance.

Some opportunities for improvements and clarifications are suggested.

Major points:

1. The main claim of the study is that FACS-based profiling of CAFs with five markers, which could theoretically yield 32 CAF subsets if each cell is classified as negative or positive for each marker, results in identification of four similar major subsets when analyses are performed in either primary tumors and lymph nodes (Fig. 1). This claim appears to be best supported by the un-supervised analyses of Fig. 1 D and E, data of Fig. 1B and the part of the results text listing defining marker characteristics of the different subsets (end of middle paragraph, results part 6).

To increase clarity authors can consider to re-format Fig. 1A-F and the corresponding results part to highlight these findings.

2. Some multiplexed IHC analyses, with cell-based resolution, validating in situ the existence of

the S1-4 cell types, and possibly also absence of other potential subsets, would significantly increase quality of the study.

3. IHC-based annotation of CAF phenotypes in Fig. 2 is based on a marker panel where one of the markers of the FACS panel (podoplanin) has been omitted and substituted with a new marker; FSP-1. This raises some concerns since the classification criteria is thus different in the FACS and IHC-based analyses.
Authors should discuss this and present data that FACS-analyses not using podoplanin, and IHC analyses not using FSP, does not change in a major way subset-assignment of cells in Fig. 1 or S1-S4 classification of cases in Fig. 2 and 7.

4. S1-S4 are used as "cell-classes" in Fig. 1 and 3-6, and as "case-classes" in Fig. 2 and 7. Clarity of the study would be improved if some data are reported regarding S1-S4 cellular heterogeneity within the different S1-S4 cases.

5. The description of the gene-expression profiling experiment of Fig. 3 should include the criteria used to define cells as S1 or S4.

Are this analyses on fibroblasts from S1 and S4 cases (only defined as negative for CD45/EPCAM/CD31/CD235a), or analyses of FACS-defined S1 and S4 cells?

6. Text should clarify whether the S1 and S4 cells of the functional experiments (Fig. 4-6) are from lymph node or primary tumor S1 and S4 cells. Furthermore, text should clarify if S1 and S4 cells are from S1 and S4 cases, or FACS-sorted cells full-filling the marker criteria of Fig. 1.

7. The earlier Cancer Cell study demonstrated differential functional effects of S1 and S4 CAFs with regard to modulation of different immune cells. Some S1/S4 differential key immune-modulatory phenotypes should be re-capitulated with lymph-node-derived S1 and S4 cells.

8. Survival associations should be discussed more cautiously. Notably the only significant signal in the multi-variable analyses is for S4 in overall survival, where no signal was detected in the uni-variable analyses (Table 1). Furthermore, it would be interesting to know the survival association of S1-4 status in the primary tumors.

9. S1- and S4-case status is associated with molecular subsets as shown in Fig. 2E. Earlier studies have shown different metastatic behavior of molecular sub-types. Some analyses should thus be presented to see if the general and liver-specific metastasis associations of Figs. 7 D-H remain after adjusting to molecular sub-type.

Reviewer #3:
Remarks to the Author:
In the manuscript titled 'Cancer-associated fibroblast heterogeneity in axillary lymph nodes drives metastases in breast cancers through complementary mechanisms', Pelon and colleagues have;
• Investigated cancer-associated fibroblast (CAF) heterogeneity and its role in metastasis in breast cancer.
• They have identified 4 distinct subsets of CAFs (CAF-S1, -S2, -S3 and -S4) using multicolor flow cytometry on a combination of 5 different CAF markers. Furthermore, to validate the existence and correspondence of the distinct subsets in the matched primary tumor and invaded axillary lymph node, they used FlowSom, an unsupervised automated algorithm, which orders cells according to their phenotypic similarities.
• Two of CAF subsets, CAF-S1, and CAF-S4, correlated to cancer cell invasion and that they exert positive but distinct functions in tumor dissemination. While CAF-S1 promote cancer cell migration and initiate an epithelial-to-mesenchymal transition through CXCL12 and TGFβ pathways. The

highly contractile subset CAF-S4 promotes cancer cell invasion in a 3-dimensions model via NOTCH signaling.

• Interestingly, the transcriptomic analysis suggested that the tumor cells and CAF-subsets S1 and S4, at the primary site overlap with its respective matched axillary lymph nodes cells, suggesting that the tumor cells and CAF subset -S1 and -S4 are molecularly close at the two sites.

• Furthermore, the authors performed phenotypic and functional experiments to examine the intrinsic properties of CAF-S1 and CAF-S4 subsets in primary cell cultures from BC patients.

o CAF-S1 and CAF-S4 exhibit distinct migration and invasion properties, wherein, the CAF-S1 subset were high motile and initiate first of EMT and secrete factors that attract cancer cells

o CAF-S4 exhibited a strong contractile phenotype with a clear ability to pull on collagen fibers and remodel the matrix and promote cancer cell invasion.

o They identified specific molecular factors that are involved in the distinct pro-invasive properties of CAF-S1 and CAF-S4 subsets. The authors show the role of TGFβ and CXCL12 signaling pathways in promoting pro-tumorigenic phenotype via the CAF-S1 subset. Additionally, they demonstrate that CAF-S4 subset of fibroblasts stimulates cancer cell invasion and motility in 3D by increasing contractility through the NOTCH signaling pathway.

• Finally, the authors extended their studies to examine the clinical relevance of their findings, by using multivariate analysis on a retrospective cohort of breast cancer patients. More specifically, they demonstrate that patients who showed LN metastases with high stromal content on the one hand and specific enrichment of the CAF-S4 subset on the other were more prone to develop late distant metastases than any other patients.

Overall, the manuscript is well written and provides insight into the heterogeneity of cancer-associated fibroblasts and their role in the metastasis of breast cancer. The overall approach of using multicolor flow cytometry on a combination of 5 different known CAF markers combined with FlowSom to delineate phenotypic similarities seemed like a good approach. However, the overall claims lack novelty and fails to instill much enthusiasm. In the discussion section, the authors acknowledge that their findings are consistent with previous studies and not necessarily an extension. There are some suggestions that will strengthen the current state of the manuscript. TGF-beta and NOTCH signaling has been extensively studied in the context of fibroblast activation and its pro-tumorigenic functions. If the authors wish to inject novelty into their manuscript, they could consider using the transcriptomic data generated on the CAF subsets S1 and S4, in order to potentially identify novel interactions between CAF-subsets with cancer cells and then identify their respective mechanisms involved. A comprehensive cytokine array on the conditioned media (coculture of different subsets of fibroblasts with carcinoma cells) combined with the transcriptomic data might provide some insight into the possible heterotypic interactions via cytokines/chemokines that might, in turn, lead to fibroblast activation and its pro-tumorigenic functions.

Detailed reply to the reviewers' comments

Revised manuscript (NCOMMS-19-04790) Pelon et al, "Cancer-associated fibroblast heterogeneity in axillary lymph nodes drives metastases in breast cancers through complementary mechanisms".

We first would like to thank the Editor and the three Reviewers for considering our work suitable for publication in *Nature Communications*. We have now included in the new version of the text the modifications (in apparent in the new text) for addressing their comments. For better visualization of the discussion, initial comments of the reviewers are below indicated in blue and our answers in black.

Reviewers' Comments:

Reviewer #1:

In their manuscript Pelon et al describe the role of CAF subtypes in LN metastases of breast cancer patients. This work follows up on previous work published by this group in which 4 CAF subtypes were characterized in primary breast tumors. Here they use the same markers for these CAF subtypes to show that the 4 CAF subtypes exist also in LN mets, and that two of these subtypes, S1 and S4, are particularly enriched in LN mets. They then isolate S1 and S4 CAFs and functionally characterize them in vitro, with and without co-culture with cancer cells. While both CAFs promote cancer cell proliferation, this characterization shows that S1 CAFs have higher motility, whereas S4 CAFs have a stronger contractile phenotype. Moreover, S1 initiate EMT in cancer cells, whereas S4 promote cancer cell invasion through remodeling of ECM. Finally, they analyze the correlation of S1 and S4 with disease outcome, and claim that high stromal content, in particular that enriched with S4 CAFs is correlated with worse outcome and with liver metastases.

This is an elegant work, with extensive functional analysis and potential clinical relevance. Moreover, the characterization of metastatic CAF subtypes has not been addressed before in such details. It is of interest to the field of tumor microenvironment research but also generally to cancer researchers, as well as clinicians. Nevertheless, some statements are not sufficiently supported by the experimental results presented, and further experiments and analyses should be performed before this manuscript can be accepted for publication.

We are really grateful to the Reviewer for the positive evaluation of our manuscript and for his/her interesting suggestions. We have now considered Reviewer's comments and provided the corresponding modifications, as recommended.

Major comments:

1. One of the most important and novel points in this paper is the functional characterization of the two CAF subtypes S1 and S4. Since most of the characterization is done in vitro, one must make sure that this is well controlled. Grown alone, in cell culture, S1 and S4 show different phenotypes. When cocultured with cancer cells these differences are not as significant and some of the effects are very similar between S1 and S4. The main differences and the interesting mechanistic explanation is in figure 6 – the differences between TGF-beta + CXCL12 in S1 vs Notch in S4. However the authors don't show the effect of the reciprocal – what would inhibition of TGF-beta and CXCL12 do to S4 CAFs and what would inhibition of Notch signaling do to S1 CAFs? It is crucial to show that this would not affect the activity of the reciprocal CAF subtype.

We thank the reviewer for this interesting suggestion. We have now addressed the question of the reciprocal effects between CAF-S1 and CAF-S4 subsets. We have inhibited the identified pathways in both CAF-S1 and S4 and compared the effects on their respective activities, as requested. To do so, we have investigated the impact of CXCL12 and TGFβ inactivation in CAF-S4, and of NOTCH in CAF-S1, thereby providing the reciprocal analyses to those initially given. While CXCL12 silencing in CAF-S1 fibroblasts prevents their effects on cancer cell density and E-cadherin staining in both MCF7 and T47D, inhibiting CXCL12 in CAF-S4 has no impact. Similarly, inhibiting TGFR by using LY2109761 in CAF-S1 has a significant impact on cancer cell density and E-cadherin staining in both MCF7 and T47D, as the treatment in CAF-S1 almost completely rescues the cancer cell phenotype. In contrast, although existing, the rescue effect observed on EMT initiation of cancer cell upon TGFR inhibition in CAF-S4 are at lower extent than in CAF-S1. Finally, we tested the effect of NOTCH pathway inhibition in CAF-S1 using DAPT but observed no impact of this inhibition in CAF-S1 on contractile capacity, while this inhibitor significantly reduced ECM contraction when applied in CAF-S4.

In conclusion, these data highlight either the lack of impact or the moderate effect of the pathways identified in CAF-S1/S4 in the reciprocal cell lines. These new data have now been included into the **new Figures 6a, 6c and 6d,** with additive photos shown in **Supplementary Figures 5c and i.** The corresponding text is modified **p14-15.** These observations reinforce our initial data and we thank the reviewer for having suggested these complementary analyses.

2. The clinical relevance of the findings is described in figure 7. The definition of high/low stroma in figure 7 is based on the epithelial compartment staining with EPCAM. Epithelial cells undergoing EMT loose EPCAM staining. As the authors themselves claim, their CAF subtypes promote EMT, in vitro, and therefore some of the EPCAM negative regions may actually be cancer cells that underwent EMT. To exclude this possibility the authors should stain with a marker that is less affected by EMT, or validate the epcam negative regions to be CAF positive using the S1 and S4 markers. This is particularly concerning since the correlations with outcome are borderline significant or not significant, in most cases. Stroma % is correlated with disease free survival, but not with overall survival, however S4 enrichment in stroma high tumors is correlated with overall survival, but not with disease-free survival. Figure 7H seems to be a derivative of Figure 7G, in which case multiple comparisons were made and the results, as shown in 7G, are not statistically significant.
The authors also claim that CAF subset specific accumulation is independent from BC subtype and LN status as a prognostic indicator, but the multivariate analysis shows that it is clearly dependent on another factor. The authors should better explain the multivariate analysis (and whether it is additive or multiplicative).

As requested by the Reviewer, we have now brought a substantial number of precisions and additive analyses regarding the clinical relevance and survival impact of our findings.

The percentage of stroma in each section has now been quantified by a referent Pathologist using a conventional approach based on morphological criteria, as well as by an automated approach (QuantCenter, 3DHistech) applied by an independent researcher. In addition to EPCAM staining, we have also performed a staining for E-cadherin and analyzed these staining in regards of CAF markers for an accurate evaluation of both stromal and epithelial compartments. These different evaluations of the percentage of stroma / epithelium in invaded lymph nodes (LN) gave consistent results, whatever the person (pathologist *versus* researcher), the method (manually *versus* automated) or the staining (EPCAM / E-cadherin). We have thus now introduced the different ways by which we have evaluated and compared the percentage of stromal and epithelial compartments in the **Methods** (# Immunochemistry analyses, **p 27**) and Results' (**p 16**) sections.

As requested, we have now given precisions on the multivariate analyses done. The multivariate analyses using the Cox regression model evaluate the different parameters (i.e. stromal content, CAF subset enrichment, LN status and BC subtype) in an additive manner. As asked by the referee, when we applied a Cox analysis using a multiplicative method to our set of data, we observed that even when considering only LN status and BC subtypes, two major well-known independent survival indicators for BC patients, the result was not significant. This suggests that the multiplicative method might not be the most appropriated for evaluating variables with the number of patients we have access to. The additivity of the Cox regression test is now mentioned in the **Results (**end of **p 16),** in the **Figure legend of Table 1, p57** and in the **Methods** (# Statistical analyses, **p43),** to avoid any misunderstanding, as recommended.

Regarding the survival analyses, we have performed additive analyses for providing precisions. Indeed, in the Cox regression model shown in the first version of the text, we had previously analyzed patients with low and high percentage of stroma together. Based on results using Log Rank test (Kaplan-Meier survival curves shown in Figure 7C), we sought to test the independence of CAF subset accumulation from LN status and BC subtype, considering patients with low- or high-percentages of stroma, separately. We observed that the independence is absolutely not detected in patients with low-percentages of stroma, while it is statistically significant in patients with high-percentages of stroma (**new Supplementary Table 6**). Thus, as mentioned by the reviewer, the CAF subset is a prognostic factor for overall survival, only in a context of high stromal content. Among patients who displayed high stromal content in LN at diagnosis, those enriched in CAF-S4 showed the poorest overall survival, while no difference was observed in patients with low stromal content. This underlines the importance of LN stromal content, both in terms of quantity and quality. These new analyses are now described in the **new text p17.** We thank the reviewer for his/her comment.

3. One more concern is in the definition of the 4 CAF subtypes, which is based on previous work by this group, but is tested again here, since these are different CAFs. This definition is based on exclusion of cancer cells and immune cells, as well as positive selection for several markers defined in their earlier publication. The S4 subtype shows high expression of pericyte markers such as CSPG4 and RGS5 (as the authors point out in the methods section when they say that S4 have a pericyte-like signature) suggesting that some of these cells may be pericytes, or pericyte-derived CAFs. CAF S2 and S3 exhibit low expression of almost all of the CAF markers defined, and are in fact present in non-metastatic LN, therefore it may be misleading to refer to them as CAFs, and perhaps they should be defined normal LN fibroblasts and not CAFs.

We fully agree with Reviewer's comments. As mentioned, the CAF-S4 exhibit pericyte-signature, and might indeed derived from pericytes. In addition to our data in breast and ovarian cancers, the existence of this particular CAF subtype has also been described in several studies, and their pericyte-origin previously discussed (including Hosaka, 2016; Murgai, 2017; Bartochek 2018; Di Carlo 2018). We have cited these publications in our manuscript (in the Results' section, **p9** and in the Discussion, **p21**). In addition, we have also included an MCAM immunostaining in the new version of our manuscript to show that MCAM-positive CAF can be detected in invaded breast cancer lymph nodes, thereby confirming their pericyte-like signature. The staining is now shown in the **new Supplementary Figure 2f (**Result described **p9).**

Finally, CAF-S2 and CAF-S3 show low expression of CAF markers and are observed in healthy tissues, as shown in our previous paper (Costa, 2018). We thus do agree with the Reviewer that these fibroblasts might indeed be or derived from normal resident fibroblasts. As they are detected in BC, to spell them "normal fibroblasts" might be also questioned or contested. Still, as we completely agree with the Reviewer on that point, we have detailed this important point in the **new version of the manuscript** (**p6**) for being more precise in the definition that we can give to these fibroblasts.

Minor comment: all fonts in FIg S5 are distorted.

We have checked the font in Figure S5 and have tried to solve this problem. We thank the Reviewer for having noticed that point and his/her careful reading of our paper.

In summary, this is an interesting paper, with novel insights and important mechanistic work. Once the issues raised above are addressed, it should definitely be considered for publication.

We would like to thank again the reviewer for his/her positive evaluation and support of our work.

Reviewer #2 (Remarks to the Author):

This study contains a large set of analyses from the Mechta-Grigoriou group which continue earlier important and ground-breaking definitions, and functional and molecular annotations, of multi-marker-defined subsets of cancer-associated fibroblasts in human clinical samples. The novelty of the present study relies on analyses of breast cancer CAFs from invaded, and non-invaded, lymph nodes.
The wide-ranging study makes a number of novel observations;
-node-derived CAFs are composed of a similar set of four subsets of marker-defined CAFs as previously described in primary tumors (Fig. 1A-F)
- in lymph nodes S1 and S4 subsets are positively associated, and S2 and S3 subsets negatively associated, with invasion of malignant cells (Fig. 1G)
- when these subsets are used for case-based classification of lymph-node, CAF status significant differ between molecular sub-types (Fig. 2 E)
- CAF subsets S1 and S4 occur more commonly in lymph nodes than in primary tumors (Fig. 2 G)
- concordance between primary tumor status and lymph node status is high for "S1- and S4-primary tumors" but low for "S2- and S3-primary tumors" which display mostly S4-lymph nodes (2H-J)
- S1 and S4 CAFs, regardless of primary tumor or lymph-node origin, display distinct global gene expression profiles (Fig. 3)
- S1 and S4 CAFs show different cell-intrinsic functional characteristics (Fig. 4), and regulate cancer cell migration (Fig. 5) and cancer cell invasion (Fig. 6) in a differential manner
- Node S1-S4 status is associated with outcome (Fig. 7)
- additional data also report on associations between single-marker-defined case-status and associations with molecular sub-types (Fig. 2 A-C)

The study thus presents a set of novel observation regarding human breast cancer CAF of potential clinical relevance.

We are grateful to the Reviewer for his/her positive evaluation of our work. We have appreciated his/her comments that contributed to improve the quality of our manuscript. We are very pleased to list below the main modifications inserted into the text and figures.

Some opportunities for improvements and clarifications are suggested.

Major points:
1. The main claim of the study is that FACS-based profiling of CAFs with five markers, which could theoretically yield 32 CAF subsets if each cell is classified as negative or positive for each marker, results in identification of four similar major subsets when analyses are performed in either primary tumors and lymph nodes (Fig. 1). This claim appears to be best supported by the un-supervised analyses of Fig. 1 D and E, data of Fig. 1B and the part of the results text listing defining marker characteristics of the different subsets (end of middle paragraph, results part 6).
To increase clarity authors can consider to re-format Fig. 1A-F and the corresponding results part to highlight these findings.

As suggested by the Reviewer, the data has now been re-organized and presented in a different and more convincing way. As recommended, we first described the identification of the 4 main CAF subsets in invaded breast cancer lymph nodes (BC LN) by using FlowSom unsupervised analyses and next quantified expression of each CAF marker in gated subpopulations. In line with this, we have also re-written the text for highlighting that in contrast to CAF-S2 and CAF-S3 also detected in healthy tissues, CAF-S1 and CAF-S4 strictly accumulate in invaded LN. Moreover, we underline that CAF-S1 and CAF-S4 are correlated with tumor cell invasion, revealing their potential function in metastatic spread in BC. The data are now re-organized in the **New Figure 1** and described accordingly in the **new corresponding text, p5 and 6.**

2. Some multiplexed IHC analyses, with cell-based resolution, validating in situ the existence of the S1-4 cell types, and possibly also absence of other potential subsets, would significantly increase quality of the study.

As requested, we have performed analyses at cell-based resolution of CAF subsets in invaded LN. To do so, we performed maps of CAF subsets at cellular scale by using IHC staining of each CAF marker We have previously developed an image analysis tool that we applied to breast and ovarian cancers (Costa, 2018; Givel, 2018). We have now applied this tool to invaded LN. In brief, images from IHC staining of CAF markers from the same areas in representative invaded LN are first aligned using elastic transformation from Fiji software plugin (bUnwarpJ). This plugin uses landmarks manually defined on hematoxylin & eosin staining of the sections to compute the optimal correlation between images and alignment at cellular level. Images are divided into tiles of 225 μm^2 to mimic the approximate size of one fibroblast and each tile is annotated according to the position in the section. Aligned and annotated images of the CAF markers are then submitted to color deconvolution and the intensity of each DAB staining is measured by densitometry analysis using ImageJ software. Each tile is classified into a specific CAF subset using the algorithm tree shown in **Figure 2d**, which takes as input DAB intensities of CAF markers measured within each tile. The method is also described in details in the **Method** section, **p27-28** (# Design of a decision tree for CAF subset prediction, and # Visualization of CAF subsets and epithelial cells at cellular level *in situ* in invaded LN). Different examples of maps of CAF-S1 or CAF-S4 enriched LN are now shown in the new version of our paper in new **Figure 2f** and **Supplementary Figure 2d**, and described **p7**.

3. IHC-based annotation of CAF phenotypes in Fig. 2 is based on a marker panel where one of the markers of the FACS panel (podoplanin) has been omitted and substituted with a new marker; FSP-1. This raises some concerns since the classification criteria is thus different in the FACS and IHC-based analyses.
Authors should discuss this and present data that FACS-analyses not using podoplanin, and IHC analyses not using FSP, does not change in a major way subset-assignment of cells in Fig. 1 or S1-S4 classification of cases in Fig. 2 and 7.

We have initially introduced Podoplanin (PDPN) in our FACS panel because PDPN is known to stain resident fibroblasts in normal LN structure. According to our first observations by FACS, we have sought to stain PDPN by IHC, but we failed to find an antibody giving a satisfying signal intensity and specificity by IHC. In that aim, we have tested different antibodies, including the anti-human PDPN monoclonal antibody NZ-1 (AngioBio, #11-009), and different conditions. But we had some difficulties to obtain a staining (even when using Tonsil sections), and when we get a staining, this one was not reproducible. Thus, we have not been able to find appropriate conditions for PDPN staining by IHC. However, as recommended by the Reviewer, we have checked that FSP1 and PDPN staining recognize the same fibroblasts. As FSP1 and PDPN antibodies are both available for FACS, we have now included in the new manuscript an experiment showing that using either PDPN or FSP1 antibody does not affect the detection of CAF sub-population. We found that using one or the other antibody gives rise to the same results and identify the same cells. This shows that these two antibodies provide redundant information and identify the same type of cells. These data are now shown in the **Supplementary Figure 2b** and described in the new version of the text **p6.**

4. S1-S4 are used as “cell-classes” in Fig. 1 and 3-6, and as “case-classes” in Fig. 2 and 7. Clarity of the study would be improved if some data are reported regarding S1-S4 cellular heterogeneity within the different S1-S4 cases.

As mentioned by the referee, FACS as well as functional assays provide data at a cellular scale. The cellular resolution from IHC data described in point 2 above with the use of the image analysis tool and now added in **Figure 2f** and **Supplementary Figure 2d** is technically challenging. This analysis is really time-consuming and impossible to generate for all data. We have thus chosen to use the notion of CAF subset-enrichment in patients when analyses were based on IHC. Based on CAF marker expression, we observed that the median of fibroblasts positive for FAP, SMA and CD29 (reflecting CAF-S1 identity) reaches 75% of total CAF in patients defined as CAF-S1-enriched LN. Moreover, the median of fibroblasts negative for FAP but positive for SMA and CD29 (reflecting CAF-S4 identity) reaches 60% of total fibroblasts in CAF-S4-enriched LN. These quantifications have been established by re-evaluating all patients analyzed by IHC and are now provided in the new version of the text in the **Results’** section, **p7**.

5. The description of the gene-expression profiling experiment of Fig. 3 should include the criteria used to define cells as S1 or S4. Are this analysis on fibroblasts from S1 and S4 cases (only defined as negative for CD45/EPCAM/CD31/CD235a), or analyses of FACS-defined S1 and S4 cells?

We apologize for the missing information. The CAF analyzed in **Figure 3** were isolated by FACS and the gating strategy combined surface markers, as described in **Figure 1.** Indeed, cells were first selected by using live-dead, CD45, EPCAM, CD235a, CD31 markers to exclude dead, hematopoietic, epithelial, and endothelial cells, as well as erythrocytes. Cells were next stained with CAF markers. These precisions are now given in the **new text** (section # *CAF subsets show similar molecular identity between PT and metastatic LN*), **p8**.

6. Text should clarify whether the S1 and S4 cells of the functional experiments (Fig. 4-6) are from lymph node or primary tumor S1 and S4 cells. Furthermore, text should clarify if S1 and S4 cells are from S1 and S4 cases, or FACS-sorted cells full-filling the marker criteria of Fig. 1.

As recommended, we have now given the proportion of CAF-S1 and CAF-S4 cell lines used for functional assays that were isolated either from PT or LN. In brief, all along the duration of the project, we isolated 22 CAF-S1 and 22 CAF-S4 primary cell lines using FACS sorted method that were extracted from the same samples and used in parallel for functional experiments, as shown in **Figure 1**. Among them, 64% were from PT and 36% from LN. In agreement with the strong transcriptomic analogy of CAF subsets from PT or LN (as shown in **Figure 3**), we did not observe any difference in functional assays when we performed experiments with CAF-S1/CAF-S4 isolated either from PT or from LN. Thus, we observed no effect of the site of isolation (PT or LN) on CAF-S1/CAF-S4 properties. Transcriptomic signatures and *in vitro* properties were thus equivalent whatever the site of CAF initial localization (PT or LN). This was something that we also observed when we isolated CAF-S1 from either luminal or triple-negative breast cancers (data published previously in Costa, 2018). Still, this is an important precision that has now been included in the new text. We thank the Reviewer for his suggestion, which improves the clarity of our manuscript. As recommended by the Reviewer, we have now described the different primary cell lines used in the **Method’**s section **p29-30**.

7. The earlier Cancer Cell study demonstrated differential functional effects of S1 and S4 CAFs with regard to modulation of different immune cells. Some S1/S4 differential key immune-modulatory phenotypes should be re-capitulated with lymph-node-derived S1 and S4 cells.

As requested, we have performed an analysis testing the link between FOXP3+ regulatory T lymphocytes and CAF-S1 subset in invaded lymph nodes, as done in our previous study in PT from TN BC patients. Indeed, as indicated by the Reviewer, we have previously observed that TN BC can be divided into two subgroups, being either CAF-S1- or CAF-S4-enriched in PT. In our previous study, we found that there is no significant difference in CD8 and CD3 T cell infiltration in CAF-S1- or CAF-S4-enriched TN BC, but there is a significant accumulation of FOXP3+ T lymphocytes in CAF-S1-enriched tumors compared to CAF-S4 (n = 72 TN BC). We thus performed CD3+, CD8+ and FOXP3+ T cell staining in invaded LN of TN BC patients. Among the cohort analyzed in our paper, we had access to 28 samples from TN BC patients with enough remaining material for proceeding to these different staining. Although we found a similar tendency in LN with a higher amount of FOXP3+ T lymphocytes in CAF-S1 compared to CAF-S4-enriched LN, it does not reach significance. Here we have to emphasize that the number of available patients in these different analyses is not the same, with a lower proportion of patients in which we could analyze T cells in invaded LN. Thus, it is difficult to determine if the lack of significance is because CAF subsets are less efficient to modulate immune cells in LN than in PT or if this is due to the low number of patients and the lack of power in this analysis. As we suffer from a potential lack of power, the observations regarding the link between CAF subset enrichment in LN and at PS and FOXP3 T cell infiltration in the respective compartments deserve deeper analyses that -we hope the Reviewer will agree- are beyond the scope of the current study.

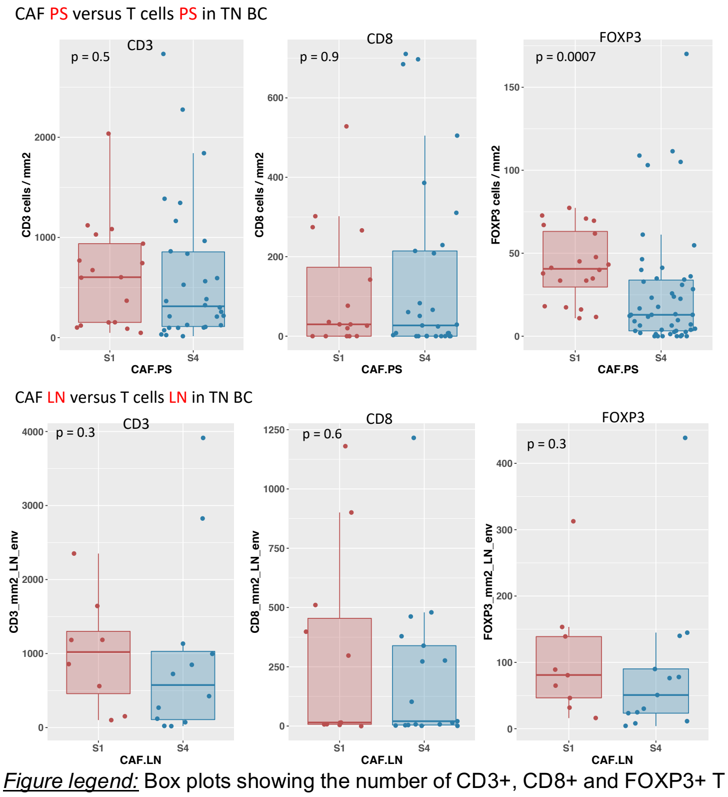

Figure legend: Box plots showing the number of CD3+, CD8+ and FOXP3+ T lymphocytes per mm^2 at primary sites (PS, Up) and in invaded lymph nodes (LN, Bottom) in CAF-S1 or CAF-S4-enriched tumors (Up) and CAF-S1 or CAF-S4-enriched invaded LN (Bottom).

8. Survival associations should be discussed more cautiously. Notably the only significant signal in the multi-variable analyses is for S4 in overall survival, where no signal was detected in the uni-variable analyses (Table 1). Furthermore, it would be interesting to know the survival association of S1-4 status in the primary tumors.

We agree with the reviewer that survival interpretation should be taken with caution, and we have now modified the text to describe more precisely the observations (**p16-18**). We have also included additive analyses in the new version of our manuscript in order to describe in a more precise manner survival

association. Indeed, we observed that the CAF subsets are a prognostic factor for overall survival but is strongly associated with stromal content. Indeed, by comparing the CAF subset prognostic value in patients with either low- or high-stromal content in LN, we found that this prognostic value was only observed in the context of high stromal content. Among patients with high stromal content in LN at diagnosis, those enriched in CAF-S4 showed the poorest overall survival, while no difference according to CAF subsets was detected in patients with low stromal content. We thus now show the CAF subset impact in patients with high stromal content in the **new Supplementary Table 6**, and describe this observation and the lack of CAF subset effects in patients with low stromal content in the **new text p16-17.** In contrast to LN, we did not observe any survival association of the CAF subset status in primary tumors (as indicated in our previous paper, Costa 2018). As this might reinforce the potential interest of its prognostic value -although faint- in LN, this notion is now indicated in the **new Results' section p17,** and **Discussion, p19.**

9. S1- and S4-case status is associated with molecular subsets as shown in Fig. 2E. Earlier studies have shown different metastatic behavior of molecular sub-types. Some analyses should thus be presented to see if the general and liver-specific metastasis associations of Figs. 7 D-H remain after adjusting to molecular sub-type.

This is an important point and we thank the Reviewer for his/her comment. Indeed, bones are the most common metastatic site in BC, in particular in Lum patients, but TN BC are also associated with high rate in other metastatic niches, such as liver. We have thus tested whether the prognosis value of global stromal content and CAF subset accumulation in LN at diagnosis could be linked to an enrichment in one specific BC subtype, as requested.

To address this question, we first verified that there was no enrichment in one specific BC subtype in each category defined according to LN features (low / high stromal content and CAF-S1 / CAF-S4 enrichment) in patients with distant metastases. Among patients with distant metastases, 45% were Lum, 13% HER2 and 42% TN. We had thus access to a quite low representation of HER2 patients but Lum and TN BC subtypes were equally represented. We observed a similar repartition of Lum and TN BC patients in the different categories (considering CAF content and subset status). We even noticed a higher proportion of Lum compared to TN patients in the category of CAF-S4-enriched LN with high stromal content (60 % Lum *versus* 33% TN). Thus, patients with high-CAF-S4 content in LN, who tend to metastasize into liver, are not enriched in TN BC subtype in the cohort analyzed. We can thus conclude that the effects we observed were not associated with an enrichment in one particular BC subtype. This important point is now mentioned in the **new text, p18.**

In addition, as requested, we have also performed each analysis in Figure 7 by considering each BC subtype. We observed that distribution in each subgroup (high / low stroma and CAF subset enrichment) is similar in the different BC subtypes, in particular if we compare Lum and TN BC patients with a similar number of patients studied. For each analysis done, the repartition in the different BC subtype is now provided in the **New Figure 7**.

<u>Reviewer #3 (Remarks to the Author):</u>

In the manuscript titled 'Cancer-associated fibroblast heterogeneity in axillary lymph nodes drives metastases in breast cancers through complementary mechanisms', Pelon and colleagues have;

• Investigated cancer-associated fibroblast (CAF) heterogeneity and its role in metastasis in breast cancer.

• They have identified 4 distinct subsets of CAFs (CAF-S1, -S2, -S3 and -S4) using multicolor flow cytometry on a combination of 5 different CAF markers. Furthermore, to validate the existence and correspondence of the distinct subsets in the matched primary tumor and invaded axillary lymph node, they used FlowSom, an unsupervised automated algorithm, which orders cells according to their phenotypic similarities.

• Two of CAF subsets, CAF-S1, and CAF-S4, correlated to cancer cell invasion and that they exert positive but distinct functions in tumor dissemination. While CAF-S1 promote cancer cell migration and initiate an epithelial-to-mesenchymal transition through CXCL12 and TGFβ pathways. The highly contractile subset CAF-S4 promotes cancer cell invasion in a 3-dimensions model via NOTCH signaling.

• Interestingly, the transcriptomic analysis suggested that the tumor cells and CAF-subsets S1 and S4, at the primary site overlap with its respective matched axillary lymph nodes cells, suggesting that the tumor cells and CAF subset -S1 and -S4 are molecularly close at the two sites.

• Furthermore, the authors performed phenotypic and functional experiments to examine the intrinsic properties of CAF-S1 and CAF-S4 subsets in primary cell cultures from BC patients.
o CAF-S1 and CAF-S4 exhibit distinct migration and invasion properties, wherein, the CAF-S1 subset were high motile and initiate first of EMT and secrete factors that attract cancer cells
o CAF-S4 exhibited a strong contractile phenotype with a clear ability to pull on collagen fibers and remodel the matrix and promote cancer cell invasion.
o They identified specific molecular factors that are involved in the distinct pro-invasive properties of CAF-S1 and CAF-S4 subsets. The authors show the role of TGFβ and CXCL12 signaling pathways in promoting pro-tumorigenic phenotype via the CAF-S1 subset. Additionally, they demonstrate that CAF-S4 subset of fibroblasts stimulates cancer cell invasion and motility in 3D by increasing contractility through the NOTCH signaling pathway.
• Finally, the authors extended their studies to examine the clinical relevance of their findings, by using multivariate analysis on a retrospective cohort of breast cancer patients. More specifically, they demonstrate that patients who showed LN metastases with high stromal content on the one hand and specific enrichment of the CAF-S4 subset on the other were more prone to develop late distant metastases than any other patients.

Overall, the manuscript is well written and provides insight into the heterogeneity of cancer-associated fibroblasts and their role in the metastasis of breast cancer. The overall approach of using multicolor flow cytometry on a combination of 5 different known CAF markers combined with FlowSom to delineate phenotypic similarities seemed like a good approach. However, the overall claims lack novelty and fails to instill much enthusiasm. In the discussion section, the authors acknowledge that their findings are consistent with previous studies and not necessarily an extension. There are some suggestions that will strengthen the current state of the manuscript. TGF-beta and NOTCH signaling has been extensively studied in the context of fibroblast activation and its pro-tumorigenic functions. If the authors wish to inject novelty into their manuscript, they could consider using the transcriptomic data generated on the CAF subsets S1 and S4, in order to potentially identify novel interactions between CAF-subsets with cancer cells and then identify their respective mechanisms involved. A comprehensive cytokine array on the conditioned media (coculture of different subsets of fibroblasts with carcinoma cells) combined with the transcriptomic data might provide some insight into the possible heterotypic interactions via cytokines/chemokines that might, in turn, lead to fibroblast activation and its pro-tumorigenic functions.

We first would like to thank the Reviewer for his/her positive evaluation of our work. We have now done our best to provide new data in order to answer to his/her requests,

We would like to emphasize that the novelty in this paper consists in 3 main points : (1) 2 specific CAF subsets (CAF-S1 and CAF-S4) strikingly accumulate in invaded lymph nodes (LN) and correlate with tumor cell invasion; (2) both CAF-S1 and CAF-S4 promote metastatic spread by exhibiting complementary activities; (3) high CAF content in invaded LN, in particular in CAF-S4, is associated with late distant metastases and constitutes a new prognostic factor, while CAF content in PT does not. Considering the Reviewer's comment, we have re-written different parts of our paper for clarifying these novel findings. We have emphasized these points in description of the question asked (**introduction p4**), in the description of CAF subsets in LN (**Figures 1 and 2, p5-8**), in the pathways involved in the complementary CAF-S1 and CAF-S4 activities (**Figure 6, p14-15**) and in the clinical relevance of our findings (**Figure 7, p16-18**). We hope these different changes will clarify our message and give satisfaction to the Reviewer.

As recommended by the Reviewer, we have tested cytokine arrays on the conditioned media from CAF-S1 and CAF-S4, in presence or not of tumor cells, for evaluating the release of several cytokines in the different conditions tested. We have also compared the release of these cytokines by CAF-S1 and CAF-S4 fibroblasts with the transcriptomic data initially shown in Figure 3. Thanks to this new recommended analysis, we provided a wider study of the different signals that could be exchanged between CAF-S1/S4 and tumor cells. Among them, we studied for instance IL6, IL8, CCL2, CXCL1, IL18 and SERPIN1. We found that these different molecules were either not detected, or not differentially released by the two CAF subsets in presence or not of cancer cells, and thus we could not explain by this way the phenotypes observed. Based on these new data, we can argue that the molecules, such as CXCL12, TGFβ and NOTCH that we analyzed in-depth in our manuscript are main pathways in the phenotypes we evaluated, such as EMT initiation of cancer cells and ECM contraction. We have now included these data in the **New Supplementary Figure 6i** and corresponding text **p16** of the new

version of our manuscript. We thank the reviewer for this suggestion that provides us a better vision of the molecules released by the different CAF subsets, in presence or absence of cancer cells.

Reviewers' Comments:

Reviewer #1:
Remarks to the Author:
In their revised manuscript Pelon et al have nicely addressed my comments and concerns. Overall I am satisfied with their response, and recommend this manuscript for publication. Some minor comments that should be addressed:

Following up on a comment I made in the earlier version regarding the normal-like phenotype of CAF-S2 and S3 (which the authors have addressed) – Both these CAF subtypes stain low or negative to most of the markers. Looking at Fig 1bc it seems that even when they are positive, in particular S3 (for CD29, FAP and PDPN), the pattern of the LN is different than that of the PT. This would make sense if these are indeed normal-like fibroblasts and therefore their tissue-specific identity is stronger than their cancer-related identity. The authors may want to comment on that, but even if not the fact that these are less similar between PT and LN than S1 and S4 should be addressed. This also is reflected in Fig 1e – it seems that the correlations are driven in some cases mostly from S1 (especially for PDGFr and PDPN) whereas S2 and S3 (and in the case of PDPN also S4) do not correlate.

The colors in Fig 2a-b are confusing – too similar to the colors used for the CAF subtypes. And the legend doesn't say what these colors mean. Also some dots are in a different color in some cases

Figure 7 – the use of green and red together should be avoided (for the color blind)

Page 10 – it should be mentioned that the CAF cultures are mostly from PT and only one is from LN

Reviewer #4:
None

Reviewer #5:
Remarks to the Author:
About the Traction Force Microscopy (TFM) data:
It would be nice to include the density and size of the fluorescent beads used for TFM, as they give an idea of the resolution for the used protocol. 20X is not a high magnification, thus the spatial resolution of the TFM data may be very low. However, the differences between the two groups are very clear.

In Figure 6e authors reported the strain energy density of CAF-S4 treated with DAPT and a significant difference is observed ($p=0.03$), However, supplementary figure 6e shows no significant differences ($p=0.08$) in stress upon DAPT treatment. Authors should specify and discuss in the text that they see a difference in strain and not in stress rather than claiming there is a general difference in traction force generation (lines 398 and 399 of the text).

About the SHG experiments:
The quantification of the collagen remodelling is somehow confusing. The authors measured the percentage area of collagen, but the cells seem to be embedded in collagen gels (2.5 mg/ml collagen solution 926 (Corning, #354249)). This may not be a good measure of collagen remodelling. Other metrics like alignment or density may be more adequate. I also do not understand how they calculate the median per cell (I would expect each cell to be a data point, are they calculating the percentage of collagen for several windows around the cell? Is it the median when considering the z stack?). The difference is evident from the images, but another descriptor may be much more robust than collagen area.

Additional comment:
What do they refer to as CAF-S1 and CAF-S4 pairs? How many cells do they analyse per pair? The authors seem to answer these for some of the figures; I recommend inviting them to put this information in the methods.

Detailed reply to the reviewers' comments

Revised manuscript (NCOMMS-19-04790) Pelon et al, "Cancer-associated fibroblast heterogeneity in axillary lymph nodes drives metastases in breast cancers through complementary mechanisms".

We first acknowledge the Editor for having found new Reviewers that bring a novel level of expertise and thus benefit to our paper. We thank all Reviewers for their positive evaluation of our work, and for considering our paper suitable for publication in *Nature Communications*. We have now included in the new version of the text the modifications in apparent for addressing their comments. For better visualization of the discussion below, initial comments of the reviewers are indicated in blue and our answers in black. In the new version of the manuscript, modifications are highlighted in yellow for differentiating them more easily from the revised version 1.

Reviewer #1 (Remarks to the Author):

In their revised manuscript Pelon et al have nicely addressed my comments and concerns. Overall I am satisfied with their response, and recommend this manuscript for publication. Some minor comments that should be addressed:

We thank the Reviewer for his/her positive assessment, and for previous and current recommendations that improve our manuscript. We are grateful for his/her careful reading of our manuscript.

Following up on a comment I made in the earlier version regarding the normal-like phenotype of CAF-S2 and S3 (which the authors have addressed) – Both these CAF subtypes stain low or negative to most of the markers. Looking at Fig 1bc it seems that even when they are positive, in particular S3 (for CD29, FAP and PDPN), the pattern of the LN is different than that of the PT. This would make sense if these are indeed normal-like fibroblasts and therefore their tissue-specific identity is stronger than their cancer-related identity. The authors may want to comment on that, but even if not the fact that these are less similar between PT and LN than S1 and S4 should be addressed. This also is reflected in Fig 1e – it seems that the correlations are driven in some cases mostly from S1 (especially for PDGFr and PDPN) whereas S2 and S3 (and in the case of PDPN also S4) do not correlate.

We have now mentioned this point in the new version of the text. As suggested, we now describe clearly that the pattern of CAF-S3 cells that might derive from normal-like fibroblasts is indeed different in LN compared to PT. The following sentence was added into the new text, **p6**, and we thank the Reviewer for his/her suggestion.

Added text: *Results, p6:* "In that sense, the pattern of CAF-S3 in LN was slightly different than the one detected in PT, as observed with CD29 staining (Fig. 1b-d), thereby suggesting that CAF deriving from normal-like fibroblasts could be more affected by their tissue of origin than CAF-S1 and CAF-S4".

The colors in Fig 2a-b are confusing – too similar to the colors used for the CAF subtypes. And the legend doesn't say what these colors mean. Also some dots are in a different color in some cases.

We are sorry for the confusion. We have now changed the colors appropriately in the **new Figure 2 a,b,** as recommended by the Reviewer. We use now violet for LumA, blue for LumB, grey for HER2, and black for TN breast cancer in **Figure 2 a,b**. Among HER2 patients, some of them were also categorized as LumB (as listed in the clinical data Table S1). We thus differentiated these patients as light grey for HER2 and dark grey for LumB/ HER2 patients, to avoid losing this information. As requested, we inserted a legend on the right of **Figure 2b panel** and added this information in the corresponding text (legend Figure 2a, **p52**).

Added text: *Legend Fig. 2a, p52:* **"**Percentage (%) of stroma in invaded LN according to BC subtypes (n = 124 patients), LumA (violet), LumB (blue), HER2 (light grey), Lum B/HER2 (dark grey), TN (black)".

Figure 7 – the use of green and red together should be avoided (for the color blind)

We are sorry for not having thought about that. As requested, we have now modified the colors used in **Figure 7a, b and d.** We have replaced green and red colors by grey (patients with low % of stroma)

and black (patients with high % of stroma) colors and made the corrections appropriately in the **new Figure 7a,b,d.**

Page 10 – it should be mentioned that the CAF cultures are mostly from PT and only one is from LN

In Figure 3, we have analyzed 5 pairs (PT and LN) of CAF-S1 and CAF-S4, meaning that we isolated these cell lines for both PT and LN. This precision has been given in the text, **p8.** This indication is also given in the legend of Figure 3, **p53.**

Experiments described in Figures 4, 5 and 6 were performed on both PT and LN CAF-S1/S4 cultures. Overall, we used 14 CAF couples from PT and 8 CAF couples from LN, as described in the Methods section (# *Cell culture*) and in Supplementary Table 1. We did our best to use CAF-S1/S4 both from PT and LN for each experiment, according to the cell cultures available at the time of experiments. As mentioned by the Reviewer, the validation by RNAseq shown in Supplementary Figure 3a-c was performed on 3 couples CAF S1/S4 from PT and one couple from LN. This information initially given in Supplementary Figure 3B has been also added in the **new Text p10**, as requested.

Added text: Results, p8: "CAF-S1 and CAF-S4 from both PT and LN (n = 5 pairs) were FACS-sorted"

Added text: Legend Fig. 3a, p53: **"**cells sorted from paired PT and LN (n = 5 pairs for CAF-S1 and CAF-S4)"

Added text: Results, p10: "We first confirmed by RNA sequencing on a first set of 4 pairs of CAF-S1 and CAF-S4 (3 couples of CAF-S1/CAF-S4 from PT and one couple from LN) that, after several passages in culture, these cells kept similar molecular identities than those detected in CAF-S1 and CAF-S4 patient samples without culture (Supplementary Fig. 3a-c)."

Reviewer #4 (Remarks to the Author):
We are grateful to the Reviewer for his/her support in our work and for considering our manuscript suitable for publication in *Nature Communications*, based on the revised version of our manuscript.

Reviewer #5 (Remarks to the Author):

About the Traction Force Microscopy (TFM) data:
It would be nice to include the density and size of the fluorescent beads used for TFM, as they give an idea of the resolution for the used protocol. 20X is not a high magnification, thus the spatial resolution of the TFM data may be very low. However, the differences between the two groups are very clear.

We thank the reviewer for pointing these oversights. As stated in the methods, we used commercial TFM gels (Matrigen, #ST-0.2YG-SW6G-COL-25 EA). These hydrogels contain fluorescent beads of 0.2 µm diameter at a density of 0.2 +/- 0.04 beads/µm². The last piv interrogation window used to track the bead displacement at the basis of the measurements of the traction forces was set to 48 x 48 pixel² (10.9 x 10.9 µm²) and thus contained about 20 beads. This was previously considered as adequate for such measurements (Martiel J-L, et al., 2015, Measurement of cell traction forces with ImageJ. *Methods in Cell Biology*, pp 269–287). Moreover, as indicated by the Reviewer, we have indeed used a 20X objective, because of the large size of the CAF cells with elongated protrusions. We have now added these different information in the Methods section (# *Traction Force Microscopy*), **p35.**

Added text: Methods, p35: "These commercial hydrogels contain fluorescent beads of 0.2 µm diameter at a density of 0.2 ± 0.04 bead/µm². 3 CAF-S1 and CAF-S4 pairs were tested. …. 24 h post-plating with 20X objective, the large size of CAF cells with elongated protrusions precluding the use of higher magnification."

In Figure 6e authors reported the strain energy density of CAF-S4 treated with DAPT and a significant difference is observed (p=0.03), However, supplementary figure 6e shows no significant differences (p=0.08) in stress upon DAPT treatment. Authors should specify and discuss in the text that they see a difference in strain and not in stress rather than claiming there is a general difference in traction force generation (lines 398 and 399 of the text).

Indeed, the Strain Energy Density (SED) and total stress magnitude (σ) are different quantities probing the mechanics of the cells. σ integrates the forces locally exerted by the cells (via their Focal Adhesions) while the SED is a more global quantity related to the mechanical work dissipated by the whole cell. Therefore, we favor the SED over the average stress to compare the two situations.

We thus propose to change the sentence: « Using TFM, we confirmed that DAPT treatment also diminished CAF-S4 traction forces exerted on the matrix (Fig. 6e) » by « Using TFM, we confirmed that DAPT treatment also diminished the Strain Energy Density globally exerted by CAF-S4 cells (Fig. 6e), although it did not significantly affect the magnitude of the stress, which averages the local traction forces (Fig. S6e).” This sentence has been added **p15** in the new text.

Added text: *Results, p15:* “Using TFM, we confirmed that DAPT treatment also diminished the Strain Energy Density globally exerted by CAF-S4 cells (Fig. 6e), although it did not significantly affect the magnitude of the stress, which averages the local traction forces (Fig. S6e).”

About the SHG experiments:
The quantification of the collagen remodelling is somehow confusing. The authors measured the percentage area of collagen, but the cells seem to be embedded in collagen gels (2.5 mg/ml collagen solution 926 (Corning, #354249)). This may not be a good measure of collagen remodelling. Other metrics like alignment or density may be more adequate. I also do not understand how they calculate the median per cell (I would expect each cell to be a data point, are they calculating the percentage of collagen for several windows around the cell? Is it the median when considering the z stack?). The difference is evident from the images, but another descriptor may be much more robust than collagen area.

We are sorry for the confusion. As requested, we have now quantified the density of collagen around each cell in the **new Figure 4i (Right).** The calculation of the density is now described in the **Methods** section *(# Collagen evaluation by Second Harmonic Generation),* **p36**. Accordingly, we have modified the description of the results in **Figure 4i, p11**, and the Figure legend, **p55** of the new text.

We would like also to clarify that median was initially chosen because the analysis was done in 3D, stack by stack, to cover the volume of the cell. The median was indeed computed considering all slices in the z stack so that in the end one value on the graph would represent the data for one cell. However, we have now replaced this parameter by the density of collagen around each cell, as recommended by the Reviewer. We have now explained the method to compute the density in the **Methods** section, **p36.**

Added text: *Methods, p36:* “Collagen density around each cell was then computed as $d = \frac{\sum_{i=1}^{n} \Delta z \times surf.coll_{slice(i)}}{V_{stack}}$, where Δz is the step between two slices in the stack, *n* the number of slices in the stack, *Vstack* the volume of the stack and $surf.coll_{slice(s)}$ the area covered by collagen in the slice *i* of the stack (Fiji output).”

Added text: *Results, p11*: “we observed a strong increase in collagen fiber density around CAF-S4 compared to CAF-S1 cells (Fig. 4i)”

Added text: *Legend Fig. 4i, p55:* “Right, Collagen density in each cell stack. Each dot represents the value of collagen density around a single cell (n ≥ 10 cells per CAF subset, 2 pairs are shown). p values from Student’s t-test (1st CAF pair) and Mann-Whitney (2nd CAF pair). n = 2 independent experiments.”

The results represented in Figure 4i, do not take into account the volume occupied by the cell. To take into account the variation of cell volume, we have quantified cell volume using the same method as described for collagen. Subtraction of cell volume from the volume occupied by collagen, do not change the result (as shown below).

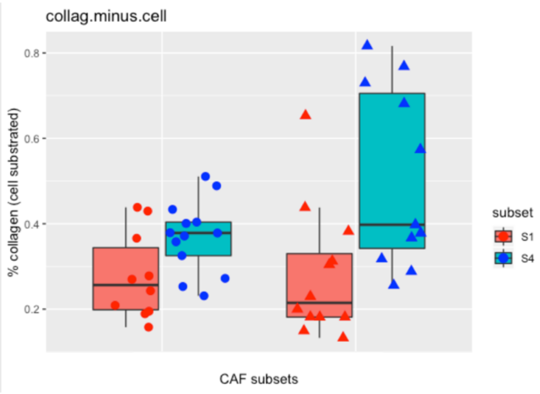

Additional comment:
What do they refer to as CAF-S1 and CAF-S4 pairs? How many cells do they analyse per pair? The authors seem to answer these for some of the figures; I recommend inviting them to put this information in the methods.
CAF pairs represent CAF-S1 and CAF-S4 isolated from the same patient from PT and/or LN. We have now indicated this precision within the Method section **p29.**

__Added text__: *__Methods, p29:__* "All along the duration of the project, we isolated 22 CAF-S1 and 22 CAF-S4 paired primary cell lines (referred to as CAF pairs because isolated from the same patient)".

Reviewers' Comments:

Reviewer #5:
Remarks to the Author:
The authors answered satisfactorily to all points raised and provided compelling arguments for the methods used in their manuscript (e.g. use of 20X for TFM, strain energy density vs total stress). Moreover, the authors expanded on their methods and clarified the points raised in our remarks concerning the TFM and SHG protocols. This reviewer thinks that these additional details make the materials & methods more comprehensive, help readers understand the data, and help other researches reproduce their experiments.
